# Inhibitors of BRAF dimers using an allosteric site

**DOI:** 10.1038/s41467-020-18123-2

**Published:** 2020-09-01

**Authors:** Xiomaris M. Cotto-Rios, Bogos Agianian, Nadege Gitego, Emmanouil Zacharioudakis, Orsi Giricz, Yang Wu, Yiyu Zou, Amit Verma, Poulikos I. Poulikakos, Evripidis Gavathiotis

**Affiliations:** 1 Department of Biochemistry, Albert Einstein College of Medicine, Bronx, NY USA; 2 Department of Medicine, Albert Einstein College of Medicine, Bronx, NY USA; 3 Department of Oncology, Albert Einstein College of Medicine, Montefiore Medical Center, Bronx, NY USA; 4 Department of Developmental & Molecular Biology, Albert Einstein College of Medicine, Bronx, NY USA; 5 Albert Einstein Cancer Center, Albert Einstein College of Medicine, Bronx, NY USA; 6 Department of Oncological Sciences, Icahn School of Medicine at Mount Sinai, New York, USA; 7 Department of Dermatology, Icahn School of Medicine at Mount Sinai, New York, USA

**Keywords:** Kinases, Structural biology, Cell signalling, Small molecules, Target validation

## Abstract

BRAF kinase, a critical effector of the ERK signaling pathway, is hyperactivated in many cancers. Oncogenic BRAF^V600E^ signals as an active monomer in the absence of active RAS, however, in many tumors BRAF dimers mediate ERK signaling. FDA-approved RAF inhibitors poorly inhibit BRAF dimers, which leads to tumor resistance. We found that Ponatinib, an FDA-approved drug, is an effective inhibitor of BRAF monomers and dimers. Ponatinib binds the BRAF dimer and stabilizes a distinct αC-helix conformation through interaction with a previously unrevealed allosteric site. Using these structural insights, we developed PHI1, a BRAF inhibitor that fully uncovers the allosteric site. PHI1 exhibits discrete cellular selectivity for BRAF dimers, with enhanced inhibition of the second protomer when the first protomer is occupied, comprising a novel class of dimer selective inhibitors. This work shows that Ponatinib and BRAF dimer selective inhibitors will be useful in treating BRAF-dependent tumors.

## Abstract (web summary)

FDA-approved RAF inhibitors poorly inhibit BRAF dimers, which limits their clinical efficacy in tumors expressing BRAFV600E mutant monomers. Here the authors identify FDA-approved Ponatinib as an effective inhibitor of BRAF monomers and dimers and designed PHI1, an inhibitor with a unique mode of action and selectivity for oncogenic BRAF dimers.

## Introduction

The RAS–RAF–MEK–ERK signaling pathway (ERK signaling) regulates mammalian cell growth, proliferation, and survival^1,2^. This pathway is normally activated by receptor tyrosine kinase signaling that stimulates binding of GTP to RAS at the plasma membrane^3,4^. RAF proteins (ARAF, BRAF, and CRAF isoforms) are subsequently recruited at the membrane by interaction with the active GTP-bound RAS, where they are activated by dephosphorylation and phosphorylation events and simultaneous dimerization of their RAF kinase domains^1,2,5^. Activated RAF proteins subsequently initiate a cascade of phosphorylation and activation steps of downstream kinases MEK1/2 and ERK1/2, which then phosphorylate an array of proteins leading to specific cellular effects^1,2,5^.

Aberrant activation of ERK signaling is a hallmark of many cancers most commonly due to mutations of RAS and BRAF^6,7^. BRAF mutants are found in up to 9% of all human cancers and over 50% of melanoma^8,9^. While RAF proteins activate ERK signaling as homo and heterodimers in the presence of active RAS^10^, mutant BRAF^V600E^ can activate ERK signaling independent of RAS as an active monomer^11,12^. Drug development efforts have yielded three FDA-approved RAF inhibitors, Vemurafenib, Dabrafenib, and Encorafenib for BRAF^V600E^ metastatic melanoma^13,14^. These inhibitors show remarkable clinical efficacy in BRAF^V600E^ melanoma tumors from potent inhibition of the monomeric BRAF^V600E^ protein^11–14^. However, drug resistance to these inhibitors is developed resulting in only short-term improvement of patients’ survival^15^. Several mechanisms of clinical resistance to RAF inhibitors have been identified, including feedback reactivation of receptor tyrosine kinases and RAS, RAS mutations, BRAF amplification and expression of BRAF^V600E^ splice variants^16–19^. These resistance mechanisms commonly lead to reactivation of ERK signaling through dimerization of RAF proteins^16–19^. RAF dimers are poorly inhibited by the FDA-approved inhibitors^11,12^ and therefore have been recognized as an important target for limiting drug resistance but also for more effective inhibition of ERK signaling in various tumors^20,21^.

Recently, next-generation RAF inhibitors that equipotently inhibit RAF monomers and dimers have been developed^11–14^. Comperative analysis of crystal structures of BRAF kinase bound to RAF inhibitors showed that inhibitors that stabilized the αC-helix toward the inactive OUT position (αC-OUT inhibitors), such as Vemurafenib, Dabrafenib and PLX7094, sterically disfavor binding of the inhibitor to the second protomer within the RAF dimer (negative allostery)^12,22,23^. In contrast, RAF inhibitors that stabilized the αC-helix toward the active IN position (αC-IN inhibitors), such as TAK-632, LY3009120, and AZ-628, favor catalytic inhibition of both RAF protomers within the dimer by two inhibitor molecules^12,24,25^. Biochemical data consistently demonstrated that αC-IN inhibitors are more effective in inhibiting dimeric RAF activity compared to αC-OUT inhibitors^11,12^. However, αC-IN inhibitors strongly promote RAF binding to active RAS (RAF priming) and therefore induce increased RAF dimerization. In contrast, αC-OUT inhibitors only weakly promote RAF priming^12,26^. Nevertheless, with increased active RAS levels, αC-OUT inhibitors can induce RAF priming and RAF dimerization resulting to the phenomenon described as inhibitor-induced paradoxical activation. This is due to the inability of αC-OUT inhibitors to effectively inhibit both protomers, but inhibit only one protomer, within the RAF dimer^27–29^. Therefore, negative allostery and induced RAF priming are two allosteric mechanisms that hamper both αC-OUT and αC-IN RAF inhibitors, underscoring the need for improved rational designed strategies to effectively target BRAF.

Interestingly, although αC-OUT inhibitors show significant selectivity for active BRAF^V600E^ monomers compared to BRAF-mutant or wild-type dimers, αC-IN inhibitors inhibit BRAF monomers and dimers or each protomer within a RAF dimer equipotently^11,12,26^. Lack of selectivity by αC-IN inhibitors can significantly reduce their therapeutic index of inhibiting mutant RAF dimers in cancer cells compared to inhibiting wild-type RAF dimers in normal cells^12^. Notably, LY3009120, a potent RAF dimer αC-IN inhibitor, showed limited dose escalation and at its maximum tolerated dose demonstrated limited efficacy in recent phase I trial^30^. Thus, we hypothesized that selective RAF dimer inhibitors may be superior for inhibition of resistant RAF dimers in tumors. Here, we screened a panel of kinase inhibitors with diverse structures in a cell-based assay that probed the activity of obligated BRAF^V600E^ dimers^19^. We found that the FDA-approved drug Ponatinib is a potent BRAF inhibitor. Ponatinib inhibited ERK signaling activity in cancer cells driven by either BRAF monomers or dimers. Structural analysis demonstrated that Ponatinib, in contrast to other RAF inhibitors, binds BRAF forming interactions with an allosteric site. Using these structural insights, we developed Ponatinib Hybrid Inhibitor 1 (PHI1) that displays cellular selectivity for BRAF^V600E^ and non-V600E dimers over monomers via enhanced inhibition of the second protomer within the dimers, when the first protomer is occupied. Structural investigation revealed that PHI1 recognizes a previously uncharacterized allosteric site in RAF kinases, which is intimately linked to the conformation of αC-helix. Our studies present mechanistic insights and BRAF inhibitors that can be exploited for the treatment of BRAF dimer-dependent tumors.

## Results

### Screening for RAF dimer kinase inhibitors

**Fig. 1:**
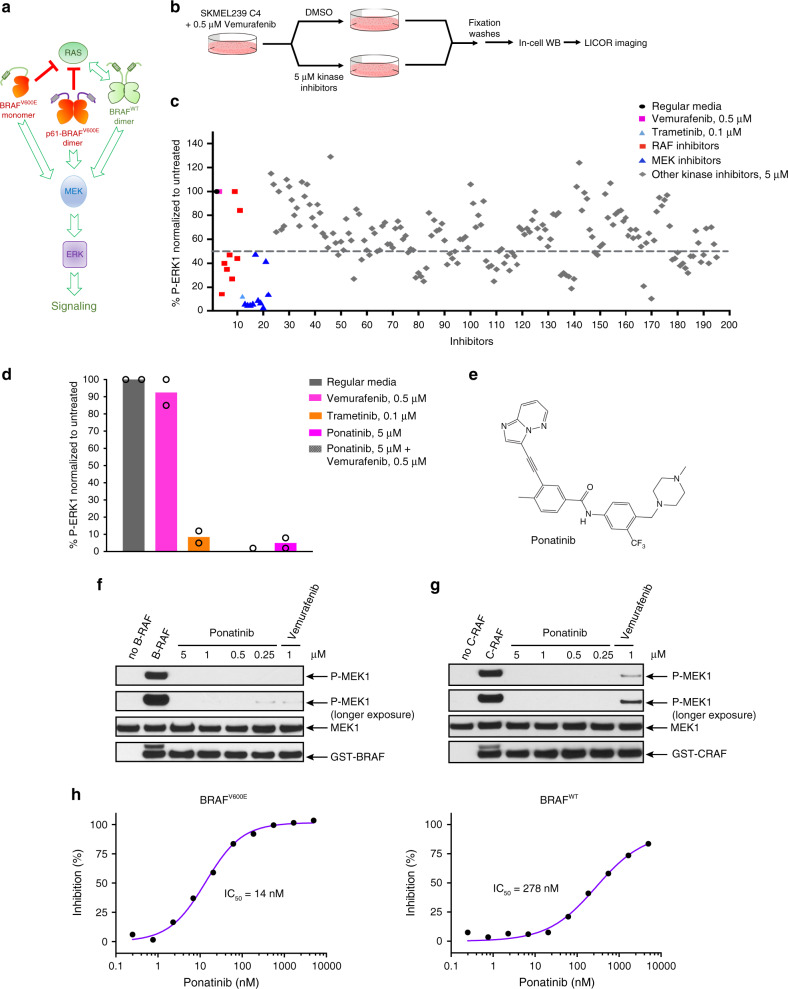
Ponatinib is a RAF inhibitor. **a** Schematic representation of ERK signaling pathway from different BRAF species. **b** Schematic representation of the in-cell-western screening assay. **c** Screening of a library of 200 kinase inhibitors using in-cell-western assay. SKMEL239-C4 melanoma cells left untreated (regular media), treated with 0.5 μM Vemurafenib, 0.1 μM Trametinib, or with 5 μM of known RAF, MEK, and other kinase inhibitors in the presence of 0.5 μM Vemurafenib for 3 h. **d** SKMEL239-C4 melanoma cells left untreated (regular media), treated with 0.5 μM Vemurafenib, 0.1 μM Trametinib, Ponatinib 5 μM without or with 0.5 μM Vemurafenib for 3 h, and assayed with in-cell-western. Data are mean of *n* = 2 independent experiments. **e** Chemical structure of Ponatinib. **f** BRAF kinase activity assay in the absence or presence of Ponatinib or Vemurafenib was assayed by western blot with the indicated antibodies. **g** CRAF kinase activity assay in the absence or presence of Ponatinib or Vemurafenib was assayed by western blot with the indicated antibodies. **g** Kinase activity inhibition profiles of BRAF^V600E^ and BRAF^WT^ upon Ponatinib titration using SelectScreen assay. Data are mean of two technical replicates from *n* = 2 independent experiments. Source data are provided as a Source Data file.

To identify RAF dimer kinase inhibitors, we established an in-cell-western based screening assay using SKMEL239-C4 melanoma cells^31^. These cells were generated under Vemurafenib-induced selection pressure inhibiting BRAF^V600E^, allowing preferential growth of cells expressing p61BRAF^V600E^, a splice variant of BRAF that constitutively signals as a dimer in a RAS-independent manner^19^ (Fig. 1a). p61BRAF^V600E^ is resistant to Vemurafenib and is found in patients’ tumors^19^. Typically, Vemurafenib robustly inhibits ERK signaling in BRAF^V600E^ expressing cells such as melanoma A375 cells at 0.3 μM, whereas in SKMEL239-C4 cells a similar effect required over 10 μM (Supplementary Fig. 1a, b)^11,12^. The in-cell-western assay, which was based on fluorescence readout for phosphorylated ERK (p-ERK) and total ERK levels, enabled a wide dynamic range of signal detection and recapitulated p-ERK resistance of SKMEL239-C4 cells at low concentrations of Vemurafenib (Supplementary Fig. 1c, d). In contrast, Trametinib, an FDA-approved MEK inhibitor, potently inhibited ERK signaling at low nM concentrations (Supplementary Fig. 1e). We used a 96-well format with non-inhibitory concentrations of Vemurafenib as negative control and 0.1 μM Trametinib as positive control, obtaining a *Z*-factor of 0.68 (Supplementary Fig. 1f).

We screened a library of 200 kinase inhibitors, including established RAF and MEK inhibitors as positive controls, and found several additional compounds with capacity to reduce p-ERK levels by more than 50% at 5 μM after 3 h of treatment (Fig. 1b, c). SKMEL239-C4 cells were co-treated with 0.5 μM Vemurafenib in order to saturate inhibition of low expressing BRAF^V600E^ monomer and reveal inhibition of p61BRAF^V600E^ dimer^19^. Hits were screened again with or without Vemurafenib co-treatment as control. Several kinase inhibitors were confirmed to have potent inhibitory effect on p-ERK, independently of Vemurafenib (Supplementary Fig. 2). Since p61BRAF^V600E^ maintains activity in SKMEL239-C4 cells independently of active RAS or tyrosine kinase receptor signaling, our screening assay identified potential BRAF dimer inhibitors^12,19^ (Supplementary Fig. 2). Ponatinib induced one of the strongest inhibitory effect on p-ERK (Fig. 1d, e).

### Ponatinib is a potent BRAF inhibitor

Following validation of hits, we focused on Ponatinib, which is an FDA-approved kinase inhibitor for chronic myeloid leukemia (CML) targeting BCR-ABL-T315I mutant^32,33^. Besides ABL in CML, Ponatinib strongly inhibits other kinases such as FGFR, FLT3, KIT, and PDFGRα (Supplementary Fig. 3)^34–36^ and it was previously reported in kinome screens to inhibit BRAF activity, however, it is not validated or recognized as BRAF inhibitor^13,37^. We performed in vitro kinase assays for BRAF^V600E^ activity to phosphorylate recombinant MEK and for MEK1 activity to phosphorylate recombinant ERK2, respectively. We found that Ponatinib is a potent inhibitor of MEK phosphorylation by BRAF^V600E^, but does not inhibit MEK (Fig. 1f and Supplementary Fig. 4). In addition, Ponatinib potently inhibited CRAF (Fig. 1g). Ponatinib inhibited BRAF^V600E^ (IC_50_ = 14 nM) and BRAF^WT^ (IC_50_ = 278 nM) comparably to Vemurafenib, Dabrafenib, and Encorafenib inhibitors but also to LY3009120, AZ-628, and TAK-632 that inhibit both monomers and dimers (Fig. 1h and Supplementary Fig. 5)^11,12^. Thus, our data demonstrate that Ponatinib is an inhibitor of BRAF with similar potency to other RAF inhibitors.

### Ponatinib binds BRAF with a unique binding mode

**Fig. 2:**
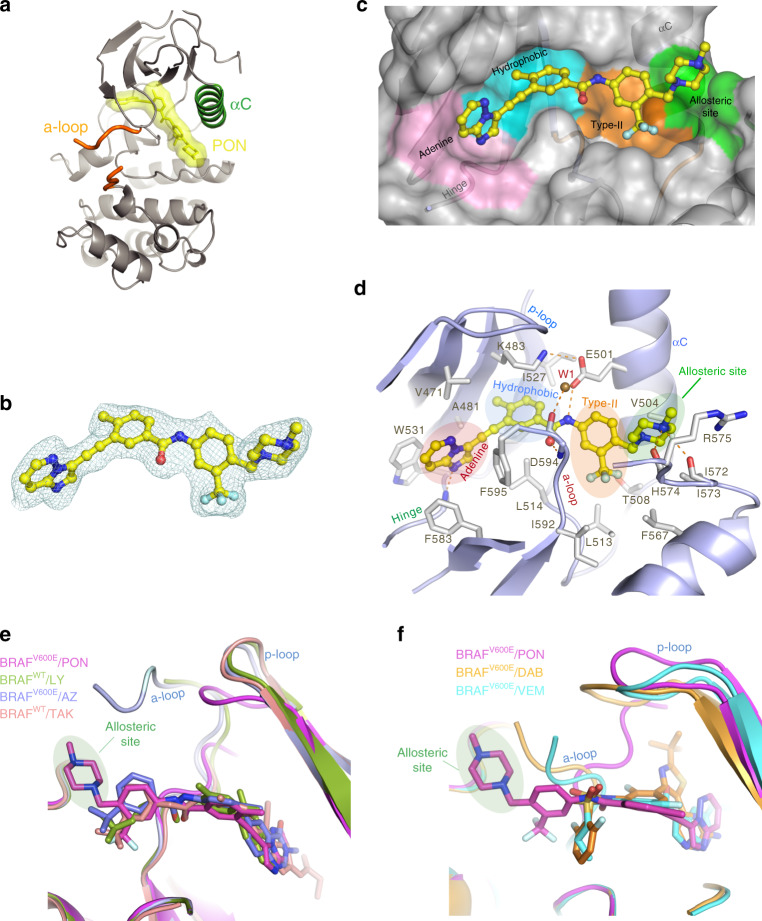
Structural analysis of BRAFV600E bound to Ponatinib (PON). **a** Crystal structure of the BRAF^V600E^/PON protomer structure. BRAF^V600E^ structure depicted in ribbons (gray) showing the αC-helix (green). The partially disordered activation loop (orange) is shown in a tube representation. PON is shown in sticks and molecular surface (yellow). **b** *F*_o_ – *F*_c_ electron density of PON in the BRAF^V600E^/PON complex contoured at 3*σ*. **c** Close-up of PON binding (in ball-and-sticks) to its binding pocket (transparent surface) in BRAF^V600E^. Protein sub-pockets recognizing various inhibitor moieties are colored, including the allosteric pocket (green). **d** A close-up view of PON binding interactions with BRAF^V600E^. PON is shown in ball-and-sticks and BRAF^V600E^ in a cartoon model. Protein residues interacting with PON are shown in sticks. Note hydrogen bond interactions of PON with the backbone of residues H574 and I573 in the allosteric site and interaction with the D594 and F595 of the DFG motif and catalytic E501 of the αC-helix. A structural water molecule in the binding site (W1) is shown as a sphere. H-bonds are shown in dashed lines. **e** A superposition of BRAF type-II/αC-IN inhibitors LY3009120 (LY, green, PDB 5C9C), TAK-632 (TAK, orange, PDB 4KSP), and AZ-628 (AZ, blue, PDB 4G9R) bound in their BRAF recognition pockets with PON in BRAF^V600E^/PON structure. Protein parts were omitted from clarity. **f** A superposition of BRAF type-Ib/αC-OUT inhibitors Vemurafenib (VEM, cyan, PDB 3OG7) and Dabrafenib (DAB, orange, PDB 4XV2) bound in their BRAF recognition pockets with PON in BRAF^V600E^/PON structure. Inhibitors are depicted in sticks and proteins in a cartoon model.

To gain further insights into the mechanism of BRAF inhibition by Ponatinib, we determined the BRAF^V600E^-Ponatinib co-crystal structure at 2.1 Å resolution (Supplementary Table S1). In this complex, Ponatinib is well-defined in electron density and binds deeply into the BRAF inter-lobe active site cleft (Fig. 2a, b). Ponatinib induces the DFG-OUT conformation and forms an extended network of hydrophobic and polar contacts, reflecting its nanomolar activity (Fig. 2c, d). Its pyridazine group establishes hydrogen bonds to hinge residues and the methyl and trifluoromethyl moieties occupy the hydrophobic and type-II pockets, respectively. The amide linker forms additional hydrogen bonds to the catalytic E501 residue, which however maintains the catalytically important salt bridge with K483 (Fig. 2d)^9^, a signature of active BRAF conformation. Interestingly, the activation loop of the kinase domain is partially disordered (Fig. 2a)^12,38^, whereas, its experimentally determined portion adopts an orientation that docks residue E600 to a unique position compared to other inhibitor-bound BRAF^V600E^ structures (Supplementary Fig. 6), most likely a result of Ponatinib binding mode.

Further analysis revealed that Ponatinib adopts a unique recognition mode for a RAF inhibitor. In this binding mode, apart from the previously defined “adenine”, “hydrophobic”, and “type-II” pockets (Fig. 2c, d), Ponatinib forms extensive interactions with an allosteric site (Fig. 2c, d) defined as back-pocket IV (BP-IV) according to Kinase-Ligand Interaction Fingerprints and Structures (KLIFS) database^39^. Indeed, a comparison of Ponatinib binding mode to known BRAF inhibitors illustrates distinct recognition of this allosteric site by the methyl-piperazine moiety of Ponatinib (Fig. 2e, f). These interactions include a bifurcated hydrogen bond with the backbone carbonyl oxygen atoms of αΕ-β6 loop residues I573 and H574 and hydrophobic contacts with R575 (Fig. 2d). Interestingly, H574 and R575 belong to the conserved HRD motif that is essential for substrate phosphorylation^40^ (Fig. 2d). R575 recognizes regulatory phosphorylated residues of the activation loop and the highly conserved H574 is part of the kinase regulatory spine (R-spine)^40^. Although Ponatinib binds Receptor Tyrosine Kinases (RTKs) in a similar manner (see below), among RAF inhibitors, HRD recognition is achieved only by Ponatinib. Taken together, our structural analysis suggests that Ponatinib binding to BRAF involves interactions with a previously unreaveled allosteric site.

### Ponatinib induces BRAF dimers with αC-CENTER conformation

Crystal structures of BRAF with inhibitors typically are determined in dimeric conformation and each protomer’s αC-helix can adopt different conformations between the IN (active) and OUT (inactive) position. αC-IN inhibitors occupy both protomers within the dimer, which both have an αC-IN conformation. In contrast, αC-OUT inhibitors occupy the first protomer with the αC-OUT conformation and induce the second protomer with an αC-IN conformation, therefore inhibiting the binding of a second αC-OUT inhibitor. Differences in αC conformation between dimers result in asymmetry as shown by the superposition of protomers, which is more pronounced with αC-OUT inhibitors (VEM, DAB) and less with αC-IN inhibitors (LY3009120, AZ-628, and TAK-632)^12^ (Supplementary Fig. 7).

**Fig. 3:**
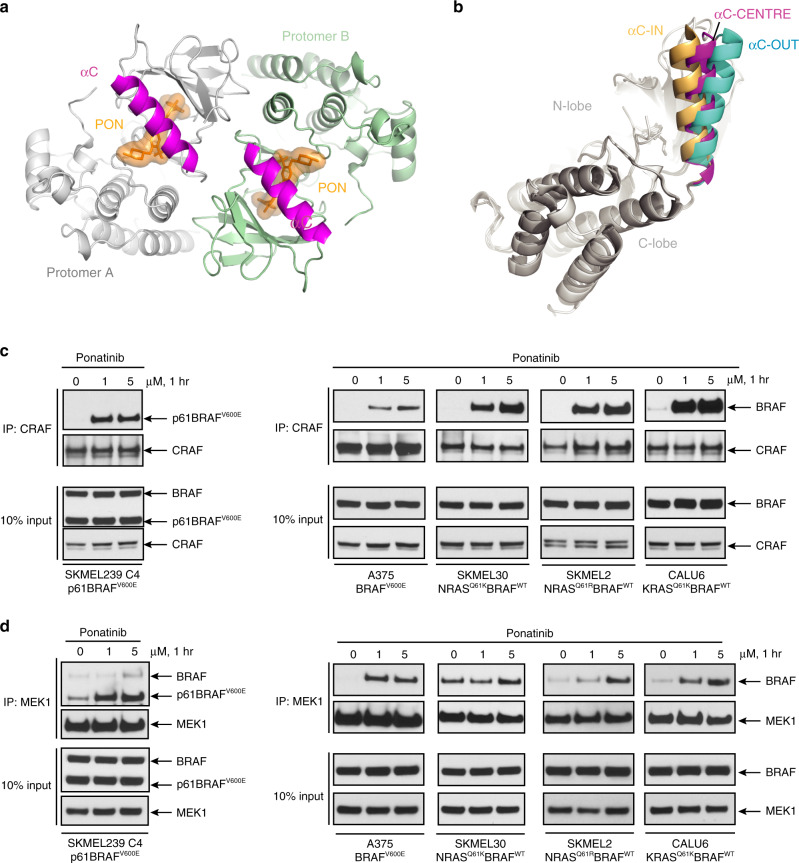
Ponatinib induces symmetrical BRAF dimers and promotes BRAF complexes in cells. **a** The BRAF^V600E^/PON symmetrical dimer structure. The dimer is viewed along its twofold axis with two protomers in silver and green ribbon representations. PON bound to each protomer is shown in orange sticks/molecular surface. The αC-helix of each protomer is illustrated in magenta. **b** The αC-helix of BRAF^V600E^/PON complex adopts a αC-CENTRE position. Structural superposition of BRAF protomer structures in ribbon representation showing that αC-helix of BRAF-PON structure (magenta) lies between typical αC-OUT position observed in BRAF–Vemurafenib complex (cyan, PDB 3OG7) and αC-IN position in MEK-bound BRAF (gold, PDB 4MNE). Protein parts were omitted for clarity. **c** Various cell lines expressing BRAF^V600E^ or RAS^MUT^/BRAF^WT^ were left untreated or treated with 1 or 5 μM Ponatinib for 1 h. Cells were then collected, assayed for CRAF by immunoprecipitation and immunoblot with the indicated antibodies for monitoring BRAF/CRAF heterodimerization and activation of ERK signaling. **d** Similar analysis to **c** assayed for MEK by immunoprecipitation and immunoblot with the indicated antibodies for monitoring BRAF/MEK complex formation. Data are representative of *n* = 3 independent experiments. Source data are provided as a Source Data file.

The BRAF–Ponatinib structure corroborates the dimeric conformation of the kinase induced by αC-IN inhibitors, with both protomers occupied by Ponatinib (Fig. 3a). Interestingly, Ponatinib induces an intermediate αC-CENTRE position in both protomers (Fig. 3b and Supplementary Fig. 8a), which compose a perfectly symmetric dimer (crystallographic dimer). To further investigate whether the BRAF–Ponatinib complex has a distinct conformation among other Ponatinib-kinase complexes, we compared the orientation of αC-helix in this complex to crystal structures of Ponatinib with ABL, ABL-T315I, FGFR1, FGFR4, and cKIT kinases^35–37^ (Supplementary Fig. 8b). Comparison shows that only the αC-helix in the BRAF–Ponatinib adopts the αC-CENTER position, despite the similar binding mode of Ponatinib in all active sites (Supplementary Fig. 8c).

To determine whether Ponatinib also induces BRAF dimers in solution, we performed size-exclusion chromatography analysis of recombinant BRAF kinase in complex with Ponatinib and compared its migration to apo-BRAF kinase and BRAF kinase complexed to ATP. As shown in Supplementary Fig. 9, stoichiometric amounts of Ponatinib promote formation of BRAF dimers, in contrast to ATP, which produces BRAF monomers. Next, using co-immunoprecipitation assays, we evaluated whether Ponatinib can promote dimerization of endogenous BRAF with CRAF in cell lines expressing BRAF variants such as BRAF^V600E^, p61BRAF^V600E^, and BRAF^WT^/NRAS mutant. Consistent with our in vitro data, Ponatinib-promoted formation of BRAF^V600E^, p61BRAF^V600E^, and BRAF^WT^ dimers with CRAF in all cell lines in a dose-dependent manner (Fig. 3c). Moreover, co-immunoprecipitation assays revealed that Ponatinib enhanced the formation of RAF/MEK complexes in all cell lines in a dose-dependent manner, suggesting that Ponatinib-induced BRAF dimers have a conformation compatible for stable interaction with MEK^41^ (Fig. 3d). Taken together, our data suggest that Ponatinib binds either BRAF^V600E^ monomers or p61BRAF^V600E^ and BRAF^WT^ dimers and promotes and stabilizes BRAF dimers and RAF/MEK complexes.

### Ponatinib effectively inhibits oncogenic BRAF

**Fig. 4:**
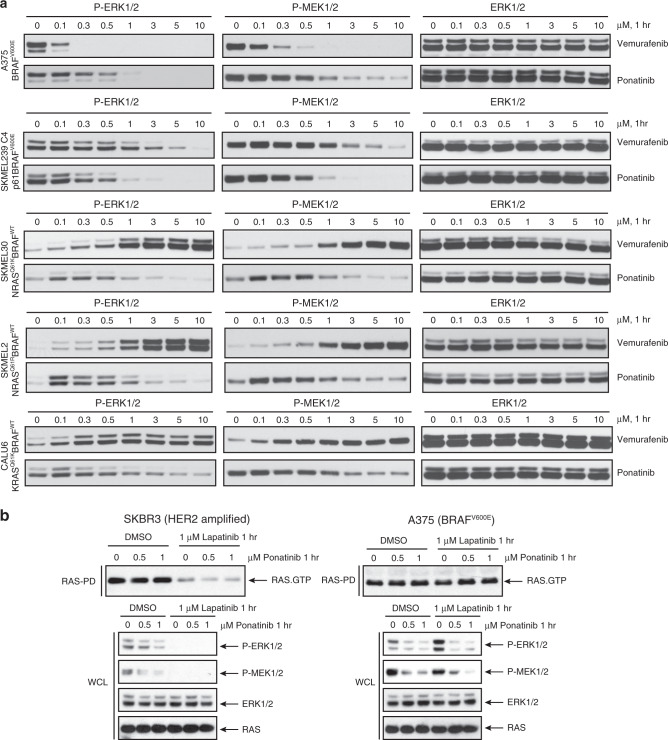
Ponatinib inhibits different BRAF species and ERK signaling in cells. **a** Melanoma A375, SKMEL239-C4, SKMEL-30, SKMEL-2, and lung cancer CALU6 cell lines expressing BRAF^V600E^ or RAS^MUT^/BRAF^WT^ left untreated or treated with increasing concentration of Ponatinib or Vemurafenib for 1 h, then assayed for western blot and immunoblot with the indicated antibodies to probe ERK-signaling inhibition. Data are representative of three independent experiments. **b** Breast cancer SKBR3 cells, which are RTK-dependent HER2 amplified and melanoma A375 (BRAF^V600E^ monomer) cells were left untreated or treated with increasing concentration of Ponatinib and Lapatinib, an EGFR/HER2 inhibitor, for 1 h, then assayed by immunoblot with the indicated antibodies. Data are representative of *n* = 3 independent experiments. Source data are provided as a Source Data file.

Next, we sought to examine the capacity of Ponatinib to inhibit ERK signaling in tumor cells dependent on BRAF^V600E^, p61BRAF^V600E^ and RAS^MUT^/BRAF^WT^. For comparison with a potent inhibitor of BRAF^V600E^ monomers we used Vemurafenib. Ponatinib inhibited ERK signaling in BRAF^V600E^ and p61BRAF^V600E^ dependent melanoma cells at 0.3–0.5 μΜ doses. However, it required 1–3 μΜ dose to effectively inhibit signaling in RAS^MUT^/BRAF^WT^ melanoma or lung cancer cells, suggesting some resistance due to RAF priming similarly to other αC-IN inhibitors^11,12^ (Fig. 4a). In contrast, Vemurafenib inhibited only BRAF^V600E^ monomer expressing cells (A375) but lacks significant inhibitory activity of p-ERK in cells dependent on p61BRAF^V600E^ dimers and enhances p-ERK levels in RAS^MUT^/BRAF^WT^ cells, due to significant negative allostery^11–13^ (Fig. 4a).

To further confirm the direct activity of Ponatinib on BRAF in inhibiting ERK signaling against its potential activity on upstream RTK targets, we used a model system that allows us to assess ERK signaling by RTK inhibition in the absence of active BRAF or RAS-GTP mutations. Thus, we used SKBR3 breast cancer cell line, in which HER2-activated RAS^WT^-GTP levels are attenuated by Lapatinib (dual EGFR/HER2 tyrosine kinase inhibitor) treatment^11^. Ponatinib inhibited ERK signaling without reducing RAS^WT^-GST levels in SKBR3 cells, whereas Lapatinib inhibited ERK signaling and significantly reduced RAS^WT^-GST levels, as expected (Fig. 4b). To test whether Ponatinib affects RAS^WT^-GTP levels in an RTK-independent manner, we used A375 cells. Both Ponatinib and Lapatinib did not reduce RAS^WT^-GTP levels, however Ponatinib only inhibited ERK signaling suggesting direct inhibition of BRAF^V600E^ (Fig. 4b). We also confirmed BRAF inhibition by Ponatinib in HEK293 cells whereby ERK signaling was dependent on heterologous expressed p61BRAF^V600E^ (Supplementary Fig. 10). These results suggest that Ponatinib targets BRAF irrespective of RTK targets.

Lastly, we tested the efficacy of Ponatinib to inhibit tumor growth in melanoma cells dependent on BRAF^V600E^, p61BRAF^V600E^, and RAS^MUT^/BRAF^WT^ grown in 3D culture conditions as previously reported^42^. Ponatinib demonstrated increased efficacy to inhibit colony formation as compared to Vemurafenib in tumor cells expressing BRAF^V600E^ monomers, BRAF^V600E^ dimers and RAS^MUT^/BRAF^WT^ dimers (Supplementary Fig. 11). Taken together, these data suggest that Ponatinib is an effective BRAF inhibitor that inhibits melanoma cells dependent on BRAF^V600E^ monomers and dimers as well as mutant RAS-activated BRAF^WT^ dimers with less potency.

### Exploiting the BRAF allosteric site with a designed inhibitor

Based on the structural insights of the Ponatib-BRAF^V600E^ dimer structure, we aimed to further exploit the identified BP-IV allosteric site. We performed a computational structure-based drug design approach to replace the head group of Ponatinib structure with alternative fragments that keep the interactions of the trifluoro-phenyl and piperazine interactions of Ponatinib while extending the compound deeper in the allosteric site (Supplementary Fig. 12a, b). Candidate compounds were evaluated in silico for the interactions with the site and filtered for favorable properties compared to Ponatinib (Supplementary Fig. 12a, b and see “Methods” section). Using this approach, we generated PHI1, which uses a morpholine-based head group predicted to interact proximal to the BP-IV site (Supplementary Fig. 12c, d and Supplementary Methods). Indeed, PHI1 showed high inhibitory potency IC_50_ = (10 nM) against BRAF^V600E^ kinase activity (Supplementary Fig. 13).

**Fig. 5:**
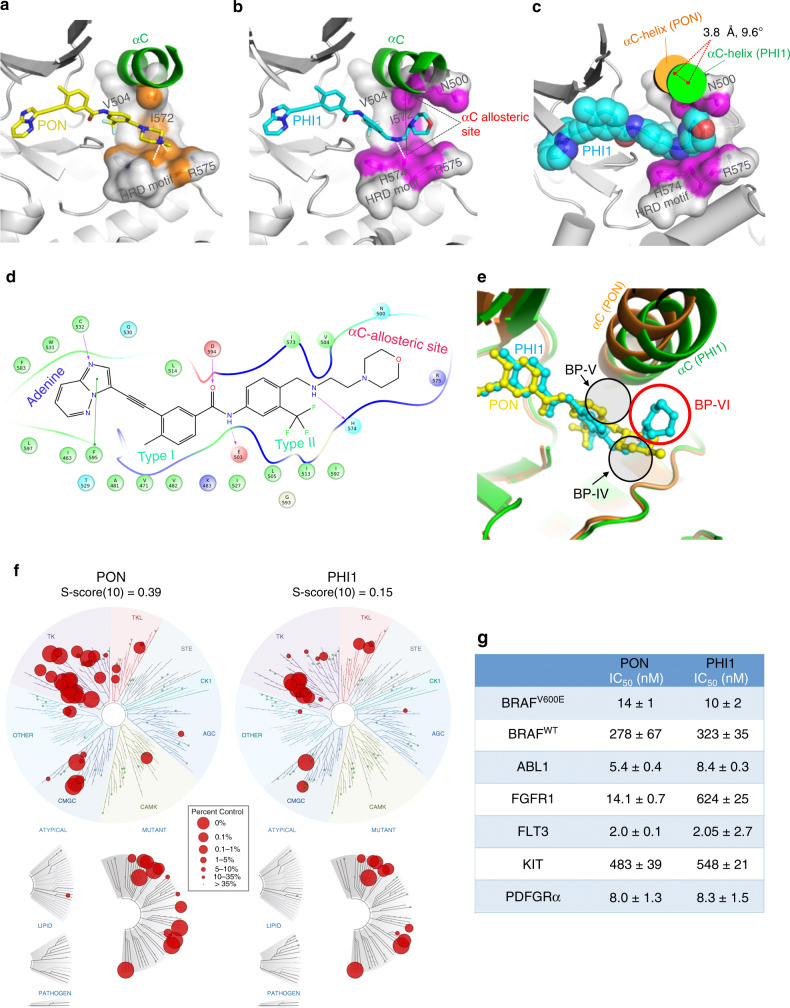
Targeting the allosteric site in BRAF with PHI1. Comparison of binding modes of PON (**a**) and PHI1 (**b**) with BRAF^V600E^ in their corresponding crystal structures, illustrating interactions of PHI1 with the αC-allosteric site. Protein residues lining the base of αC-helix and HRD motif are shown in surface representation. Protein–ligand interface contact area (within 3.6 Å of ligand atoms) is colored in orange (PON) and magenta (PHI1), respectively. H-bonds are shown with dashed white lines. Protein parts are omitted for clarity. **c** Major PHI1-induced structural rearrangement of αC-helix (green) in BRAF^V600E^/PHI1 complex compared to BRAF^V600E^/PON (orange). The αC-allosteric site is shown in surface representation as in **b** and PHI1 atoms as van der Waals spheres. **d** Cartoon map showing molecular interactions between PHI1 and BRAF^V600E^. **e** Superposition of BRAF^V600E^/PHI1 (green) and BRAF^V600E^/PON (orange) complexes, demonstrates that PHI1 binding defines a new back pocket (BP-VI, red circle), according to KLIFS database nomenclature, which corresponds to the aC-allosteric site shown in **b**. PON is classified as a BP-IV binder. The position of BP-V is also shown. **f** KinomeEDGE^®^ of PON and PHI1 at 1 μM. Interaction maps of human kinases, including mutated, pathogen and lipid kinases, illustrating levels of PON and PHI1 binding (radii of red circles, see inset) giving better than 90% displacement of control binding (see “Methods” section). **g** Inhibition of kinase activity of BRAF^V600E^, BRAF^WT^, and other tyrosine kinases targets, identified by KinomeEDGE^®^, using SelectScreen (Invitrogen) in the presence of 100 μM ATP. Half-maximal inhibition values (IC_50_ ± SD) of two technical replicates from *n* = 2 independent experiments are tabulated. Source data are provided as a Source Data file.

To investigate the structural basis of BRAF inhibition by PHI1, we determined the co-crystal structure of PHI1 with BRAF^V600E^ at 2.65 Å resolution (Supplementary Table S1). Comparison of BRAF^V600E^-Ponatinib and BRAF^V600E^–PHI1 structures demonstrated a major allosteric rearrangement induced by PHI1 binding promoting an inward shift of αC-helix (Supplementary Fig. 14). This PHI1-induced BRAF conformational change is compatible with kinase domain dimerization, as a BRAF dimer with two PHI1-bound protomers is found in the BRAF^V600E^–PHI1 crystal structure. Stabilization of BRAF kinase dimer by PHI1 is also observed in solution (Supplementary Fig. 9). Interestingly, this PHI1-dependent allosteric modulation stabilizes the αC-helix in a tilted αC-CENTER conformation, distinct from αC-ΙΝ or αC-OUT conformations but closer to αC-IN, which we termed αC-ΙΝ* (αC-INstar) (Supplementary Fig. 15). This structural change is the result of positioning of the morpholino-based head group of PHI1 in a previously unrevealed extended allosteric BRAF site, which we named αC-allosteric site (Fig. 5a–d). Ponatinib uses its methyl-piperazine head group to interact with the HRD motif (Fig. 5a). In contrast, PHI1 positions the morpholino-based head group between the HRD motif and αC-helix, having van der Waals contacts with HRD residue R575 and residues N500 and V504 of αC-helix that result in additional 38 A^2^ of buried solvent-accessible surface area (Fig. 5b–d). In addition, the specific hydrogen bond between the nitrogen of ethyl-amino linker of PHI1 and the main-chain carbonyl oxygen of H574 is maintained (Fig. 5b–d). Interestingly, comparison between the binding modes of kinase inhibitors across the RAF kinase family using the KLIFS database^38^ suggested that this pocket is distinct from previously reported back- or allosteric pockets (BP-IV or BP-V) and would classify as BP-VI category (Fig. 5e). Notably, this pocket is not predicted in a recent atlas of potential allosteric sites across the kinome^43^.

To evaluate whether the interactions of PHI1 with the BRAF αC-allosteric site can confer selectivity to BRAF compared to other kinases, we compared Ponatinib and PHI1 against a panel of human kinases included in the KinomeEDGE screen (Fig. 5f and Supplementary Table S2). PHI1 demonstrated a significant gain in overall specificity compared to Ponatinib determined by the Specificity Index S(10) (Fig. 5f), which is comparable to several selective clinical kinase inhibitors^44^. To further validate the results of the kinome screen, we tested PHI1 against established targets of Ponatinib using the SelectScreen assay. Consistently, PHI1 had reduced inhibitory activity in several targets, with FGFR (43-fold reduction) and FLT3 (tenfold reduction) having the highest differences (Fig. 5g). Taken together, our results demonstrated that specific structural changes to BRAF conformation and interactions of PHI1 with the αC-allosteric site resulted in increased specificity of PHI1 for BRAF against other kinases.

### PHI1 is selective for oncogenic BRAF dimers

**Fig. 6:**
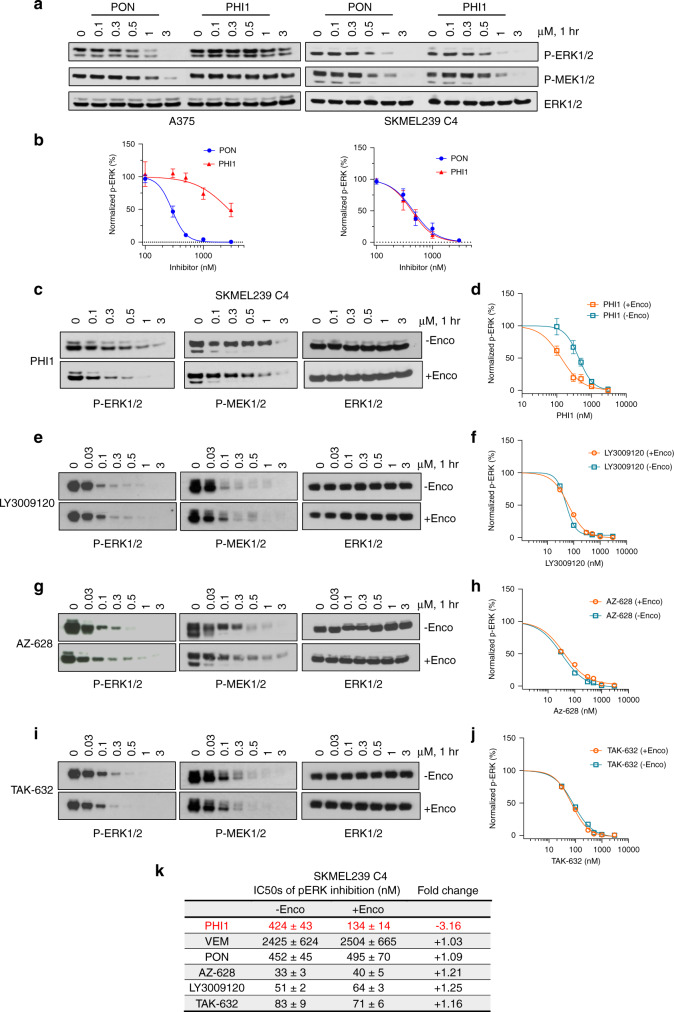
PHI1 is a selective BRAF dimer inhibitor that displays positive co-operativity. **a** Melanoma A375 and SKMEL239-C4 cell lines were treated with increasing. concentrations of PON or PHI1 for 1 h. Whole-cell lysate were assayed by western blot with the indicated antibodies to assess ERK-pathway inhibition. Representative blots from *n* = 3 independent experiments are shown. **b** Quantitation of p-ERK inhibition; normalized values (mean ± SEM, *n* = 3 independent experiments) of p-ERK levels obtained by densitometry with corresponding fitted curves (see “Methods” section). **c** SKMEL239-C4 cells without Encorafenib (−Enco) treatment and after Encorafenib (+Enco) treatment for 1 h, followed by exchange with fresh medium for another hour, were treated with increasing concentrations of **c** PHI1, **e** LY3009120, **g** AZ-628 and **i** TAK-632 for 1 h and cell lysates were assayed by western blot with the indicated antibodies to assess ERK-pathway inhibition. A representative blot from *n* = 3 (**c***,* **d**) and *n* = 2 (**e**–**j**) independent experiments is shown. Normalized values and non-linear regression fits of p-ERK activity for different compounds in **c**, **e**, **g**, and **i** is shown respectively. Error bars represent mean ± SEM, *n* = 3 (**d**) and mean of two replicates from *n* = 2 independent experiments (**f**, **h**, **j**). **k** Table summarizing p-ERK inhibition results from all inhibitors in Encorafenib-free and Encorafenib-treated SKMEL239-C4 cells. Source data are provided as a Source Data file.

Guided by the distinct binding properties and structural effects of PHI1 compared to Ponatinib, we investigated the cellular activity of PHI1. We assessed inhibition activity on BRAF and ERK signaling in melanoma cells expressing BRAF^V600E^ monomers (A375 cells) and constitutively expressed p61BRAF^V600E^ dimers (SKMEL239-C4 cells). The inhibitory activity of PHI1 in A375 cells (IC_50_ = 2760 nM) is significantly reduced compared to Ponatinib (IC_50_ = 291 nM), whereas in SKMEL239-C4 (IC_50_ = 424 nM) it is assessed at similar levels compared to Ponatinib (IC_50_ = 452 nM) (Fig. 6a, b). Similar selectivity profiles for Ponatinib (IC_50_ = 569 nM) and PHI1 (IC_50_ = 2045 nM) were also observed in SKMEL239 parental cells expressing BRAF^V600E^ monomers (Supplementary Fig. 16). Consistently, in HEK293 cells ectopically expressing p61BRAF^V600E^ dimers versus p61BRAF^V600E^ harboring the monomer-driver R509H mutation, PHI1 showed preferential inhibition for p61BRAF^V600E^ compared to p61BRAF^V600E/R509H^ BRAF species (Supplementary Fig. 17). Taken together, these results demonstrate that PHI1 more effectively targets p61BRAF^V600E^ dimers than BRAF^V600E^ monomers.

These data suggest that the interactions of the morpholine-based head group of PHI1 with the αC-allosteric site controls the specificity for the BRAF dimer. To confirm this, we investigated the activity of PHI2, a compound with an altered head group, but with the same PHI1 and Ponatinib scaffold (Supplementary Fig. 18a). We selected to maintain this common, type-II scaffold, to ensure that the αC-IN binding mode to BRAF is maintained. PHI2 potently inhibited BRAF kinase activity in vitro (Supplementary Fig. 18b). However, the inhibitory activity of PHI2 for p61BRAF^V600E^ dimers (SKMEL239-C4 cells) is reduced, while it is increased for BRAF^V600E^ monomers (A375 cells), in comparison to the activity of PHI1 (Supplementary Fig. 18c). Collectively, our data suggest that recognition of the αC-allosteric site by BRAF inhibitors can result in distinct specificity for BRAF^V600E^ dimers.

Since PHI1 is a weak inhibitor of BRAF^V600E^ monomers but inhibits BRAF^V600E^ dimers more effectively, we reasoned that it may be more potent for the second site than the first site within the BRAF dimer. To assess this, we used a previously established assay and treated SKMEL239-C4 cells with 1 μΜ Encorafenib, a potent inhibitor of BRAF^V600E^ monomers that remains bound to the first site within the dimer up to 24 h after a wash-out treatment, due to its low *K*_off_^11,45^. Indeed, in control experiments, we observed that Encorafenib treatment for 1 h inhibited p-ERK, but the activation is recovered after wash-out for another hour due to half-occupied active dimers (Supplementary Fig. 19). Following this approach, we determined the inhibitory activity for the second site of the BRAF^V600E^ dimer for various BRAF inhibitors (Fig. 6c–k).

Remarkably, PHI1 inhibited more potently the second site within the BRAF^V600E^ dimer (IC_50_ = 134 nM) than the Encorafenib-untreated BRAF^V600E^ dimer (IC_50_ = 424 nM) or the BRAF^V600E^ monomer (IC_50_ = 2760 nM) (Fig. 6c, d). This enhanced potency for the second site of the BRAF^V600E^ dimer indicates positive co-operativity of binding to the second site induced by occupancy of the first site. In contrast, Vemurafenib showed very weak inhibition of both the second site within the BRAF^V600E^ dimer (IC_50_ = 2504 nM) and the Encorafenib-untreated BRAF^V600E^ dimer (IC_50_ = 2425 nM) (Supplementary Fig. 20), as expected by its negative allosteric effect. On the other hand, Ponatinib had similar inhibition activity (IC_50_ = 495 nM) for the second site compared to Encorafenib-untreated BRAF^V600E^ dimer (IC_50_ = 452 nM) (Supplementary Fig. 20). We excluded the possibility that positive co-opertivity arises from elimination of cellular activity from residual BRAF^V600E^ monomers by Encorafenib pre-treatment, as monomeric species are not observed upon size-exclusion chromatography analysis of SKMEL239-C4 cell extracts (Supplementary Fig. 21). To confirm whether positive co-operativity by PHI1 is indeed unique property among BRAF αC-IN/type-II inhibitors, we compared second-site binding profiles of three inhibitors that inhibit BRAF monomers and dimers^11,45^. LY3009120 (Fig. 6e, f), AZ-628 (Fig. 6g, h), and TAK-632 (Fig. 6i, j) demonstrated very similar potency in inhibiting the second site within the BRAF^V600E^ dimer or the Encorafenib-untreated BRAF^V600E^ dimer, suggesting equipotent binding of the first and second-site within the dimer by these inhibitors. In contrast, PHI1 exerts more potent inhibition of the second site of the BRAF^V600E^ dimer (Fig. 6k). To examine whether PHI’s selectivity is linked to direct target engagement for BRAF^V600E^ dimers, we performed Cellular Thermal Shift Assay (CETSA) in A375 and SKMEL239-C4 cells, under inhibitor-saturated conditions. CETSA showed that Ponatinib stabilizes BRAF^V600E^ monomers in cells (Δ*T*_m_ = +6.3 °C) more effectively compared to PHI1 (Δ*T*_m_ = +3.4 °C) (Supplementary Fig. 22a, b). In contrast, PHI1 and Ponatinib display similar stabilization effect with p61BRAF^V600E^ dimers (Δ*T*_m_ = +3.2 °C) (Supplementary Fig. 22c, d). Although *T*_m_ values may not reflect true binding affinity ranking under these conditions, these results are in agreement with the observed inhibition selectivity of PHI1 and Ponatinib (Fig. 6a, b).

**Fig. 7:**
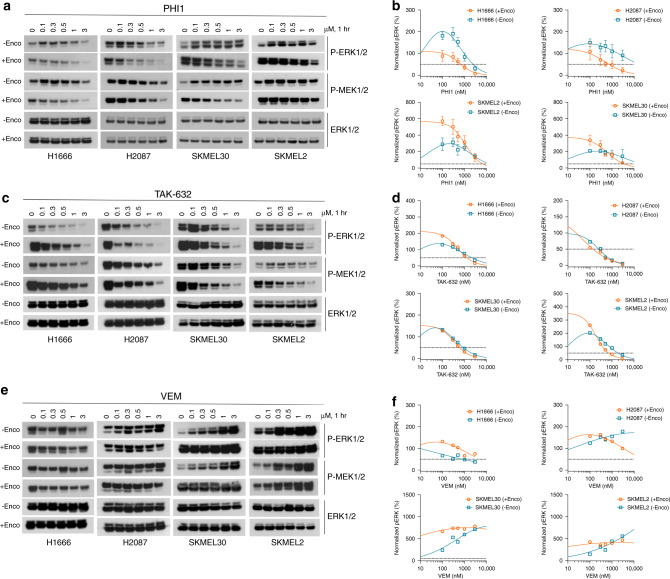
Positive co-operativity in inhibition of non-BRAFV600E dimers by PHI1. Lung cancer H1666 (*BRAF*^*G*466V^), H2087 (BRAF^L597V^), and melanoma SKMEL-30 (BRAF^WT^/NRAS^Q61R^), SKMEL-2 (BRAF^WT^/NRAS^Q61K^) cells without Encorafenib (−Enco) treatment and after Encorafenib (+Enco) treatment for 1 h, followed by exchange with fresh medium for another hour, were treated with increasing concentrations of **a** PHI1, **c** TAK-632, and **d** Vemurafenib for 1 h and cell lysates were assayed by western blot with the indicated antibodies to assess the ERK-pathway inhibition. A representative blot from *n* = 3 (**a**, **b**) and *n* = 2 (**c**, **e**) independent experiments is shown. Normalized values and non-linear regression fits of p-ERK activity for each treatment is shown in **b**, **d**, and **f**, respectively. Error bars represent mean ± SEM, *n* = 3 (**b**) and mean of two replicates from *n* = 2 independent experiments (**d**, **f**). Notably, Encorafenib pre-treatment promotes paradoxical activation in these cells. Source data are provided as a Source Data file.

**Fig. 8:**
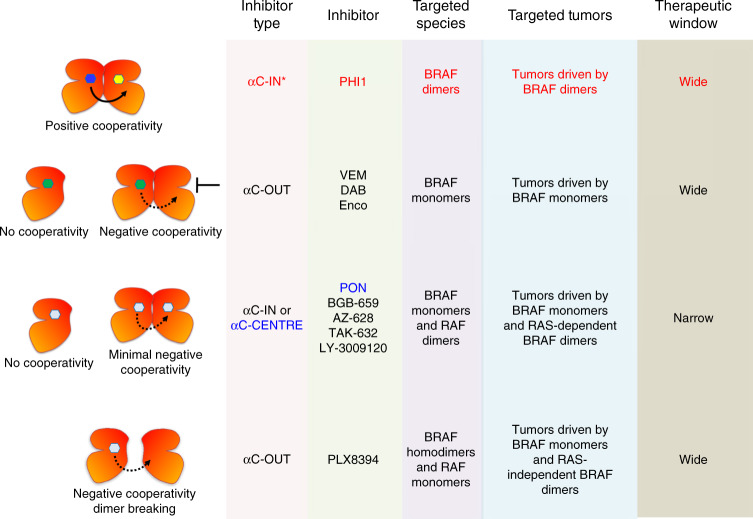
Mechanisms of RAF inhibitors action. Scheme of four distinct mechanisms of RAF inhibitors action and representative RAF inhibitors for each mechanism. This work revealed that PHI1 displays a distinct structural, inhibitory, and therapeutic mode of action compared to previously characterized αC-IN and αC-OUT RAF inhibitors. Moreover, Ponatinib, an FDA-approved inhibitor, is an effective inhibitor of BRAF monomers and RAF dimers with a distinct structural binding mode.

Finally, we asked whether second-site positive co-operativity by PHI1 extents to RAS-dependent (class-II) or RAS-independent (class-III) non-V600E-mutated BRAF dimers and RAS^MUT^/BRAF^WT^ dimers^46^. To answer this, we assessed PHI1’s inhibitory effect in lung adenocarcinoma H2087 (BRAF^L597V^, class-II) and H1666 (BRAF^G466V^, class-III) cells, and SKMEL-2 and SKMEL-30 melanoma cell lines driven by RAS^MUT^/BRAF^WT^. In Encorafenib-untreated cells, we detected low p-ERK inhibition and various degrees of paradoxical activation by PHI1, which was pronounced in H1666 and BRAF^WT^ melanoma cells (Fig. 7a, b). In contrast, after Encorafenib pre-treatment, PHI1 exhibited sub-micromolar p-ERK inhibition in both H2087 (IC_50_ = 485 nM) and H166 (IC_50_ = 590 nM) lung cancer cell lines, with more prominent effect in H2087, suggesting a strong positive co-operativity of PHI1 within non-V600E-mutated BRAF dimers present in these cells^46^ (Fig. 7a, b). However, PHI1 required low micromolar dose to inhibit signaling in RAS^MUT^/BRAF^WT^ without evidence of positive co-operativity upon Encorefenib pre-treatment (Fig. 7a, b). Furthermore, as with BRAF^V600E^ dimers (Fig. 6i, j), αC-IN/type-II inhibitor TAK-632 demonstrated equipotent inhibition of BRAF dimer species and no positive co-operativity in these cell lines, while αC-OUT/type-I inhibitor Vemurafenib was largely inactive, without and with Encorefenib pre-treatment (Fig. 7e, f). Taken together, these results suggest that PHI1 has a unique specificity for the second-site of BRAF dimers and a distinct inhibition mechanism of positive co-operativity among different types of BRAF inhibitors (Fig. 8).

## Discussion

BRAF mutational analyses in human cancer and complex biochemical mechanism of RAF activation intensified efforts for the development of selective and potent BRAF inhibitors^8,9,13,21^. Clinical BRAF inhibitors effectively target BRAF^V600E^ monomers, but it is well established that such inhibitors do not inhibit oncogenic BRAF dimers and promote paradoxical activation in cells with wild-type BRAF^11–14^. Inhibitors that potently inhibit BRAF dimers may have a broader efficacy in several BRAF-dependent tumors and in combination with clinical BRAF inhibitors may also overcome adaptive and acquired resistance^12,13,15,47^. Recent drug development efforts have yielded αC-IN RAF inhibitors that equipotently inhibit BRAF monomers and dimers without selectivity^11–14^. In addition, a recent report identified an αC-OUT RAF inhibitor, PLX8394, which selectively disrupts BRAF dimers in RAS-independent BRAF-mutant-driven signaling^45^.

Here, we identified Ponatinb, an FDA-approved kinase inhibitor, as a hitherto uncharacterized potent BRAF inhibitor, which binds either BRAF^V600E^ monomers or BRAF dimers. With either BRAF variant, Ponatinib promotes formation of inhibited BRAF/CRAF dimers and BRAF/MEK1/2 complexes. In various cellular contexts, the net inhibitory effects of Ponatinib will depend on the combined effect of its targeting for BRAF monomers and dimers and its simultaneous capacity to form new dimers^12,13^. Therefore, it is not surprising that Ponatinib is more effective in cells with activated BRAF^V600E^ and low active RAS, as opposed to cells with active RAS^MUT^BRAF^WT^ that can induce new RAF dimers due to RAF priming. Nevertheless, as it is suggested by the Ponatinib-induced BRAF dimer structure, Ponatinib has diminished negative allostery within dimers and inhibits each protomer (first site and second site) with the same potency. Recently developed αC-IN RAF inhibitors^11–14,45^ (e.g., LY3009120), which have equipotent inhibition for BRAF monomers and dimers with similar efficacy to Ponatinib and promote new RAF dimers due to inhibitor-induced RAF priming, have entered clinical trials in oncogenic BRAF-dependent tumors such as melanoma, colorectal, and other advanced solid tumors. Therefore, our findings may have clinical implications and enable repurposing of Ponatinib in melanoma and other oncogenic BRAF-dependent tumors, more likely in a combination therapy with a MEK inhibitor or immunotherapy. The maximum plasma levels (~100 nM) and prolonged elimination half-life (>24 h) of Ponatinib in standard of care dose for CML suggest that Ponatinib would be clinically effective for targeting BRAF and ERK signaling inhibition^48^. Furthermore, potential dual targeting of BRAF and RTK inhibition by Ponatinib in a specific oncogenic context (e.g., BRAF^V600E^ and FGFR aberration) or in the context of adaptive resistance mechanisms through RTK signaling may be particularly effective to suppress ERK signaling.

Our structural findings based on co-crystal structures of Ponatinib and PHI1 highlight that each inhibitor recognizes a distinct allosteric site and induces distinct dimers and αC-helix conformation. While Ponatinib promotes dimers with αC-CENTRE conformation, PHI1 induces an αC-IN* conformation within the BRAF^V600E^ dimer through interactions within its allosteric site. Indeed, we found that these interactions result in gain of specificity for PHI1 compared to Ponatinib for other kinase targets using the KinomeEDGE screen. Although this in vitro screen shows that a common subset of RTKs binds both Ponatinib and PHI1 (Supplementary Table S2), the distinct cellular BRAF monomer-dimer selectivity by the compounds is consistent with the effects of these compounds being RTK-independent. Remarkably, the αC-allosteric site (BP-VI) revealed by PHI1 binding is not predicted in the current available kinase structures^43^. Additional drug design around this site should allow further fine-tuning of selectivity and potency for BRAF inhibition.

Our data demonstrate that PHI1 has a very weak inhibition for the BRAF^V600E^ monomers compared to the BRAF^V600E^ dimers, suggesting that interactions with the αC-allosteric site (BP-VI) are favored in the dimer conformation while are unfavorable in the monomer conformation. Our data using the PHI2 compound showed that selectivity can be modulated by the head group attached to the Ponatinib and PHI1 core scaffold. Crystallization of PHI2 with BRAF or PHI1 with BRAF monomer was not successful, probably due to enhanced destabilization of αC-helix conformation. It is noteworthy that PHI1’s distinct cellular selectivity compared to Ponatinib is not recapitulated by in vitro kinase inhibition systems (Supplementary Figs. 5 and 13), apparently due to absence of full structural and functional BRAF regulation^13,14^. We found that PHI1 inhibits more potently the second site of p61BRAF^V600E^ dimers when the first site within the dimer is occupied, uncovering the first paradigm of positive co-operativity for RAF inhibitors. This suggests that the allosteric coupling of PHI1 with αC-helix via specific interactions, promotes this positively co-operative effect, while Ponatinib, LY3009120, AZ-628, and TAK-632 target both sites with similar potency, as do other αC-IN/type-II BRAF inhibitors^45^.

PHI1 also exhibited a co-operative mode of inhibition in oncogenic non-V600E BRAF dimers belonging to class-II and class-III, but not in RAS^MUT^BRAF^WT^ driven melanomas. Although structural details on non-V600E mutants are elusive, these results demonstrate that both class-II and class-III oncogenic BRAF dimers species can be subjected to allosteric coupling by PHI1, driven by occupancy of the first protomer. Since signaling by class-III dimers depends mainly on heterodimerization with BRAF^WT^ or CRAF protomers^46^ these data indicate that activated RAF heterodimers are susceptible to this inhibitory mechanism. It is unclear why RAS-activated BRAF^WT^ dimers are refractory to positive co-operativity by PHI1, but this may be related to enhanced priming and low occupancy of the first protomer.

In summary, RAF inhibitors can modulate BRAF inhibition by four distinct mechanisms (Fig. 8): (i) negative co-operativity by αC-OUT inhibitors that inhibit BRAF^V600E^ monomers (ii) no co-operativity by αC-IN or αC-CENTRE (Ponatinib) inhibitors that equipotently inhibit BRAF monomers and dimers (iii) dimer breaking activity such as αC-OUT PLX8394 inhibitor and a new category (iv) positive co-operativity by αC-IN* inhibitors exemplified by PHI1 that selectively inhibit BRAF^V600E^ and other oncogenic BRAF dimers. Inhibitors such as PHI1, may be particularly effective in blocking BRAF and ERK signaling in tumors expressing different BRAF dimer species of various classes, BRAF fusions or deletions^45–50^, when used alone or in combination with αC-OUT inhibitors that effectively inhibit the first site of the BRAF dimers.

To conclude, our strategy for identifying BRAF dimer kinase inhibitors demonstrates the significant value of screening specific phenotypic assays with additional kinase inhibitors to identify alternative uses and targets. There are several examples of kinase inhibitors that were developed for a specific kinase and indication, and later were found to have additional targets and effectiveness in a different cellular context or disease^51–53^. We foresee that a similar approach will be increasingly used to yield repurposing opportunities as well as chemical biology applications and drug development campaigns. Collectively, our data provide unexpected findings and useful mechanistic insights for BRAF targeting and hold promise for the development of next-generation inhibitors for the treatment of BRAF-dependent tumors.

## Methods

### Compounds

Kinase inhibitors library was obtained from Selleck. Vemurafenib, Encorafenib, Trametinib, TAK-632, AZ-628, LY3009120, Lapatinib, and Ponatinib were purchased from Selleck and purity >99% was confirmed by NMR and MS. PHI1 and PHI2 compounds were synthesized in >99% purity and confirmed by NMR and MS. All compounds were dissolved in DMSO to a 10 mM stock solution.

### Antibodies

BRAF (1:1000, Santa Cruz sc-5284), BRAF^V600E^ (1:1000, NewEast BioSciences 26039), CRAF (1:1000, Santa Cruz C-12), MEK1 (1:1000, Millipore Millipore, 07-641), MEK1/2 (1:1000, Cell Signaling 4694), P-MEK1/2 (1:1000, Cell Signaling 9154), ERK1/2 (1:1000, Cell Signaling 4696), P-ERK (1:200, Santa Cruz sc-7383), P-ERK1/2 (1:500, Cell Signaling 4370), ERK1 (1:200, Santa Cruz sc-94), Actin (1:10000, Invitrogen MA5-15739), GAPDH (1:5000, Sigma, G8795), IRDye800CW anti-mouse (1:800, LICOR 926-32210), and IRDye680RD anti-rabbit (1:800, LICOR 926-68071).

### Cell culture

Cell lines were purchased from ATCC or provided by Poulikos Poulikakos laboratory. All cell lines were tested negative for mycoplasma contamination and authenticated by morphology and STR profiling. A375, SKMEL239, SKMEL-30, and SKMEL-2 cells were grown in Dulbecco’s modified Eagle’s medium (DMEM) with 10% fetal bovine serum (FBS), 1% Pen-Strep, and 1% Glutamine. SKMEL239-C4 cells were grown in Dulbecco’s modified Eagle’s medium (DMEM) with 10% fetal bovine serum (FBS), 1% Pen-Strep, and 1% Glutamine in the presence of 1 μM Vemurafenib. CALU6 cells were grown in Roswell Park Memorial Institute (RPMI) 1640 medium with 10% FBS, 1% Pen-Strep, and 1% Glutamine. H1666 and H2087 cells were grown in RPMI 1640 medium with 5% FBS, 1% Pen-Strep, and 1% Glutamine.

### In-cell-western screening

SKMEL239-C4 melanoma cells were plated in 96-well plate in DMEM 10% FBS, 1% Pen-Strep, and 1% Glutamine, and allowed to seed overnight. Media was removed and replaced with fresh media containing 0.5 μM Vemurafenib and treated with 5 μM of corresponding kinase inhibitors and incubated for 3 h. Cells were then fixed in 4% formaldehyde for 20 min at room temperature (RT) and washed four times with 0.1% Triton in 1× PBS for 5 min at RT with gentle rocking. Cells were then rinsed with 1× PBS and stored in 1× PBS at 4 °C for future in-cell-western (ICW). For ICW, we followed LiCor PI-140 0103 Doc #988-07083 protocol with some modifications (https://www.licor.com/documents/k12xs979o8ku50313v3r0n15n47w0f0c). In brief, cells were blocked with Odyssey blocking solution (LiCor) for 1 h at RT. Then cells were incubated with primary antibodies diluted in odyssey blocking buffer (1:200 P-ERK and 1:200 ERK1) for 2 h at RT, followed by four washes with 0.1% Tween-20 in 1× PBS (PBST) for 5 min at RT. Then cells were incubated for 2 h at RT with secondary antibodies diluted in odyssey blocking buffer containing 0.2% Tween-20 (1:800 IRDye800CW anti-mouse and 1:800 IRDye680RD anti-rabbit) and washed 4 times with PBST for 5 min at RT. Cell were then rinsed once with 1× PBS, aspirated off and the plate was scanned with detection in both 700 and 800 nm channel using Odyssey Classic imager (ODY-0671). Quantification and analysis was performed using the Western Analysis tool from the Image Studio 3.1 software. Percent of phosphorylated-ERK1 was calculated as the total fluorescence levels of phosphorylated-ERK1 antibody staining divided by the total fluorescence levels of ERK1 antibody staining and normalized to percent of phosphorylated-ERK1 of untreated cells.

### Analysis of ERK-signaling

Melanoma cells (A375, SKMEL239-C4, SKMEL-30, SKMEL-2) were plated in 6-well plates in DMEM with 10% FBS, 1% Pen-Strep, and 1% Glutamine to 70–80% confluency and lung cancer cells (H1666, H2087) to 90% confluency in RPMI 1640 medium with 5% FBS, 1% Pen-Strep, and 1% Glutamine. SKBR3 cell were grown to 70% confluency in McCoy’s 5 A Medium with 10% FBS, 1% Pen-Strep and 1% Glutamine and 2200 mg/L sodium bicarbonate. To assess ERK-signaling, cells were treated with DMSO or compounds for 1 h and analyzed by western blotting. For ^eE^ncorafenib wash-out experiments, cells in 6-well plates were pre-treated with 1 μΜ Encorafenib for 1 h, washed 3× with PBS and incubated for 1 h with fresh medium, followed by DMSO or compound treatment. For evaluation of RTK-dependence of inhibition by Ponatinib, before Ponatinib treatment cells were pre-treated with 1 μM Lapatinib for 1 h and washed 3× with PBS. Western blots were performed from whole-cell lysates (WCL) prepared in lysis buffer containing 50 mM Tris-HCl pH 7.5, 1% NP40, 150 mM NaCl, 1 mM EDTA, and 10% glycerol in the presence of protease inhibitor cocktail (Roche). WCL were separated on 4–12% NuPAGE MES gel (Invitrogen), transferred on a PVDF membrane, blocked for 1 h, and immunoblotted with the corresponding antibodies. To increase data output, the multistrip western blotting method was used for a selection of experiments^54^.

### Co-immunoprecipitation and kinase activity assays

Co-immunoprecipitation assays were performed from whole-cell lysate prepared in lysis buffer in the presence of protease inhibitor cocktail (Roche) and incubated at 4 °C overnight with gentle rotation, then protein-G beads were added and incubated for another 2 h at 4 °C. Kinase activity assays were performed using BRAF Kinase Assay Kit (#17-359), CRAF Kinase Assay Kit (#17-360) and MAP Kinase/Erk Assay Kit (#17-191), following protocols by the provider (Millipore) with some modifications. In brief, BRAF and CRAF, and MEK1 recombinant proteins were incubated for 15 min with inhibitors, then corresponding substrates were added and incubated for 30 min at 30 °C and assayed via western blot.

### Size-exclusion chromatography of SKMEL239-C4 cell extracts

SKMEL239-C4 melanoma cells were plated in 6-well plates in DMEM 10% FBS, 1% Pen-Strep, and 1% Glutamine to 60% confluency. Cells were incubated in the absence of Vemurafenib (DMSO) or with 2 μM Vemurafinib for 72 h. After incubation cells were washed with PBS, collected and lysed in 50 mM Tris-HCl pH 7.5, 1% NP40, 150 mM NaCl, 1 mM EDTA plus protease inhibitors. Freshly lysed whole-cell lysates (2.5 mg total protein) were loaded onto a Superdex200 HR 10/300 (GE Healthcare) column and run at a flow rate of 0.2 mL/min. Fractions (0.5 ml) were collected and western blotted for BRAF^V600E^.

### p61BRAF^V600E^ expression in HEK293H cells and PON treatment

HEK239H cells were seeded in 6-well plates (1 × 10^6^ cells per well) and transfected the following day using Lipofectamine 3000 (Invitrogen L3000008) according to the manufacturer’s instructions. 2 μg of p61BRAF^V600E^ plasmid (pcDNA-3 vector) DNA per well was employed. 24 h after transfection cells were treated with DMSO or increasing concentrations of Ponatinib for 1 h. ERK signaling and BRAF^V600E^ expression was assessed by western blot. Untransfected cells treated with DMSO or 1 μM Ponatinib were used as negative control. In addition, HEK239H cells seeded in 6-well plates (1 × 10^6^ cells per well) were transfected with Lipofectamine 3000 containing 2 μg of p61BRAF^V600E^ plasmid or 2 μg of p61BRAF^V600ER509H^ plasmid (pcDNA-3 vector) DNA per well. 24 h after transfection cells were treated with DMSO or increasing concentrations of PHI1 for 1 h and ERK signaling, and BRAF expression was assessed by western blot. Cells treated with DMSO or 10 μM Vemurefenib were used as positive control for inhibition of p61BRAF^V600E/R509H^ and no inhibition of p61BRAF^V600E^ signaling.

### Densitometric analysis and quantification

Densitometric data for P-ERK, P-MEK, ERK1/2 (MAPK cellular activity), or BRAF and GAPDH (CETSA analysis) from western blot scanned films were obtained using Image Studio software (LI-COR). Data were corrected to loading control (total ERK1/2 or GAPDH) and normalized to DMSO-treated bands (100%) and blot backgrounds (0%). IC_50_ or *T*_m_ values were obtained from non-linear regression fits of normalized data to a four-parameter logistic curve (4PL), using GraphPad Prism 8.

### 3D culture of melanoma cells

3D laminin-rich extracellular matrix cultures were prepared by trypsinization of melanoma cells from tissue culture plastic, seeding of single cells on top of a thin layer of Growth Factor-Reduced Matrigel (BD Biosciences, Franklin Lakes, NJ), and the addition of a medium containing 5% Matrigel. The cells were seeded at the density of 40,000 cells per cm^2^. All 3D cell cultures were performed in DMEM supplemented with 5 μg/mL bovine insulin with Zinc (Gibco), 15 μg/mL bovine pituitary extract (Gibco), 0.5 ng/mL epidermal growth factor (Sigma-Aldrich, St. Louis, MO) and 2% fetal bovine serum. 3D cultures were maintained with media and/or drug-treatment changes every second day, and photographs were taken at the end of the 5th day. The size of single cell colonies was estimated with ImageJ (Version 1.51).

### Cellular thermal shift assay (CETSA) analysis

For CETSA analysis, cultured A375 or SKMEL239-C4 cells were washed with Dulbecco’s phosphate buffered saline (DPBS) and split into 500 μL aliquots (each containing 375 million cells) in the same buffer, containing 20 μΜ (A375 cells) or 70 μM (SKMEL239-C4 cells) of PON, PHI1 or DMSO. The samples were incubated for 1 h at room temperature, rotating. After compound incubation, samples (50 μL each) were transferred in PCR tubes and incubated for 3 min in a temperature gradient produced with a C1000 thermal cycler (Bio-Rad). Cells were immediately lysed by repeating freeze-thaw cycles (3× times) in liquid nitrogen. Lysates were spun in a microcentrifuge at 15,000×*g* for 15 min at 4 °C. Equal volumes of supernatants were run on 15-well 4–12% NuPAGE SDS-PAGE gels (Invitrogen), and analyzed by western blot. GAPDH, which is temperature insensitive under these conditions, served as loading control. Results were quantitated by densitometric analysis as described above.

### Cloning, expression, and purification of BRAF

Human BRAF kinase domain (residues 443–723) with V600E mutation in addition to designed mutations to improve expression in *E. coli*^12^ was cloned into the first multiple cloning site of a pET-28a vector, which expresses a hexa-histidine tag at the N terminus of BRAF. Recombinant protein was transformed and expressed into *E. coli* strain BL21-Codon Plus (DE3)-RIPL (Agilent Technologies). Protein purification was performed by a rapid two-step procedure using nickel-affinity chromatography (Ni-NTA) followed by size-exclusion chromatography with Superdex200 HR 10/300 (GE Healthcare). Ponatinib or PHI1 at 1.5 molar excess to the protein sample were added immediately after elusion from Ni-NTA column.

### Crystallization

Purified complexes of BRAF^V600E^ kinase domain with Ponatinib or PHI1 were concentrated to 4.5–5.5 mg/mL using a filtration unit (Millipore). Initial crystallization hits of the complexes were obtained in the HT Crystal HT screen (Hampton Research) using the sitting-drop vapor-diffusion method and 96-well Intelli-plates (Hampton Research) at 293 K. Optimized crystals of BRAF^V600E^-Ponatinib were generated in 2 M ammonium sulfate and 5% (v/v) 2-propanol and of BRAF^V600E^-PHI1 in 1 M sodium citrate. Crystals were cryo-protected shortly in mother liquor supplemented with 20 % (v/v) ethylene glycol and flash-frozen in liquid nitrogen.

### Structure determination

Diffraction data to 2.1 Å resolution were collected using microbeam beamline 23-ID-D at Advanced Photon Source (APS) of Argonne National Laboratory. X-ray data were processed with the program iMosflm (Version 7.2.1)^55^. The BRAF^V600E^ structure bound to Ponatinib or PHI1 was solved by the molecular replacement method in Phaser of CCP4 suite (Version 7.0)^56^ using the BRAF/AZ-628 structure (PDB 4RZW) as search model. Structure refinement was performed using REFMAC (Version 5.8.0158)^57^. Statistics of the diffraction data and refinement are summarized in Supplementary Table S1. Coordinates have been deposited with the Protein Data Bank (BRAF^V600E^–PON complex: PDB 6P3D, BRAF^V600E^–PHI1 complex: PDB 6P7G).

### Structural analysis

Software was available through the SBGrid collaborative network^58^. Structural analysis and 3D-superpositions were performed in Pymol (Version 2.3, The PyMOL Molecular Graphics System, Schrödinger, LLC) and Maestro tools (Maestro, Schrödinger Release 2018-3, Schrödinger, LLC). Interface interaction analysis between ligands and BRAF^V600E^ was performed using PISA in CCP4 (Version 7.0).

### Recombinant kinase activity assay

BRAF kinase assays were performed using the Z’-LYTE™ enzymatic assay (Invitrogen, USA). Briefly, kinase activity was monitored in a cascade system consisting a mixture of inhibitor with BRAF or BRAF^V600E^/inactive MAP2K1 (MEK1)/inactive MAPK1 (ERK2)/Ser/Thr peptide (Invitrogen) in 50 mM HEPES pH 7.5, 100 μM ATP, 10 mM MgCl_2_, 1 mM EGTA, and 0.01% Brij-35. Titrations were performed using a 1:3 dilution. Assays were performed using SelectScreen (Invitrogen).

### Kinome specificity

The KINOMEscan^®^ screening platform (Eurofins/DiscoverX) was used to assess binding specificity of Ponatinib and PHI1 at 1 μM across a panel of diverse kinases (scanEDGE panel). Maps were generated using TREEspot software (Version 5.0, http://www.kinomescan.com) and display a circular representation of the kinase family tree based on kinase domain sequence. Selectivity score, a quantitative measure of compound selectivity, was calculated using the formula *S*-score(10) = (number of non-mutant kinases with %Ctrl <10%)/(number of non-mutant kinases tested), where %Ctrl represents the percent of positive control compound that remain bound to each kinase in the presence of the test compound.

### Pharmacophore-based drug design

An in silico library of 3D compounds based on eMolecules (www.emolecules.com) library of 6.5 million purchasable compounds was generated using LIGPREP (Schrödinger Release 2017-4, Schrödinger, LLC) and EPIK (Schrödinger Release 2017-4, Schrödinger, LLC). The in silico library contained approximately 13.8 million compounds with different ionization state at pH 7.0 ± 2.0, stereochemistry and tautomeric form, excluding potential Pan Assay Interference Compounds (PAINS) using PAINS definitions included in Canvas (Schrödinger Release 2017-4, Schrödinger, LLC). Conformation analysis of ligands was calculated using the OPLS3 force field. Phase (Schrödinger Release 2018-3, Schrödinger, LLC) module was used to generate a pharmacophore hypothesis and a 3D pharmacophore screen^59^. The coordinates of the BRAF/Ponatinib structure were used and a pharmacophore hypothesis was generated to preserve trifluoro-phenyl and piperazine interactions of Ponatinib with BRAF and further additional interactions within the allosteric pocket. Pharmacophore hypothesis included six features as defined in Phase and included one aromatic ring, two positively charged groups, two hydrophobic points and one hydrogen bond acceptor. The pharmacophore screen searched the in silico library with the requirement to satisfy at least five out of the six pharmacophore features of the hypothesis. Two hundred hits were obtained that satisfied at least five out of seven pharamacophore constrains, comprising of diverse chemical groups. Fragments were computationally linked to the Ponatinib core structure (replacing the piperazine moiety) to produce Ponatinib hybrid inhibitors. Virtual compounds were prepared with LIGPREP and EPIK and docked to BRAF^V600E^ using GLIDE (Schrödinger Release 2018-3, Schrödinger, LLC). Docked compounds that demonstrated excellent fit to the pharmacophore model and have the most favorable interaction energies were selected for synthesis. Physicochemical and AMDET properties including Lipinski rules, permeability, logP, metabolic liabilities, and hERG inhibition were evaluated using QikProp (Schrödinger Release 2018-3, Schrödinger, LLC) to maintain or improve Ponatinib properties.

### Statistical analysis

The number of independent experiments for each data set is stipulated in the respective figure legend. Statistical significance for pair-wise comparison of groups was determined by two-tailed Student’s *t*-test and by one-way ANOVA using GraphPad PRISM software (Version 7.0, GraphPad Inc., CA).

### Reporting summary

Further information on research design is available in the Nature Research Reporting Summary linked to this article.

## Supplementary information

Supplementary Information

Reporting Summary

Peer Review

## Data Availability

Data generated or analyzed during the study are available from the corresponding authors upon reasonable request. BRAF^V600E^/Ponatinib and BRAF^V600E^/PHI1 structure coordinates have been deposited in the PDB with accession codes 6P3D and 6P7G, respectively. Source data are provided with this paper.

## Source data

Source Data
