# Supplementary material for: Inhibitors of BRAF dimers using an allosteric site
Source: Nat Commun. 2020 Sep 1;11:4370. doi: 10.1038/s41467-020-18123-2 (PMC7462985; doi:10.1038/s41467-020-18123-2)
Supplement: Supplementary file 1 — Supplementary Information [file 41467_2020_18123_MOESM1_ESM.pdf]

Supplementary Information

Inhibitors of BRAF dimers using an allosteric site

Cotto-Rios et al.

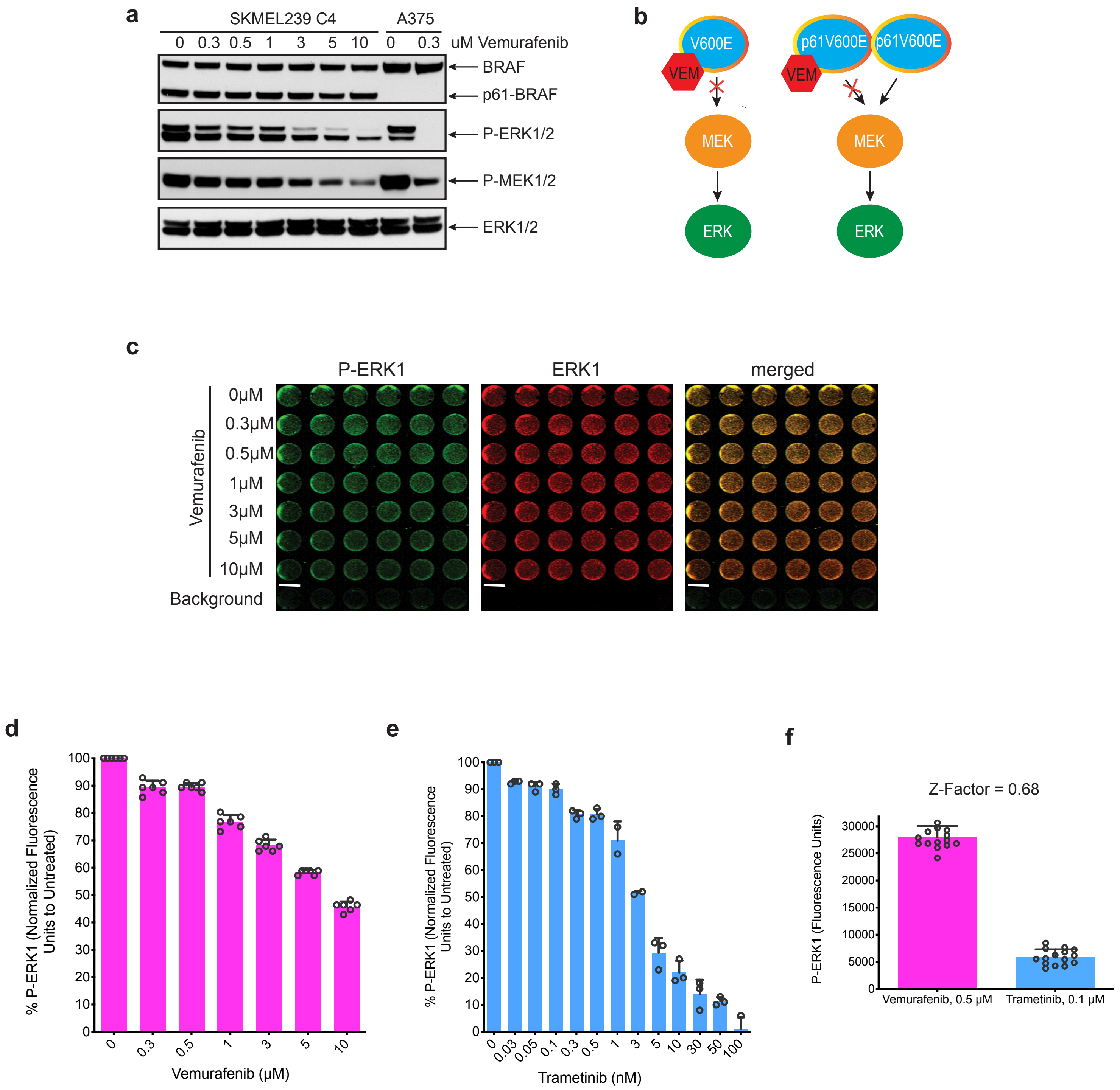
a
SKMEL239 C4
A375
0 0.3 0.5 1 3 5 10 0 0.3 uM Vemurafenib
BRAF
p61-BRAF
P-ERK1/2
P-MEK1/2
ERK1/2
b
V600E
VEM
MEK
ERK
p61V600E
p61V600E
VEM
MEK
ERK
c
P-ERK1
ERK1
merged
Vemurafenib
0μM
0.3μM
0.5μM
1μM
3μM
5μM
10μM
Background
d
% P-ERK1 (Normalized Fluorescence Units to Untreated)
Vemurafenib (μM)
e
% P-ERK1 (Normalized Fluorescence Units to Untreated)
Trametinib (nM)
f
Z-Factor = 0.68
P-ERK1 (Fluorescence Units)
Vemurafenib, 0.5 μM
Trametinib, 0.1 μM

Supplementary Figure 1. In-cell-western screen assay for inhibition of phosphorylation of ERK.

(**a**) SKMEL239 C4 and A375 melanoma cells were treated with increasing concentrations of Vemurafenib for 3 hrs and cell lysates were immunoblotted with the indicated antibodies. Blot is representative of n=3 independent experiments (**b**) Schematic illustrates the inability of Vemurafenib to inhibit the dimer of p61BRAF^{V600E} in SKMEL239 C4 cells compared to its capacity to inhibit the monomer BRAF^{V600E} in A375 cells. (**c**) SKMEL239 C4 cells left untreated or treated with increasing concentrations of Vemurafenib for 3 hrs and then assayed for in-cell-western. In-cell-western images of the assay plate showing staining of cells with phosphorylated-ERK1 (green), ERK1 (red) antibodies and their merged image. In-cell western is representative of n=3 independent experiments. Scale bars, 0.64 cm. (**d***)* Quantification and analysis of images in (c). Percent of phosphorylated-ERK1 calculated by taking the total fluorescence levels of phosphorylated-ERK1 (P-ERK1) antibody staining divided by the total fluorescence levels of ERK1 antibody staining and normalized to percent of phosphorylated-ERK1 of untreated cells. (**e**) SKMEL239 C4 melanoma cells left untreated or treated with increasing concentration of Trametinib for 3 hrs, and assayed with in-cell-western. Quantification and analysis of % phosphorylated-ERK as in (d). (**f**) Z-factor analysis of the in-cell-western assay in a 96-well plate using Trametinib as a positive control and Vemurafenib as a negative control and quantifying total fluorescence from phosphorylated-ERK1. Error bars in d-f represent mean ± SD from 5 (d) , 3 (e) and 15 (f) replicates from 3 independent experiments.

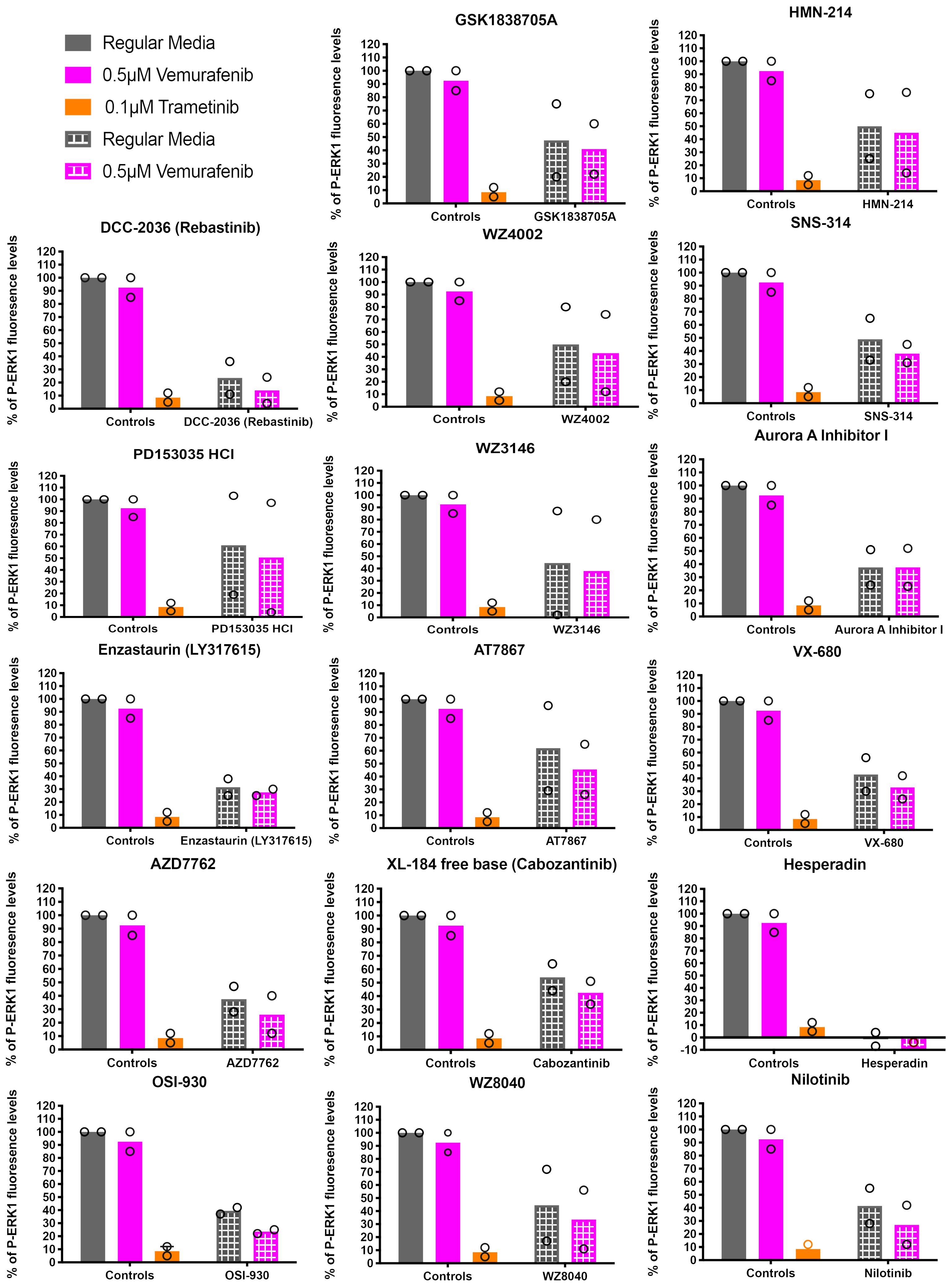

Regular Media
0.5µM Vemurafenib
0.1µM Trametinib
Regular Media
0.5µM Vemurafenib
GSK1838705A
HMN-214
DCC-2036 (Rebastinib)
WZ4002
SNS-314
PD153035 HCl
WZ3146
Aurora A Inhibitor I
Enzastaurin (LY317615)
AT7867
VX-680
AZD7762
XL-184 free base (Cabozantinib)
Hesperadin
OSI-930
WZ8040
Nilotinib
% of P-ERK1 fluoresence levels
Controls
Cabozantinib

Supplementary Figure 2. Validation of several kinase inhibitors hits from in-cell-western screen for inhibition of ERK signaling.

SKMEL239 C4 melanoma cells left untreated (regular media), treated with 0.5μM Vemurafenib, 0.1 μM Trametinib, and 5 μM kinase inhibitors hits from in-cell-western screen without or with 0.5 μM Vemurafenib for 3hrs and assayed with in-cell-western. Quantification for percent phosphorylated-ERK1 is calculated for different conditions. Bars represent mean from n=2 independent experiments.

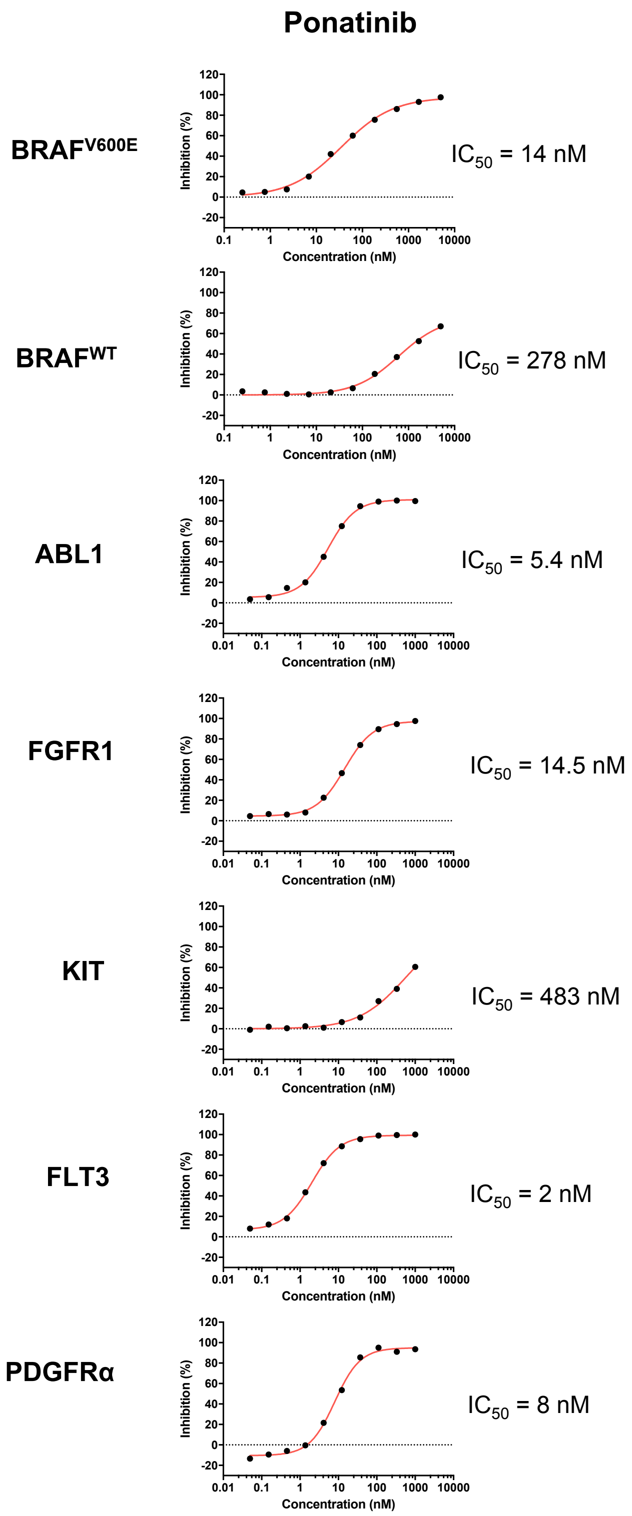
Ponatinib
BRAF^V600E
IC50 = 14 nM
BRAF^WT
IC50 = 278 nM
ABL1
IC50 = 5.4 nM
FGFR1
IC50 = 14.5 nM
KIT
IC50 = 483 nM
FLT3
IC50 = 2 nM
PDGFRα
IC50 = 8 nM
Inhibition (%)
Concentration (nM)

Supplementary Figure 3. In vitro kinase inhibition activity of Ponatinib.

Inhibition of kinase activity of $BRAF^{V600E}$, $BRAF^{WT}$ and other tyrosine kinases targets by Ponatinib using SelectScreen (Invitrogen) assay in the presence of 100 μM ATP. Half-maximal inhibition values (IC_{50}) in kinase activity by Ponatinib are measured. Data are mean ± SD of two technical replicates from n=2 independent experiments.

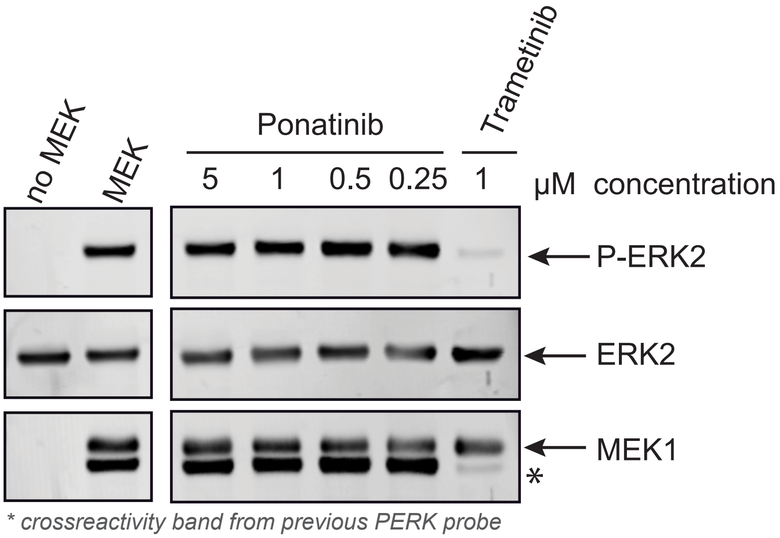
no MEK
MEK
Ponatinib
Trametinib
5
1
0.5
0.25
1
μM concentration
P-ERK2
ERK2
MEK1
*
* crossreactivity band from previous PERK probe

Supplementary Figure 4. Ponatinib does not inhibit MEK.

MEK kinase activity assay in the absence or presence of Ponatinib or Trametinib at the indicated doses, then assayed for western blot with the indicated antibodies. Data are representative of n=2 independent experiments.

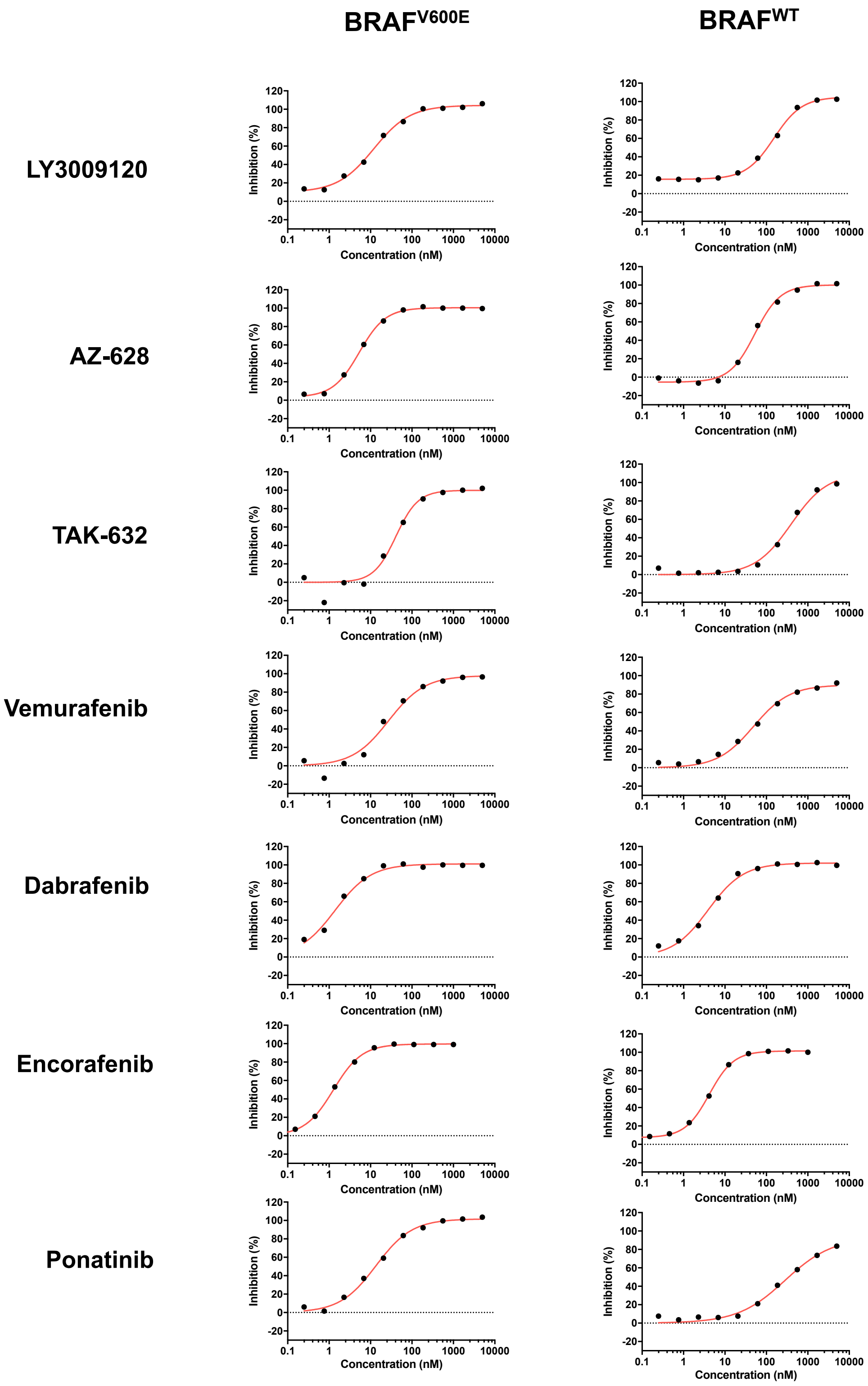

	BRAF^{V600E} IC$_{50}$ (nM)	BRAF^{WT} IC$_{50}$ (nM)
**Ponatinib**	14 ± 1	278 ± 68
**LY3009120**	12 ± 2	152 ± 15
**AZ-628**	5.3 ± 0.4	53 ± 4
**TAK-632**	36 ± 6	343 ± 43
**Vemurafenib**	22 ± 5	60 ± 5
**Dabrafenib**	1.9 ± 0.1	5.0 ± 0.2
**Encorafenib**	1.3 ± 0.1	4.2 ± 0.2

Supplementary Figure 5. In vitro BRAF kinase inhibition activity of RAF inhibitors. $BRAF^{WT}$ and $BRAF^{V600E}$ kinase inhibition by selected αC-IN inhibitors (LY3009120, AZ-628, TAK-632) and αC-OUT inhibitors (Vemurafenib, Dabrafenib, Encorafenib), in comparison to BRAF inhibition by Ponatinib as reported here. Kinase activity was measured using SelectScreen (Invitrogen) assay in the presence of 100 μM ATP. Half-maximal inhibition values (IC_{50}) in kinase activity are summarized on the table (bottom). Data are mean ± SD of two technical replicates from n=2 independent experiments.

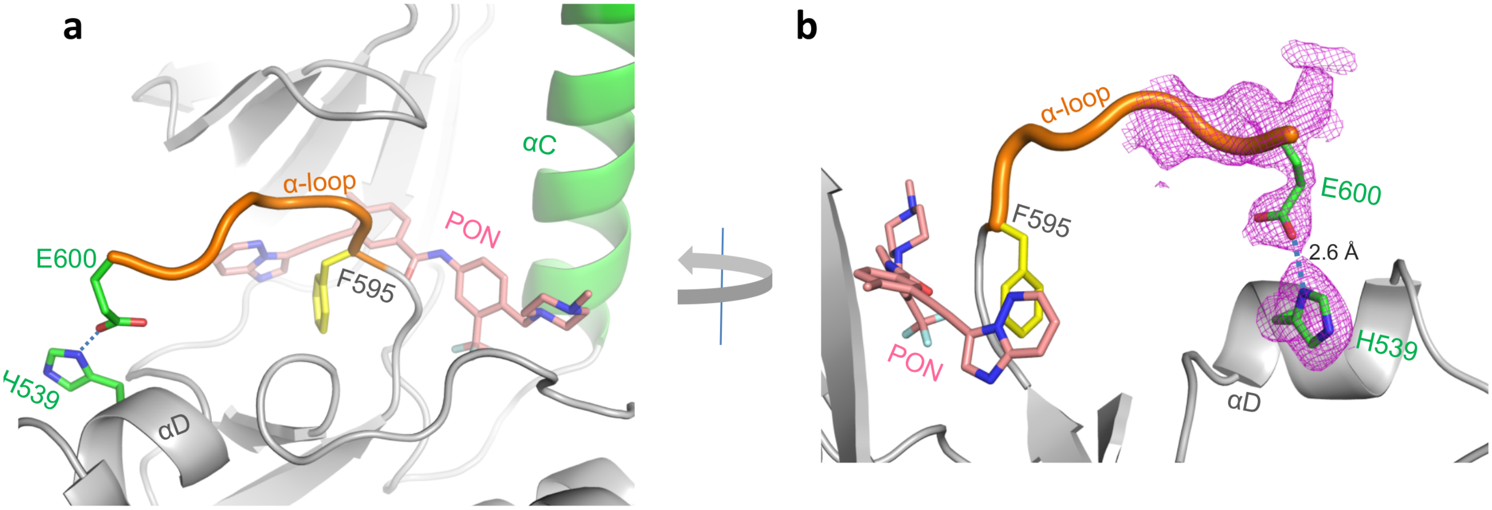
a
α-loop
αC
PON
E600
F595
H539
αD
b
α-loop
E600
F595
PON
2.6 Å
H539
αD

Supplementary Figure 6. Ponatinib (PON) induces a unique conformation in the activation loop when is bound to BRAF^{V600E}.

(**a**) The a-loop in BRAF^{V600E}-PON complex adopts a unique conformation that allows residue E600 to dock at a previously unobserved position in BRAF structures, where it forms a hydrogen bond with residue H539. **(b)** a rotated view (approximately 180°) of (a) highlighting the E600-H539 interaction. Electron density (2fo-fc) for above residues (including part of α-loop), contoured at 0.8σ, is superimposed.

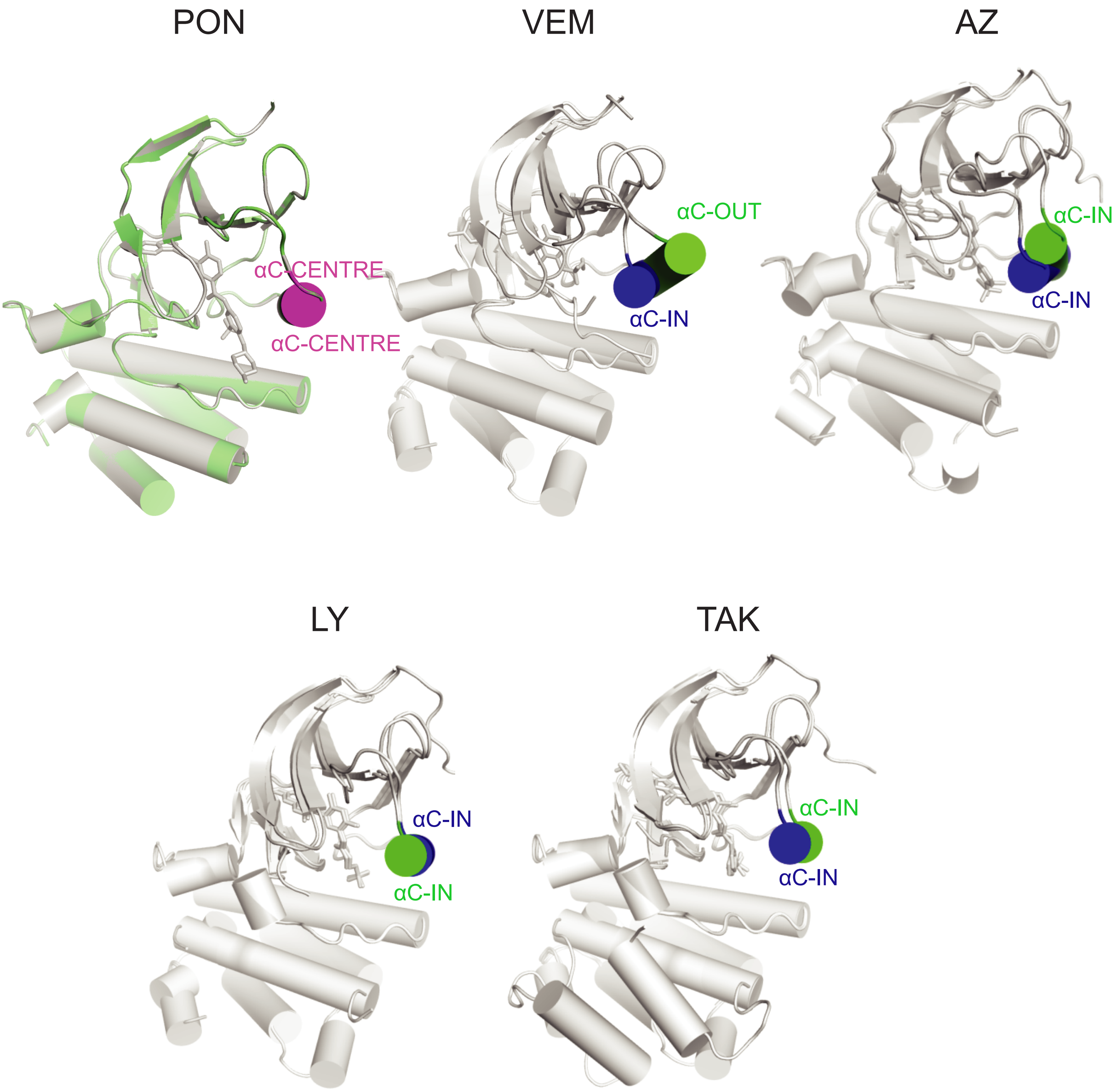
PON
αC-CENTRE
αC-CENTRE
VEM
αC-OUT
αC-IN
AZ
αC-IN
αC-IN
LY
αC-IN
αC-IN
TAK
αC-IN
αC-IN

Supplementary Figure 7. Comparison of BRAF dimers stabilized by Ponatinib (PON), Vemurafenib (VEM), AZ-628 (AZ), LY3009120 (LY) and TAK-632 (TAK).

The BRAFV600E/PON dimer is perfectly symmetric. 3D-superposition of each BRAFV600E/PON protomer in silver and green ribbons shows a perfect 2-fold symmetry. The perfectly overlapping αC-helices of protomers are shown in magenta. Asymmetry in αC-helix position is highlighted from the superposition of BRAF protomers in ribbon representations from complex structures of BRAF dimer with VEM (PDB: 3OG7), AZ-628 (PDB: 4G9R), LY (PDB: 5C9C) and TAK (PDB: 4KSP). Selected C-lobe helices have been omitted for clarity.

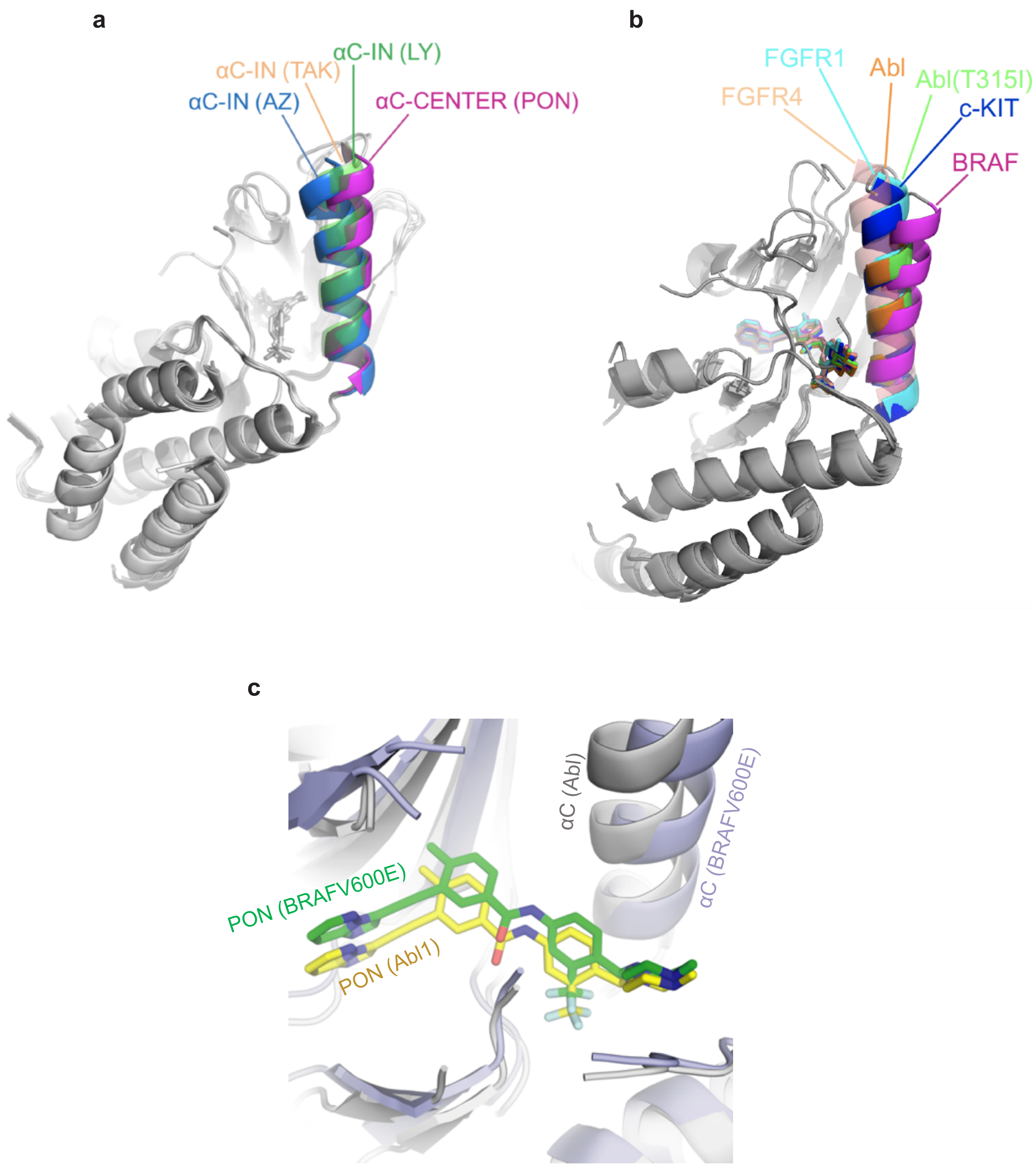
a
αC-IN (LY)
αC-IN (TAK)
αC-IN (AZ)
αC-CENTER (PON)
b
FGFR1
Abl
Abl(T315I)
FGFR4
c-KIT
BRAF
c
αC (Abl)
αC (BRAFV600E)
PON (BRAFV600E)
PON (Abl1)

Supplementary Figure 8. The αC helix of BRAF^{V600E}/Ponatinib complex adopts a distinct position compared to other RAF inhibitors with BRAF and Ponatinib with other kinases. (**a**) Structural superposition of BRAF structures in ribbon representation showing the position of αC-helix in BRAF^{V600E} structure bound to Ponatinib (PON, magenta) and αC-IN positions observed in AZ-628 (AZ, blue) (PDB: 4G9R), TAK-632 (TAK, orange) (PDB: 4KSP) and LY3009120 (LY, green) (PDB: 5C9C). Protein parts were omitted for clarity. (**b**) Structural superposition of ribbon structures showing the position of αC-helix in BRAF^{V600E} structure bound to Ponatinib (PON) (magenta), in Abl1 structure bound to Ponatinib (orange) (PDB:3OXZ), in Abl-T315I structure bound to Ponatinib (green) (PDB:3IK3) in FGFR1 structure bound to Ponatinib (cyan) (PDB: 4V04) in FGFR4 structure bound to Ponatinib (salmon) (PDB:4UXQ) and in cKIT structure bound to Ponatinib (blue) (PDB: 4U0I). Protein parts were omitted for clarity. (**c**) 3D-superposition of BRAF^{V600E}/PON and Abl1/PON (PDB: 3OXZ) complex structures, illustrating the binding mode of Ponatinib to the proteins. 3D-superposition was performed in Coot and was based on the C-terminal lobes the kinases, which demonstrate higher structural similarity.

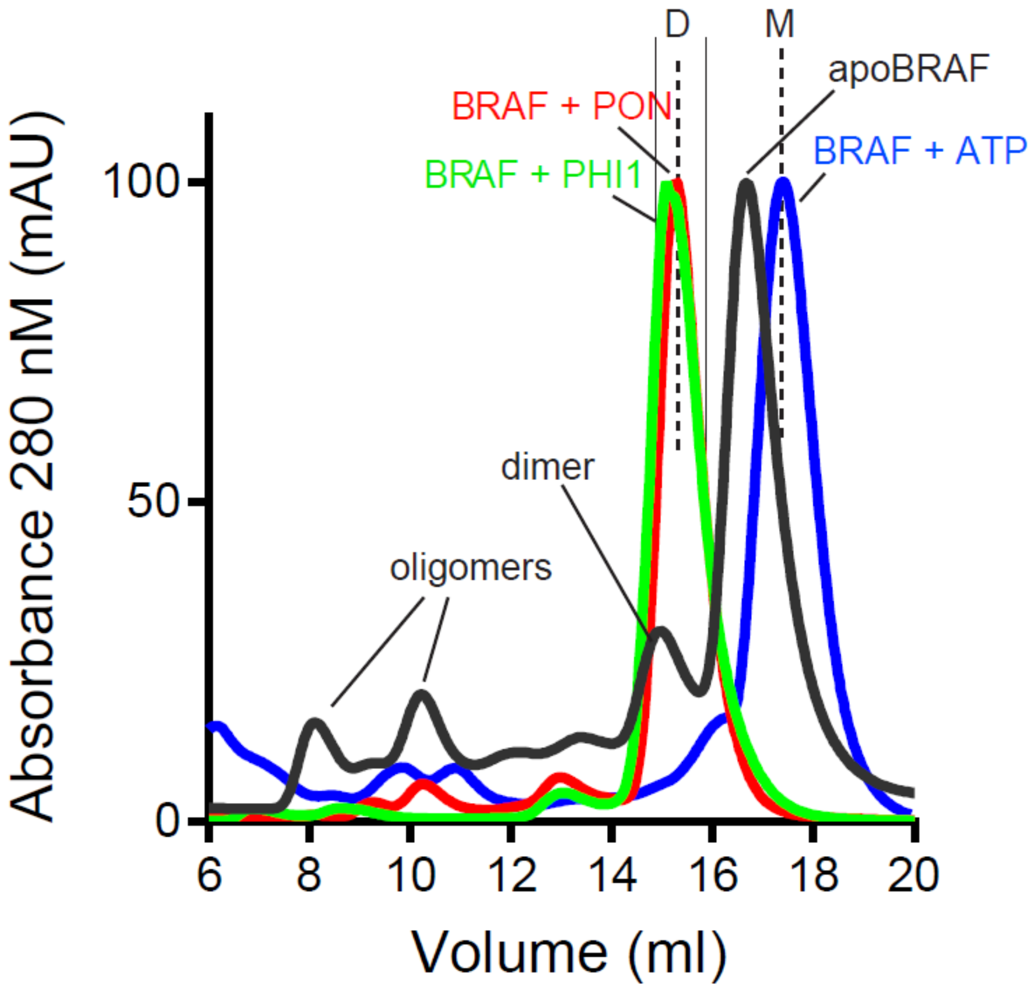

D
M
apoBRAF
BRAF + PON
BRAF + ATP
BRAF + PHI1
100
50
0
Absorbance 280 nM (mAU)
dimer
oligomers
6
8
10
12
14
16
18
20
Volume (ml)

Supplementary Figure 9. Size-exclusion chromatography analysis of apo-BRAF^{V600E} and in complex with ATP, Ponatinib or PHI1.

Size exclusion chromatographic profiles using Superdex-200 10/300 of recombinant apo-BRAF^{V600E} kinase domain (black curve) or upon addition of ATP (blue curve), Ponatinib (PON), (red curve) or PHI1 (green curve). Compounds were added immediately after Ni-NTA purification. ATP was at 1:2 protein to compound molar ratio, and PON/PHI1 at 1:1 protein to compound molar ratio. Monomeric and dimeric BRAF^{V600E} protein is marked as M and D, respectively. Data are representative of n=3 independent experiments.

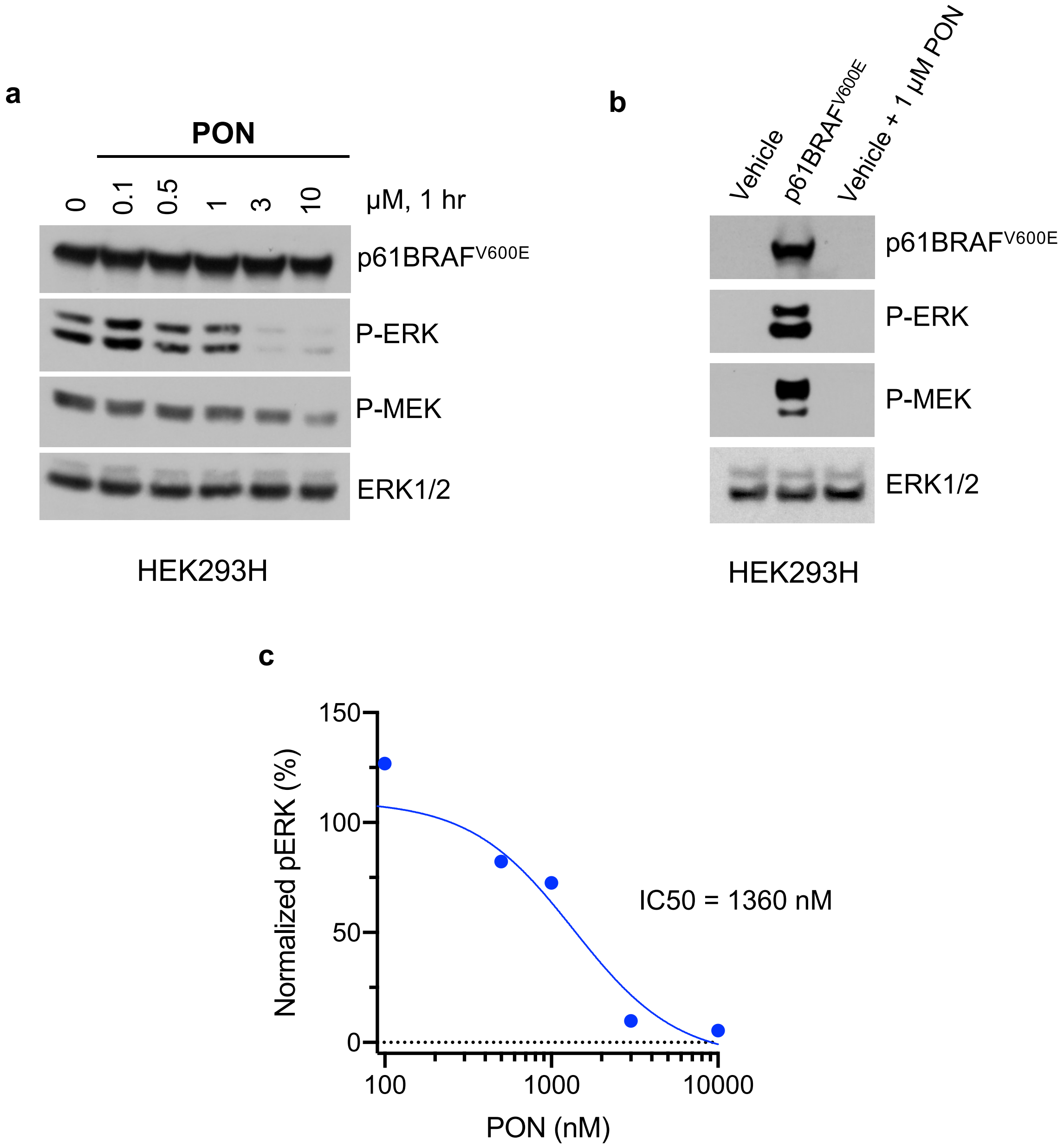

a
PON
0
0.1
0.5
1
3
10
μM, 1 hr
p61BRAFV600E
P-ERK
P-MEK
ERK1/2
HEK293H
b
Vehicle
p61BRAFV600E
Vehicle + 1 μM PON
p61BRAFV600E
P-ERK
P-MEK
ERK1/2
HEK293H
c
150
100
50
0
Normalized pERK (%)
IC50 = 1360 nM
100
1000
10000
PON (nM)

Supplementary Figure 10. Inhibition of ectopically expressed p61BRAF^{V600E} by Ponatinib (PON).

(**a**) HEK239H cells transiently expressing p61BRAF^{V600E} were treated with DMSO or increasing concentrations of Ponatinib for 1 hr. ERK-signaling and BRAF^{V600E} expression was assayed by western blot. A representative blot from n=2 independent experiments is shown. (**b**) absence of p61BRAF^{V600E} expression and pERK signaling in non-transfected cells treated similarly with DMSO or 1 μM Ponatinib (negative control). (**c**) normalized values of p-ERK levels with corresponding fitted curve. Data are mean ± SD of two replicates from n=2 independent experiments.

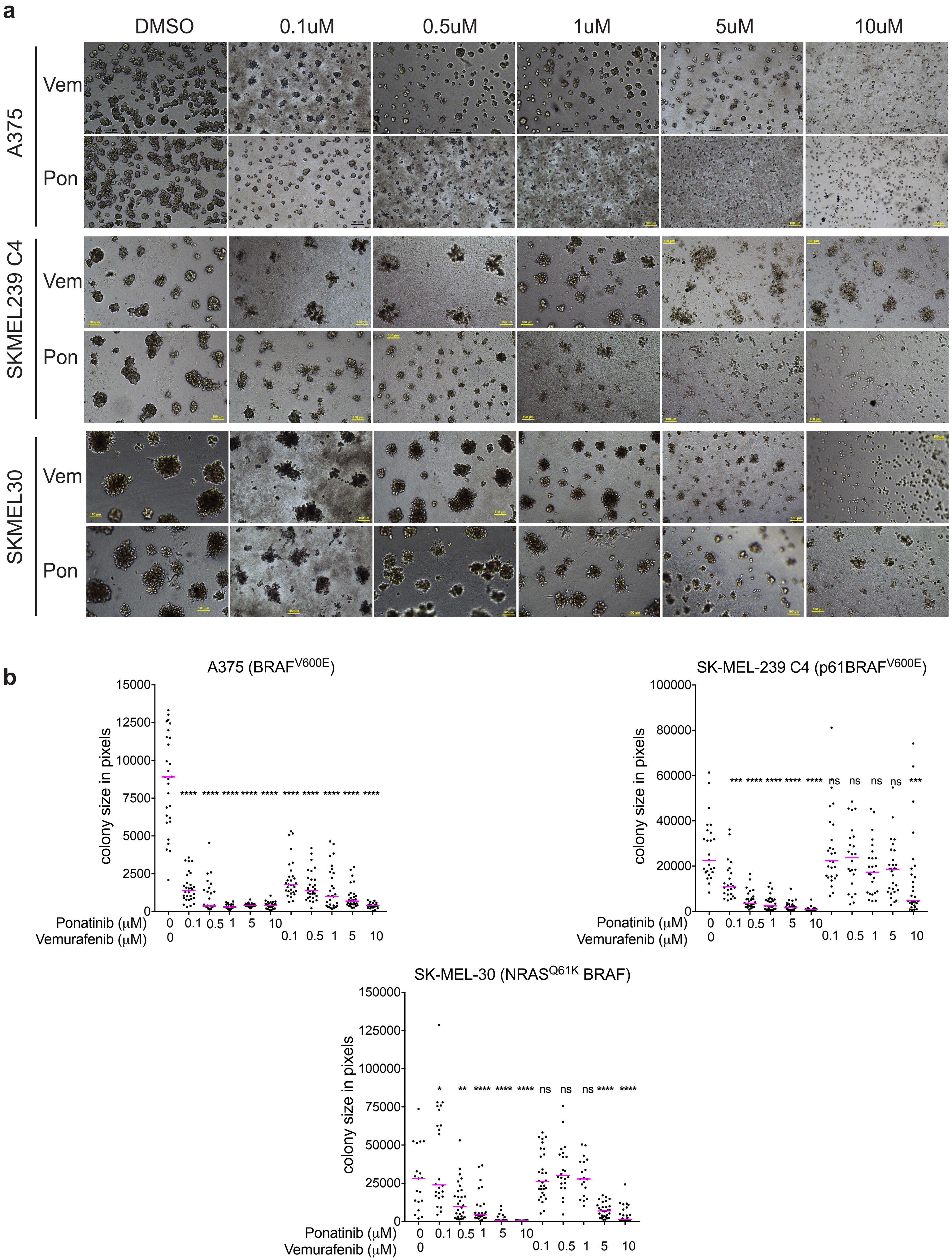
a
DMSO
0.1uM
0.5uM
1uM
5uM
10uM
A375
Vem
Pon
SKMEL239 C4
Vem
Pon
SKMEL30
Vem
Pon
b
A375 (BRAF^{V600E})
colony size in pixels
Ponatinib (μM)
Vemurafenib (μM)
SK-MEL-239 C4 (p61BRAF^{V600E})
colony size in pixels
Ponatinib (μM)
Vemurafenib (μM)
SK-MEL-30 (NRAS^{Q61K} BRAF)
colony size in pixels
Ponatinib (μM)
Vemurafenib (μM)

Supplementary Figure 11. Ponatinib treatment inhibits tumor cell growth in melanoma cells expressing different BRAF species.

(a) 3D Matrigel cell-culture of melanoma A375, SK-MEL-30 and SK-MEL-239 C4 cells cultured with increasing dose of Ponatinib (Pon) or Vemurafenib (Vem). Pictures taken on the 5th day of culture, scale bar is set at 100 μm. Data are representative of n=3 independent experiments **(b)** Quantitative analysis of 3D colony size as depicted by scatter plots, where the blue line is the median colony size in pixels. All statistical analysis of 3D colony size: one-way ANOVA. Statistical significance of treatments with A375 cells **** $P \leq 0.0001$, with SK-MEL-30 cells *$P = 0.0204$, **$P = 0.0038$, ****$P \leq 0.0001$, with SK-MEL-239 C4 cells *$P = 0.029$, ***$P = 0.0002$, ****$P \leq 0.0001$ Data are representative of n=3 independent experiments.

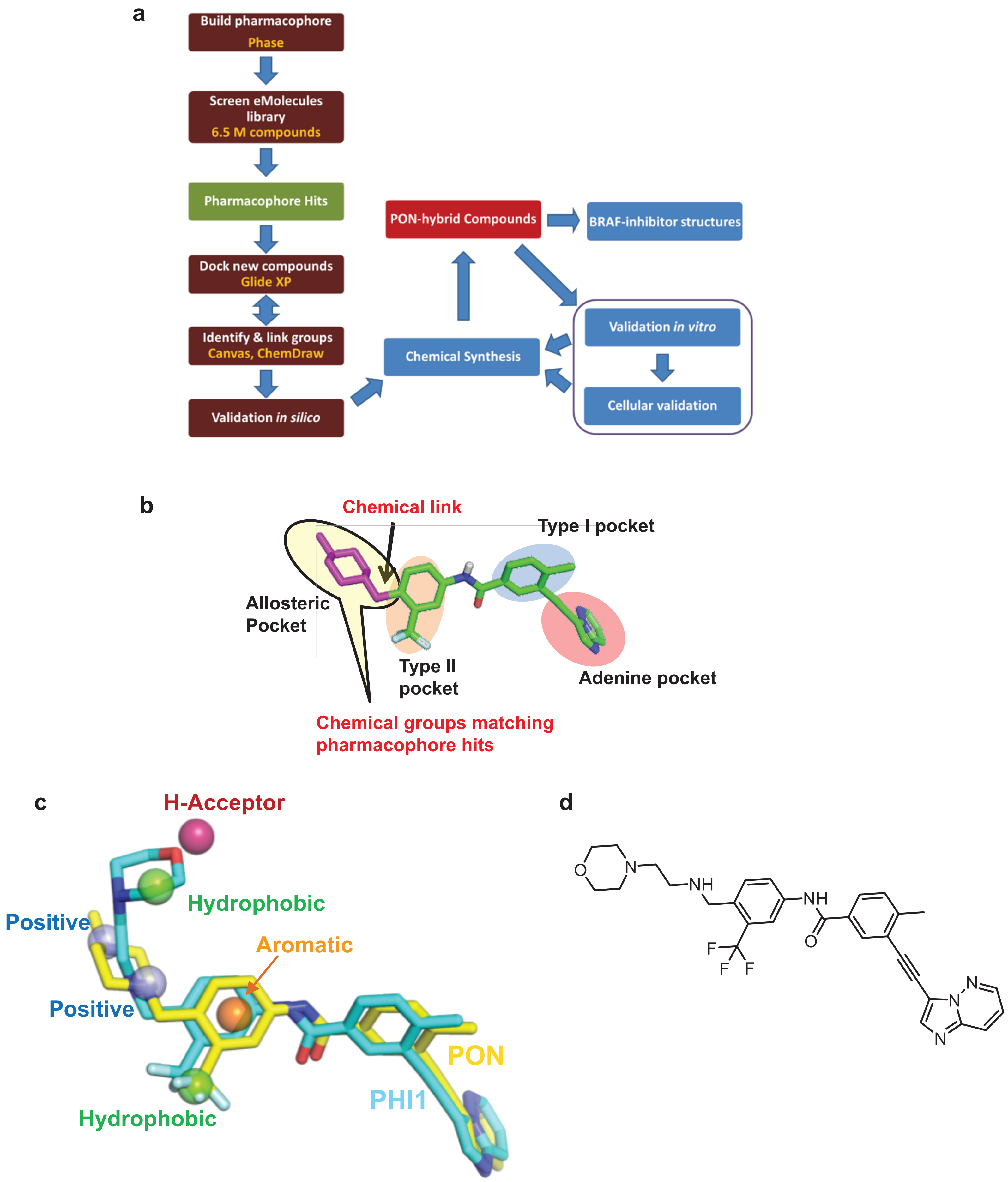
a
Build pharmacophore
Phase
Screen eMolecules library
6.5 M compounds
Pharmacophore Hits
Dock new compounds
Glide XP
Identify & link groups
Canvas, ChemDraw
Validation in silico
Chemical Synthesis
PON-hybrid Compounds
BRAF-inhibitor structures
Validation in vitro
Cellular validation
b
Chemical link
Type I pocket
Allosteric Pocket
Type II pocket
Adenine pocket
Chemical groups matching pharmacophore hits
c
H-Acceptor
Hydrophobic
Positive
Aromatic
Positive
PON
PHI1
Hydrophobic
d
O
N
NH
NH
O
F
F
F
N
N
N

Supplementary Figure 12. Structure-based drug design of Ponatinib-hybrid inhibitors (PHIs) and discovery of PH1.

(**a**) Cartoon flow diagram of a rational drug-design approach leading to Ponatinib-hybrid inhibitors targeting additional interactions with the allosteric pocket. (**b**) Schematic showing the synthetic strategy to efficiently generate PON-hybrid compounds by joining hits from the 3D pharmacophore search to the Ponatinib core scaffold. (**c**) The 3D pharmacophore model hypothesis includes seven potential criteria (six spheres) superimposed to Ponatinib and PHI1 (in sticks) in their bound conformation to $BRAF^{V600E}$ that was used for screening candidate ponatinib-hybrid inhibitors. (**d**) The molecular structure of PHI1.

PHI1

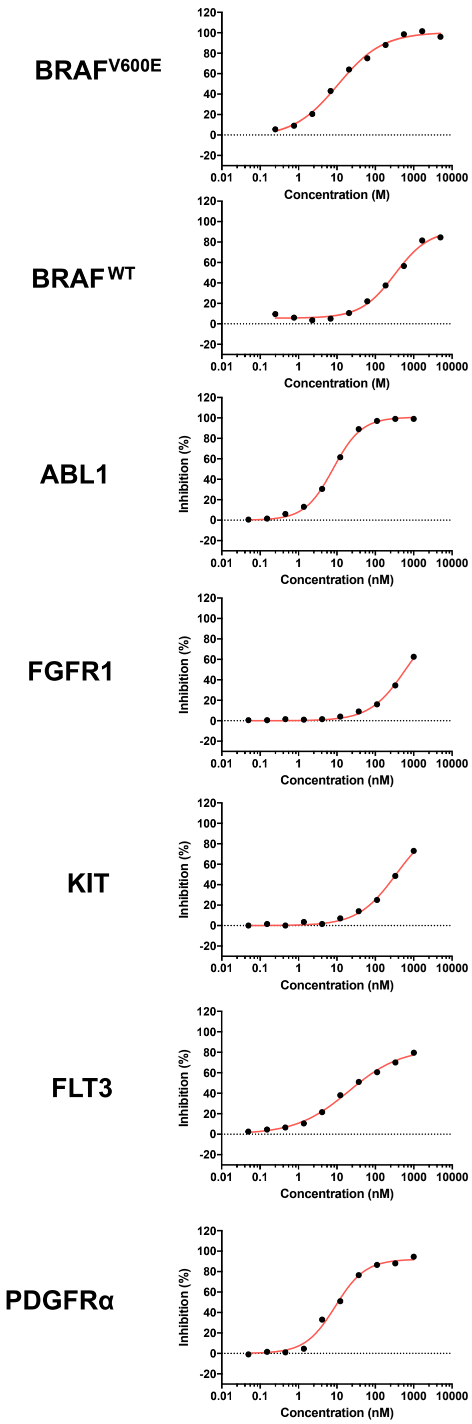

	PON IC_{50} (nM)	PHI1 IC_{50} (nM)
BRAF^{V600E}	14 ± 1	10 ± 2
BRAF^{WT}	278 ± 67	323 ± 35
ABL1	5.4 ± 0.4	8.4 ± 0.3
FGFR1	14.1 ± 0.7	624 ± 25
FLT3	2.0 ± 0.1	20.5 ± 2.7
KIT	483 ± 39	548 ± 21
PDGFRα	8.0 ± 1.3	8.3 ± 1.5

Supplementary Figure 13. In vitro kinase inhibition activity of PHI1.

Inhibition of kinase activity of BRAF and selected RTKs by PHI1 using SelectScreen (Invitrogen) assay in the presence of 100 μM ATP. Half-maximal inhibition (IC_{50}) of kinase activities by PHI1 and Ponatinib (PON) are tabulated. Data are mean ± SD of two technical replicates from n=2 independent experiments.

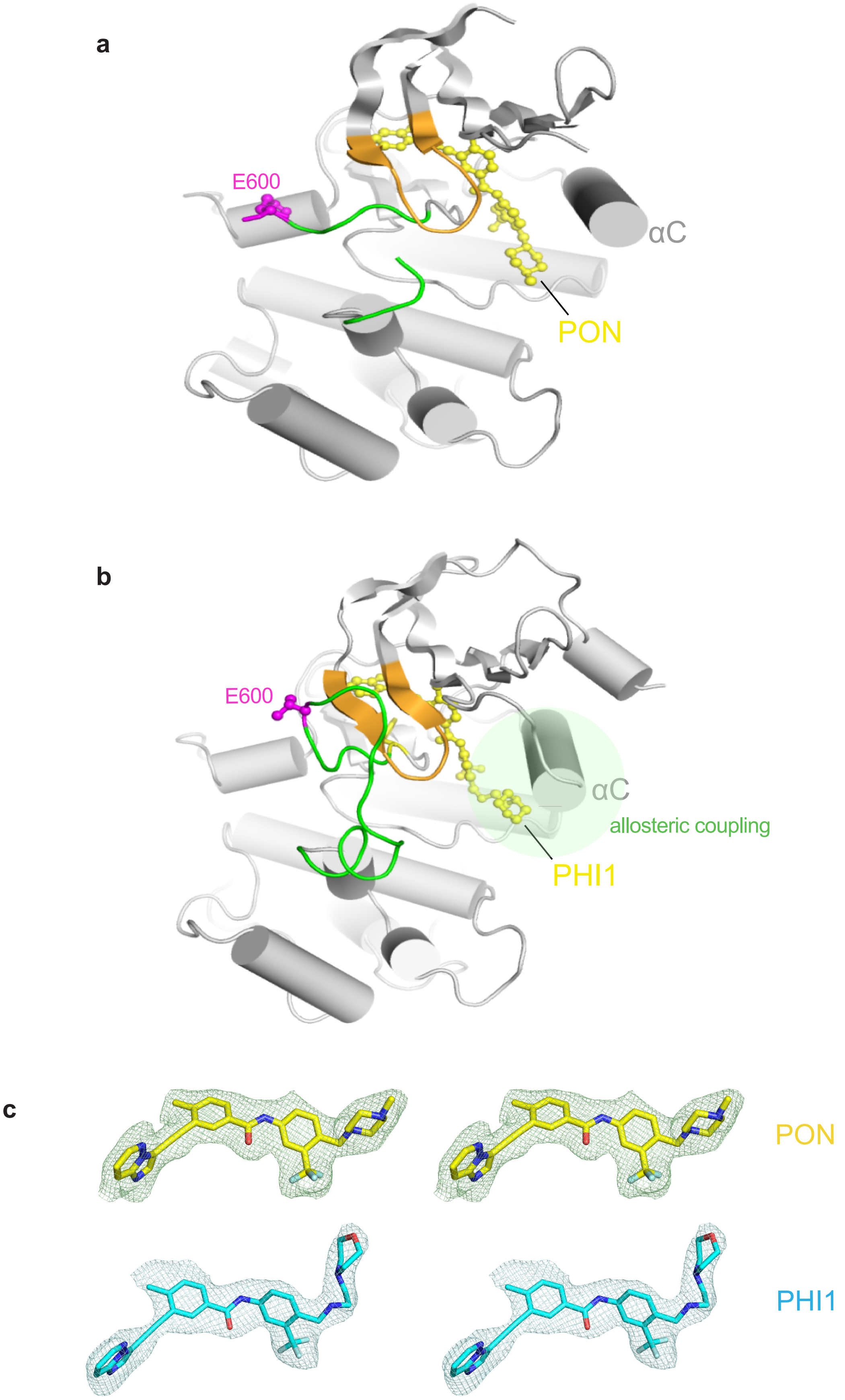
a
E600
αC
PON
b
E600
αC
allosteric coupling
PHI1
c
PON
PHI1

Supplementary Figure 14. Comparison of BRAF^{V600E}/PHI1 and BRAF^{V600E}/PON structures.

An overview of BRAF^{V600E}/PON (**a**) and BRAF^{V600E}/PHI1 (**b**) structures, showing bound inhibitors (yellow), p-loop (orange) and the kinase activation loop (green) with residue E600 (magenta). PHI1 binding induces an allosteric αC-helix movement (allosteric coupling) and a distinct, among RAF structures, orientation of the activation loop. (**c**) Stereo images of omit electron density (Fo-Fc) of PON (3σ) and PHI1 (2σ) is shown superimposed to stick models of the compounds.

a

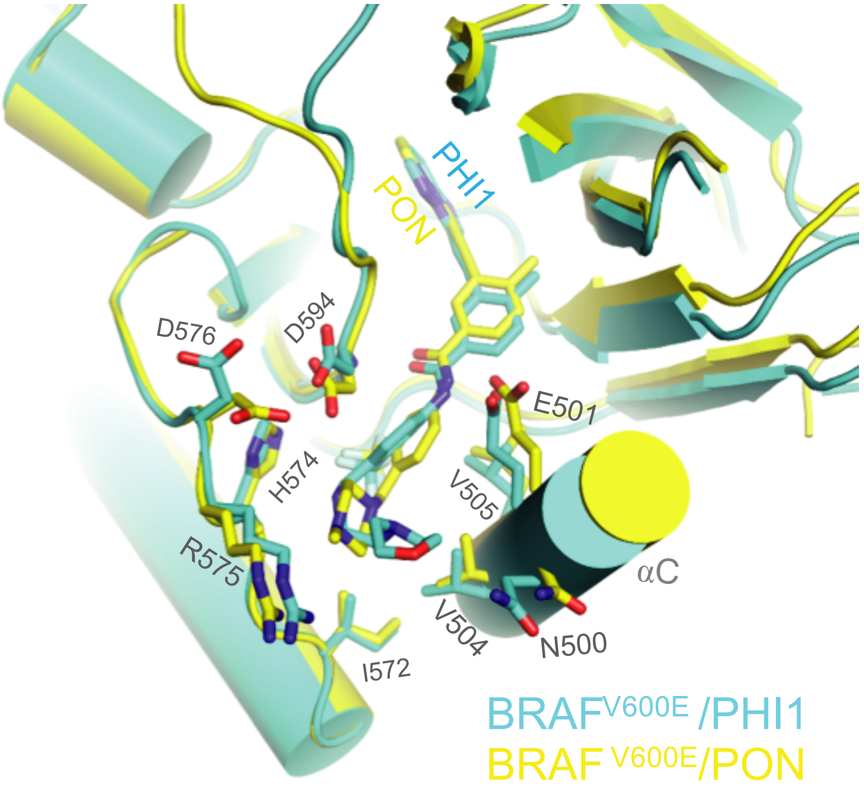

PHI1
PON
D576
D594
E501
H574
V505
R575
αC
V504
N500
I572
BRAF^{V600E}/PHI1
BRAF^{V600E}/PON

b

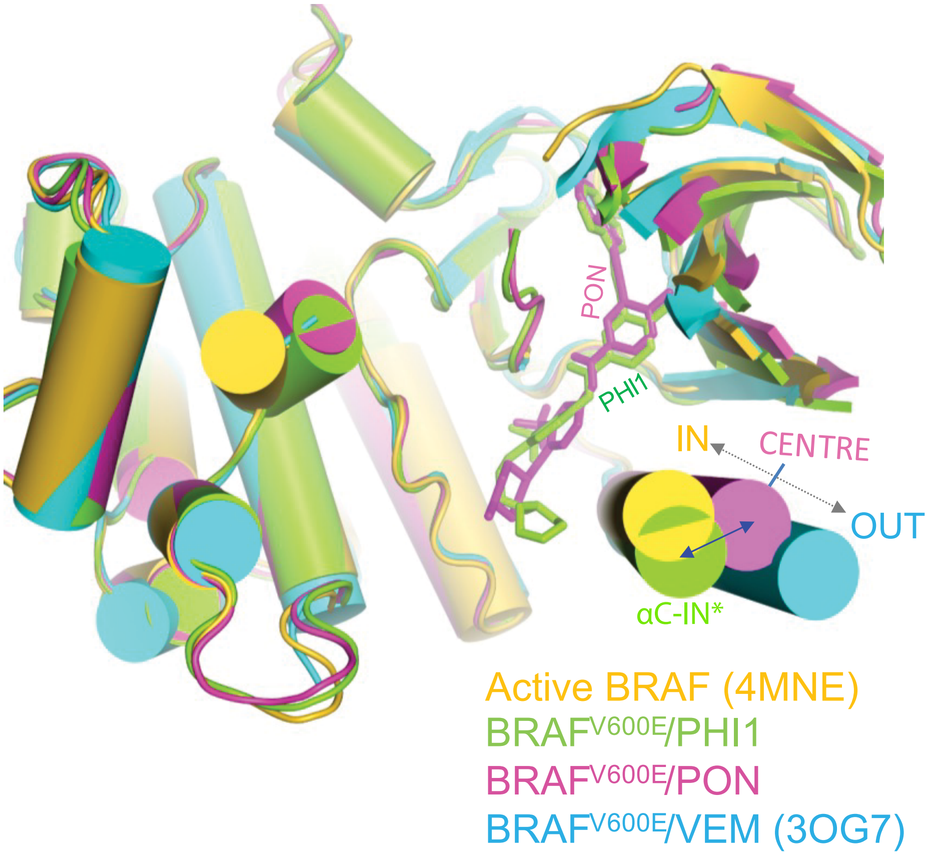

PON
PHI1
IN
CENTRE
OUT
αC-IN*
Active BRAF (4MNE)
BRAF^{V600E}/PHI1
BRAF^{V600E}/PON
BRAF^{V600E}/VEM (3OG7)

Supplementary Figure 15. Comparison of BRAF^{V600E}/PHI1 and BRAF^{V600E}/PON structures highlighting major differences.

(**a**) Comparison between BRAF^{V600E} structures bound to PHI1 and Ponatinib (PON). Local structural rearrangement at the vicinity of αC-helix upon PHI1 binding to BRAF^{V600E} compared to BRAF^{V600E}/PON structure. 3D-superpositions were based on the kinase C-lobe. Protein parts are omitted for clarity. (**b**) Comparison of BRAF^{V600E} structures bound to PHI1 and PON with typical αC-IN and αC-OUT BRAF conformations. PHI1 binding induces a distinct αC-IN* conformation.

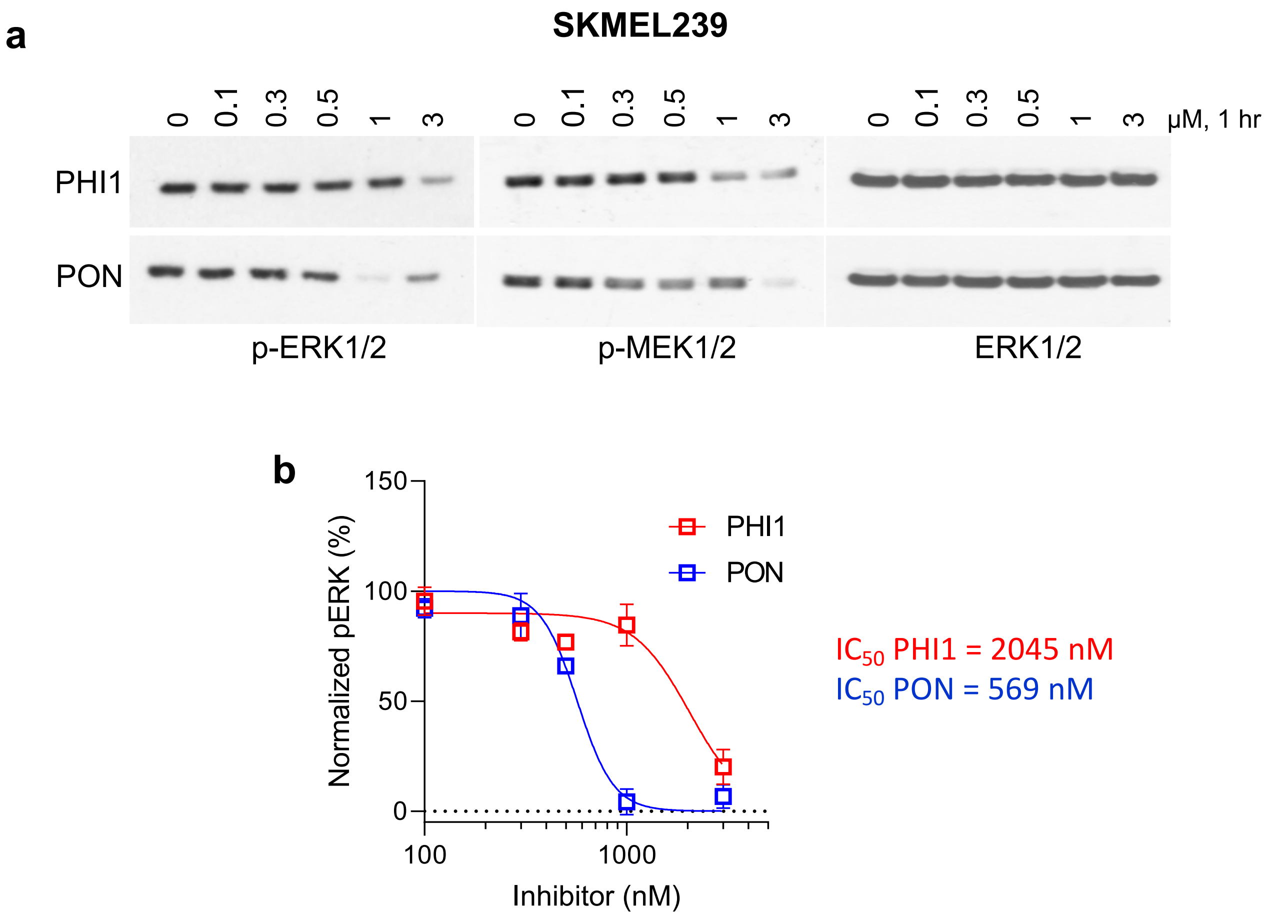
a
SKMEL239
0
0.1
0.3
0.5
1
3
µM, 1 hr
PHI1
PON
p-ERK1/2
p-MEK1/2
ERK1/2
b
150
100
50
0
Normalized pERK (%)
PHI1
PON
100
1000
Inhibitor (nM)
IC50 PHI1 = 2045 nM
IC50 PON = 569 nM

Supplementary Figure 16. Inhibition of ERK-signaling by PHI1 and Ponatinib (PON) in SKMEL239 parental cells.

(**a**) Melanoma SKMEL239 parental cell line were treated with increasing concentrations of PHI1 or Ponatinib for 1hr. Whole cell lysates were assayed by western blot with the indicated antibodies to assess ERK-pathway inhibition. Representative blots from n=2 independent experiments are shown. (b) Normalized values of p-ERK levels obtained by densitometry with corresponding fitted curves. Data are mean ± SEM of two replicates from n=2 independent experiments.

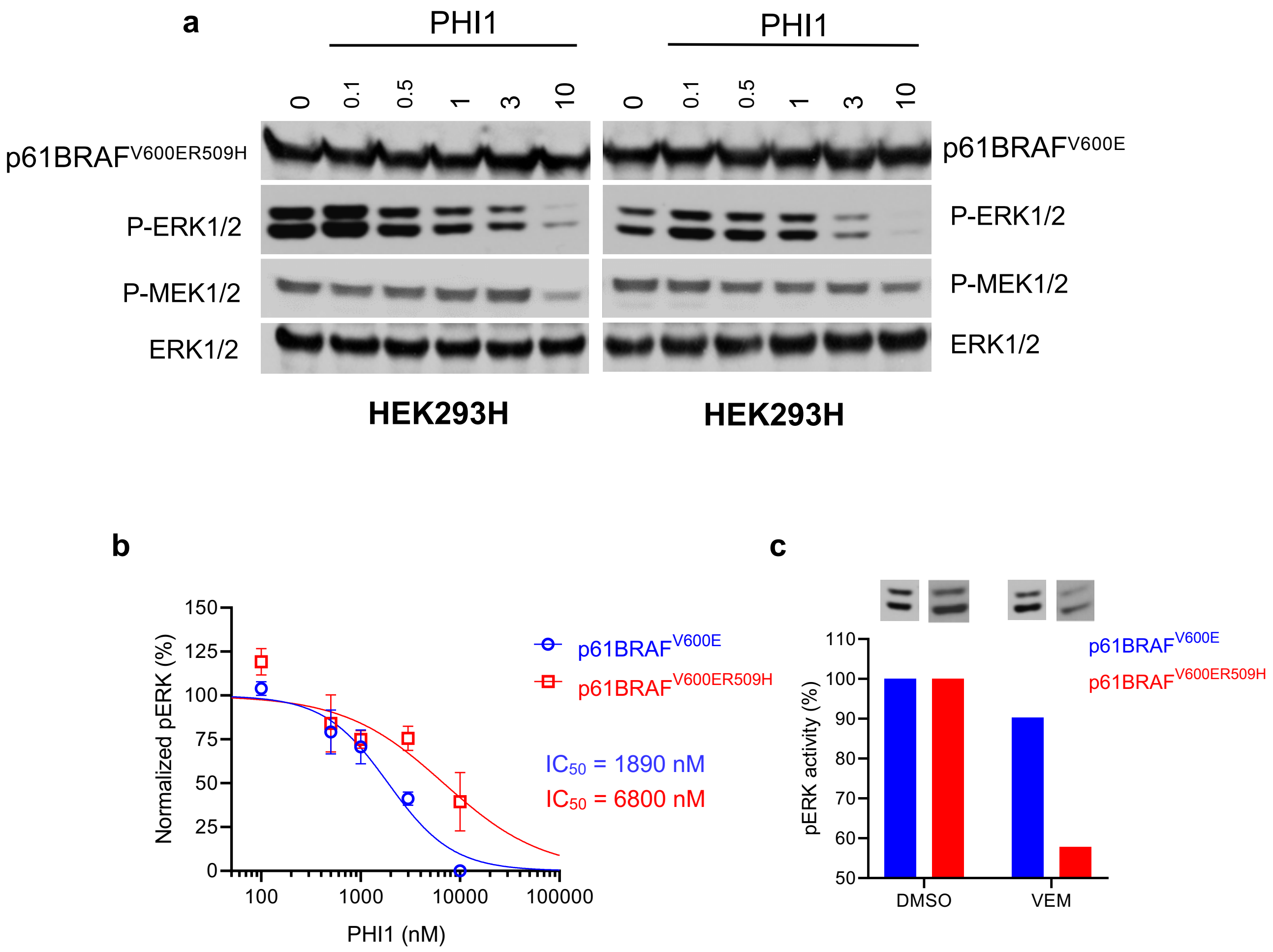
a
PHI1
0
0.1
0.5
1
3
10
p61BRAF V600ER509H
P-ERK1/2
P-MEK1/2
ERK1/2
p61BRAF V600E
HEK293H
HEK293H
b
Normalized pERK (%)
PHI1 (nM)
p61BRAF V600E
p61BRAF V600ER509H
IC50 = 1890 nM
IC50 = 6800 nM
c
pERK activity (%)
DMSO
VEM

Supplementary Figure 17. Inhibition of ectopically expressed p61BRAF^{V600E} and p61BRAF^{V600ER509H} by PHI1 shows specificity for p61BRAF^{V600E} dimer species.

(**a**) HEK239H cells transiently expressing p61BRAF^{V600E} or p61BRAF^{V600ER509H} were pretreated for 1 hr with 1 μM lapatinib to attenuate RTK signaling, followed by treatment with DMSO or increasing concentrations of PHI1 for another 1 hr. ERK-signaling and BRAF expression were assayed by western blot. A representative blot from n=2 independent experiments is shown. (**b**) quantification of pERK inhibition. Data are mean ± SEM of two replicates from n=2 independent experiments. (**c**) control experiment demonstrating preferential pERK-signaling inhibition by Vemurafenib (VEM) (at 10 μM) in p61BRAF^{V600ER509H} (monomer) transfected cells compared to p61BRAF^{V600E} (dimer) transfected cells.

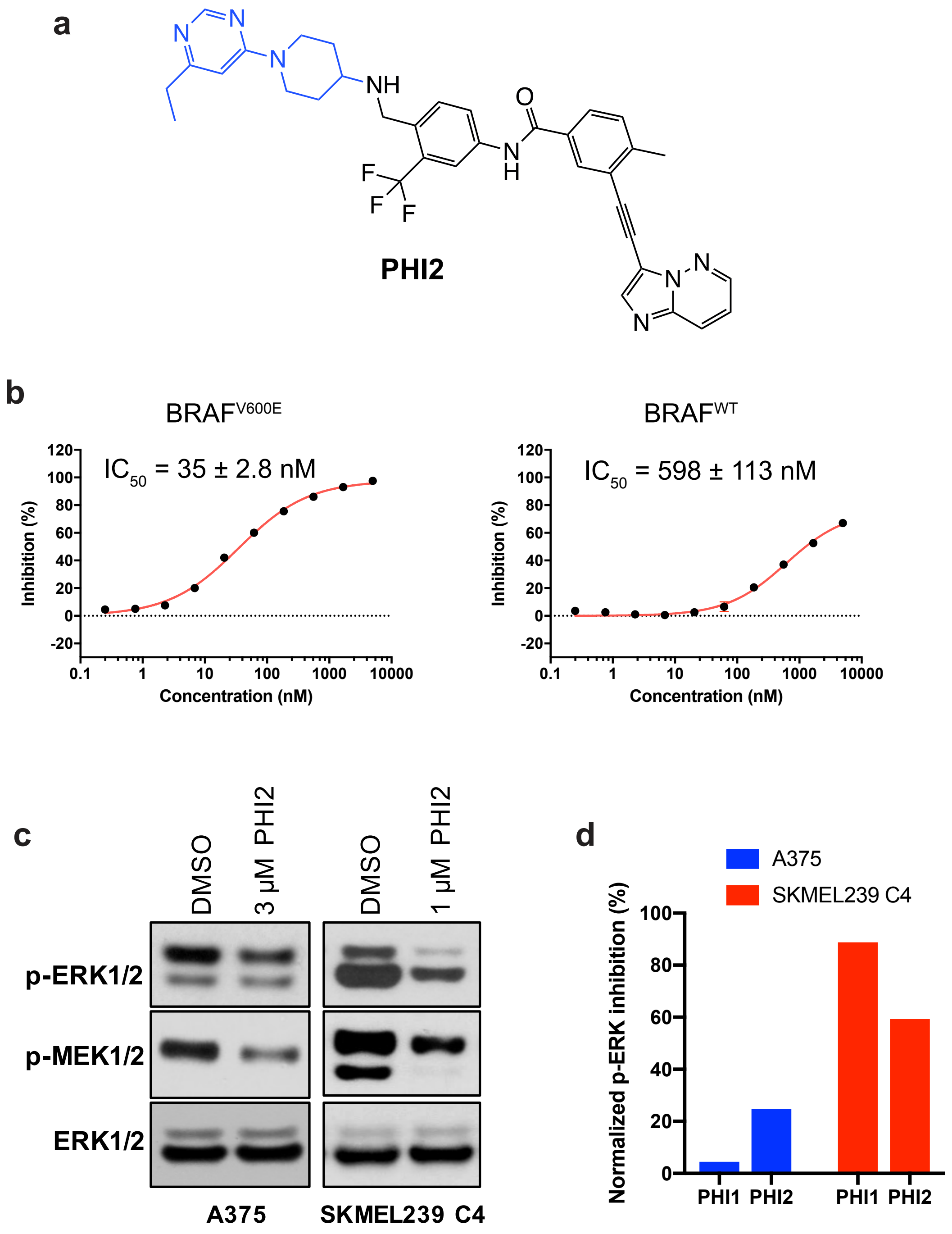
a
N
N
N
NH
O
N
H
F
F
F
N
N
N
PHI2
b
BRAF^V600E
BRAF^WT
IC50 = 35 ± 2.8 nM
IC50 = 598 ± 113 nM
Inhibition (%)
Concentration (nM)
120
100
80
60
40
20
0
-20
0.1
1
10
100
1000
10000
c
DMSO
3 μM PHI2
DMSO
1 μM PHI2
p-ERK1/2
p-MEK1/2
ERK1/2
A375
SKMEL239 C4
d
A375
SKMEL239 C4
Normalized p-ERK inhibition (%)
PHI1
PHI2
PHI1
PHI2

Supplementary Figure 18. PHI2 is an analogue of PHI1 with distinct binding specificity for BRAF^{V600E} monomers and dimers compared to PHI1.

(**a**) The molecular structure of PHI2. (**b**) BRAF kinase inhibition profiles (SelectScreen, Invitrogen) of PHI2. Data are mean ± SEM of two technical replicates from n=2 independent experiments. (**c**) Melanoma A375 and SKMEL239 C4 cells were treated with 1 μM PHI2, respectively, for 1hr. Whole cell lysates were assayed by western blot assay and ERK-pathway inhibition was assessed with indicated antibodies. Representative blots from two independent experiments are shown. (**d**) Quantitation of the effect of PHI2 on p-ERK from **c** (mean activities), in comparison to PHI. Mean activities from data used in Fig. 6a,b.

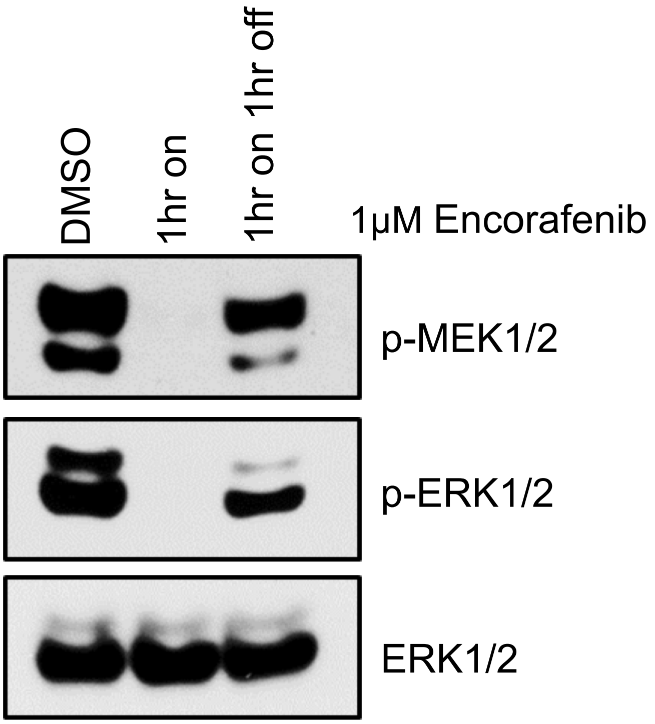
DMSO
1hr on
1hr on 1hr off
1µM Encorafenib
p-MEK1/2
p-ERK1/2
ERK1/2

SKMEL239-C4

Supplementary Figure 19. Recovery of ERK-signaling after Encorafenib wash out in SKMEL239 C4 cells.

(**a**) Melanoma SKMEL239 C4 cell lines were treated with 1 μM Encorafenib for 1 hr, followed by washed out with fresh medium for 1 hr. Untreated (DMSO), Encorafenib treated and washed-out whole cell lysates were assayed by western blot with indicated antibodies. Representative blots from n=2 independent experiments are shown.

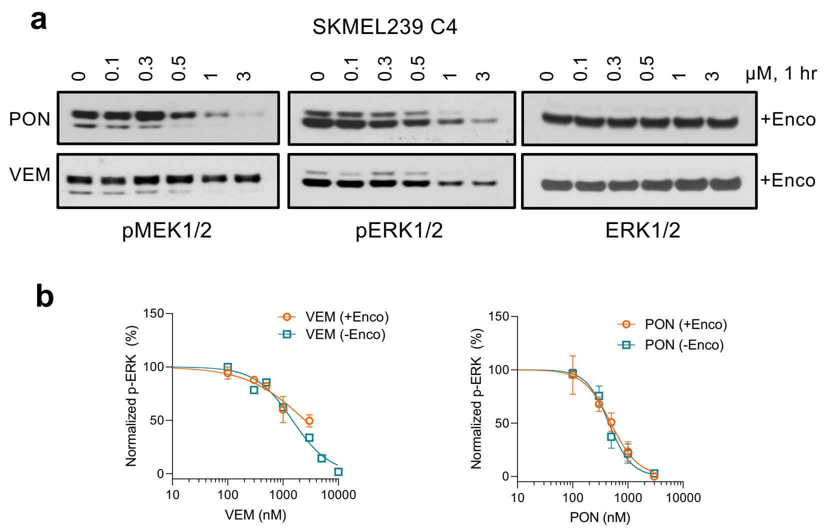
a
SKMEL239 C4
0
0.1
0.3
0.5
1
3
μM, 1 hr
PON
VEM
+Enco
+Enco
pMEK1/2
pERK1/2
ERK1/2
b
Normalized p-ERK (%)
VEM (+Enco)
VEM (-Enco)
VEM (nM)
PON (+Enco)
PON (-Enco)
PON (nM)
150
100
50
0
10
100
1000
10000

Supplementary Figure 20. Inhibition of second site in p61BRAF^{V600E} dimer by Ponatinib (PON) and Vemurafenib (VEM).

(**a**) SKMEL239 C4 cells pretreated with Encorafenib (Enco) for 1 hr followed by exchange with fresh medium for another hour, resulting in half-occupancy of p61BRAF^{V600E} dimer, were treated with increasing concentrations of Ponatinib and Vemurafenib for 1 hr and ERK-signaling was assayed by western blot. A representative blot from n=3 (Ponatinib) and n=2 (Vemurafenib) independent experiments is shown. (**b**) Normalized values and nonlinear regression fits of p-ERK activity shown in (**a**). Data are mean ± SEM (ponatinib) and mean (vemurafenib). Quantification of p-ERK activity of SKMEL239 C4 cells treated with Vemurafenib and Ponatinib without Encorafenib pretreatment is based on Fig. 4a and Fig. 6a respectively.

a

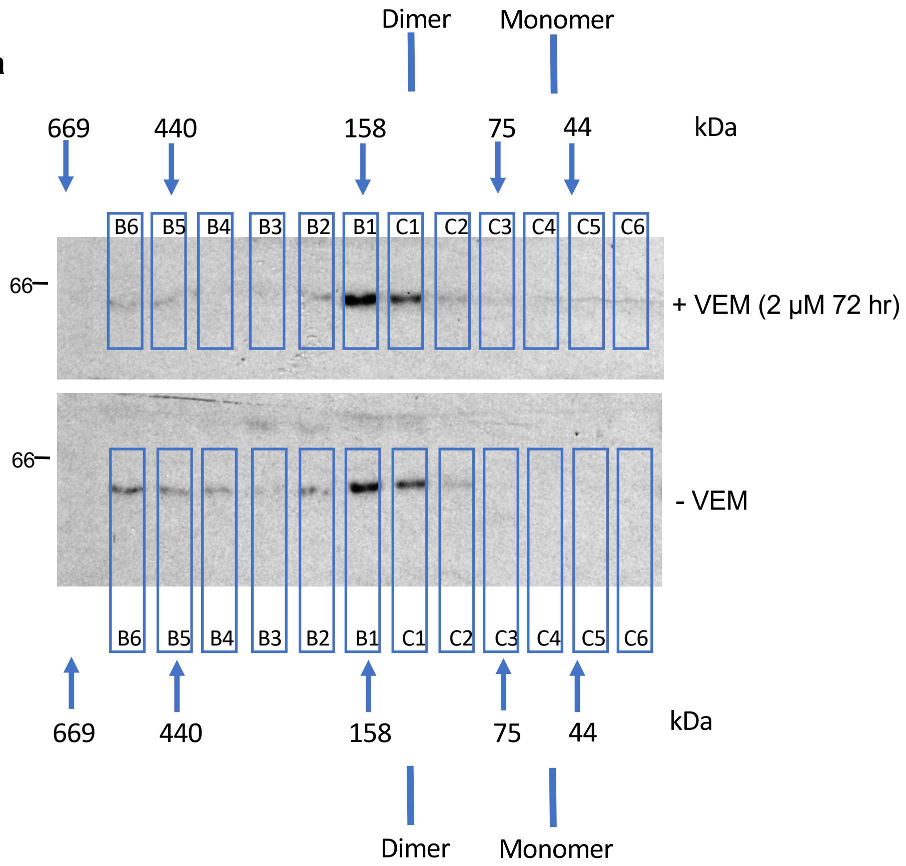

Dimer
Monomer
669
440
158
75
44
kDa
B6
B5
B4
B3
B2
B1
C1
C2
C3
C4
C5
C6
66
+ VEM (2 µM 72 hr)
66
- VEM
B6
B5
B4
B3
B2
B1
C1
C2
C3
C4
C5
C6
669
440
158
75
44
kDa
Dimer
Monomer

b

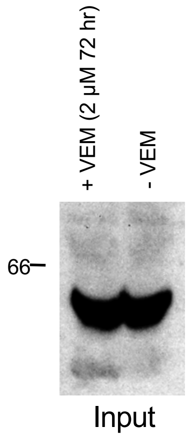

+ VEM (2 µM 72 hr)
- VEM
66
Input

Supplementary Figure 21. Size-exclusion chromatography analysis of p61BRAF^{V600E} in SKMEL239C4 cell extracts with and without Vemurafenib (VEM) treatment.

(**a**) Cell extracts (2.5 mg total protein per run) from SKMEL239 C4 cells without VEM or after treatment with 2 μM VEM for 72 hr were analyzed using a Superdex-200 10/300 column. Fractions flanking MWs corresponding to p61BRAF^{V600E} dimers or monomers (marked by lines) were collected and assayed by western blot using an antibody against BRAF^{V600E}. In both cases, no monomeric p61BRAF^{V600E} species were detected. (**b**) Western blot of input cell extracts used for chromatography runs shown in (**a**). Data are representative of n=2 experiments.

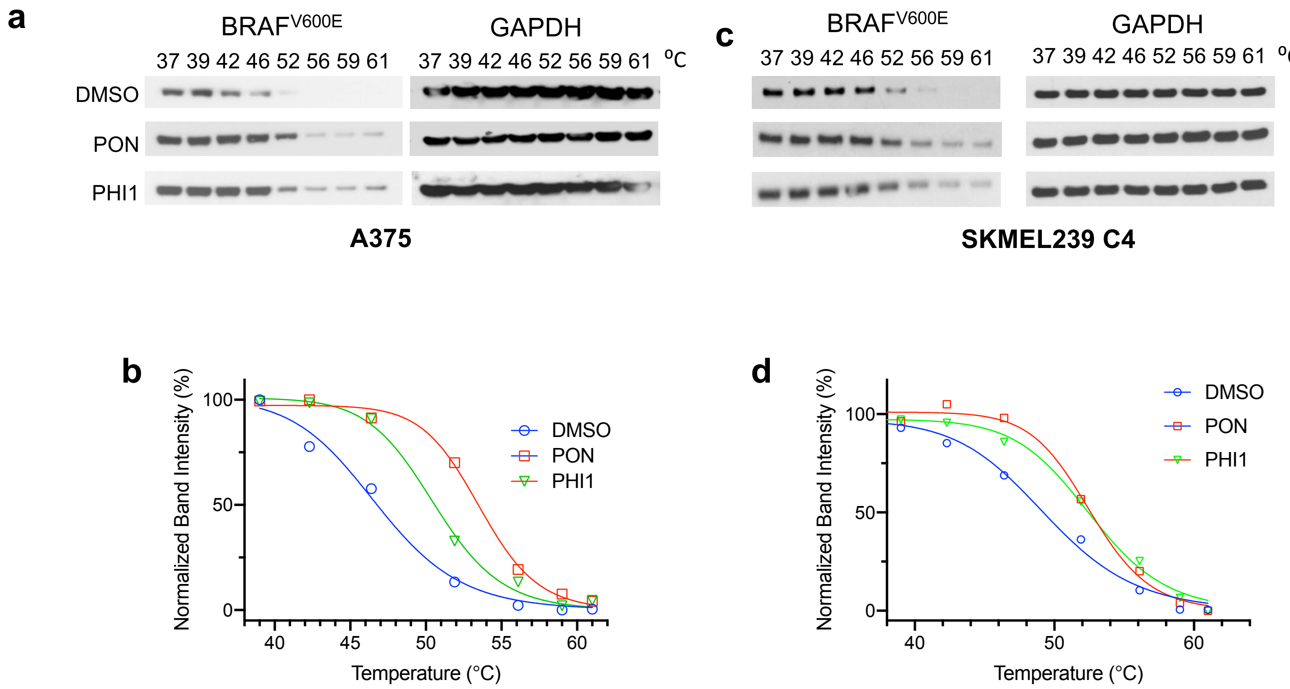

a
BRAF^{V600E}
GAPDH
37 39 42 46 52 56 59 61
37 39 42 46 52 56 59 61 °C
DMSO
PON
PHI1
A375
c
BRAF^{V600E}
GAPDH
37 39 42 46 52 56 59 61
37 39 42 46 52 56 59 61 °C
SKMEL239 C4
b
Normalized Band Intensity (%)
Temperature (°C)
DMSO
PON
PHI1
d
Normalized Band Intensity (%)
Temperature (°C)
DMSO
PON
PHI1

Supplementary Figure 22. Cellular engagement of BRAF by Ponatinib (PON) and PHI1.

Cellular engagement of BRAF by PON (**a**) and PHI1 (**c**) vs. DMSO control in A375 and SKMEL239 C4 cells, analyzed by CETSA. Representative blots from n=2 independent experiments are shown. (**b, d**) Quantitation of temperature-dependent normalized BRAF levels obtained by densitometry with corresponding fitted curves. Data are mean of n=2 independent experiments.

Supplementary Table 1: Statistics of data collection and refinement

	BRAF^{V600E}/PON [a]	BRAF^{V600E}/PHI1 [a]
**Data collection**		
Space group	$P4_322$	$P2_12_12_1$
Cell dimensions		
a, *b*, *c* (Å)	118.84, 118.84, 51.53	95.78, 108.79, 126.38
α, β, γ (°)	90.0, 90.0, 90.0	90.0, 90.0, 90.0
Resolution (Å)	118.84-2.11 (2.22-2.11) *	82.45-2.65 (2.79-2.65)
R_{sym}	0.10 (0.40)	0.28 (0.96)
$I / \sigma I$	13.6 (1.9)	3.0 (0.8)
Completeness (%)	99.5 (98.4)	94.9 (96.2)
Redundancy	8.1 (8.3)	2.9 (2.9)
**Refinement**		
Resolution (Å)	84.03-2.11	82.45-2.65
No. reflections	20746	34889
R_{work} / R_{free}	0.185 / 0.245	0.247 / 0.279
No. atoms		
Protein	2108	8916
Ligand (PON/PHI1)	39	164
Ligand (other)	49	13
Water	97	52
B-factors		
Protein	33.3	53.5
Ligand (PON/PHI1)	20.7	40.7
Ligand (other)	57.3	71.4
Water	35.6	
R.m.s. deviations		
Bond lengths (Å)	0.0185	0.011
Bond angles (°)	0.245	1.515

[a] Data from a single crystal.
*Values in parentheses are for highest-resolution shell.

Supplementary Table 2. List of kinases for which PHI1 (left) or Ponatinib (right) at 1 μM induced >35% displacement of control binding in KinomeEDGE scan.

Assay Label	% Ctrl
ABL1(E255K)-phosphorylated	0.25
ABL1(T315I)-phosphorylated	3.5
ABL1-nonphosphorylated	0
ABL1-phosphorylated	0.1
ACVR1B	0.4
BRAF	0.9
BRAF(V600E)	0.15
CDK11	0
CSF1R	0.1
EPHA	14
ERBB2	29
FLT3	3
IKK-alpha	30
JAK3(JH1domain-catalytic)	23
KIT	0
KIT(D816V)	11
KIT(V559D,T670I)	0.1
p38-beta	0.6
PDGFRA	0.5
PDGFRB	0
RAF1	6
RET	0.05
ROCK2	35
SRC	19
TIE2	16
TRKA	2
VEGFR2	0.2

Assay Label	% Ctrl
ABL1(E255K)-phosphorylated	0.1
ABL1(T315I)-phosphorylated	1
ABL1-nonphosphorylated	0
ABL1-phosphorylated	0.1
AXL	14
BRAF	0.6
BRAF(V600E)	0
BTK	22
CDK11	0.05
CDK7	6.2
CDK9	28
CSF1R	0.05
EGFR	15
EGFR(L858R)	19
EPHA2	0
ERBB2	0.3
ERBB4	27
FGFR1	0.15
FGFR3	0.9
FLT3	0
IKK-alpha	0.2
IKK-beta	1.4
JAK2(JH1domain-catalytic)	9.5
JAK3(JH1domain-catalytic)	0
JNK2	3.9
KIT	0
KIT(D816V)	2.6
KIT(V559D,T670I)	0.05
MKNK2	0.6
p38-alpha	0
p38-beta	0
PCTK1	32
PDGFRA	0.3
PDGFRB	0
PKAC-alpha	8.1
RAF1	11
RET	0
SRC	0
TIE2	0.05
TRKA	0.2
TYK2(JH1domain-catalytic)	8.6
VEGFR2	0.45

Supplementary Methods

Chemical Synthesis

All chemical reagents and solvents were obtained from commercial sources (Aldrich, Acros, Fisher) and used without further purification unless otherwise noted. Anhydrous solvents (tetrahydrofurane, toluene, dichloromethane, diethyl ether) were distilled in house before use. Chromatography was performed on a Biotage Isolera CombiFlash using silica gel column (200 mesh). Analytical thin layer chromatography (TLC) was performed on aluminum-backed Silicycle silica gel plates (250μm film thickness, indicator F254). Compounds were visualized using a dual wavelength (254 and 360 nm) UV lamp and/or staining with CAM (cerium ammonium molybdate) or $KMnO_4$ stains. NMR spectra were recorded on Bruker AVANCE III 300. ^{1}H and ^{13}C chemical shifts (δ) are reported relative to tetramethyl silane (TMS, 0.00/0.00 ppm) as internal standard or to residual solvent (CD_3OD: 3.31/49.00 ppm; $CDCl_3$: 7.26/77.16 ppm; dmso-d_6: 2.50/39.52 ppm). Mass spectra were recorded on a Shimadzu LCMS 2020.

Synthesis of PHI1-1 (N-[4-cyano-3-(trifluoromethyl)phenyl]-3-iodo-4-methylbenzamide)

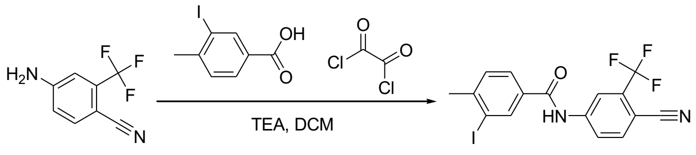

To a stirred solution of 3-iodo-4-methylbenzoic acid (28.156 g, 107.45 mmol, 1.000 equiv) and N,N-Dimethylformamide (1 drop) in dichloromethane (50 ml) was added oxalic dichloride (27.27 g, 214.85 mmol, 2.000 equiv) dropwise at 0°C. The resulting mixture was stirred at room temperature for 1 h. The resulting mixture was added to a solution of 4-amino-2-

(trifluoromethyl)benzonitrile (20 g, 107.45 mmol, 1 equiv) in dichloromethane (150 ml) at 0-5°C. And the mixture stirred at room temperature for 1 h. The reaction mixture was partitioned between dichloromethane and saturated aqueous sodium bicarbonate solution. The aqueous phase was separated and extracted with dichloromethane. The combined organic phases were washed with water, brine, dried over anhydrous sodium sulfate and concentrated under vacuum. This resulted in N-[4-cyano-3-(trifluoromethyl)phenyl]-3-iodo-4-methylbenzamide (16.1g,34.83%) as a white solid. LC-MS, PH1-2 (ES, m/z): $[M+1]^+$ = 431; RT = 1.58 min

Synthesis of PHI1-2 (N-[4-formyl-3-(trifluoromethyl)phenyl]-3-iodo-4-methylbenzamide)

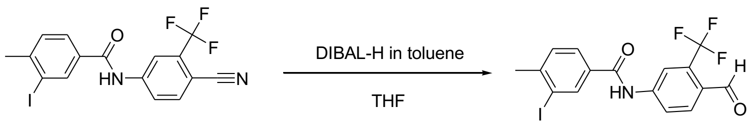

To a stirred solution of N-[4-cyano-3-(trifluoromethyl)phenyl]-3-iodo-4-methylbenzamide (8 g, 18.60 mmol, 1 equiv) in THF (240 mL, 2962.32 mmol, 159.285 equiv) was added DIBAl-H in toluene (93 mL, 93 mmol, 93 equiv) dropwise at 25-30°C under N_2 atmosphere. The resulting mixture was stirred at 25-30°C for 1.5 h. The reaction was quenched by the addition of 120 mL methanol and 120 mL of potassium sodium 2,3-dihydroxysuccinate solution at 25-30°C. The aqueous layer was extracted with tert-butyl methyl ether (3 x 250 mL). The resulting mixture was concentrated under vacuum. The crude product was purified by Prep-HPLC with the following conditions (Prep-HPLC-006): Column, XBridge Prep C18 OBD Column,, 5um,19*150mm; mobile phase, Water (10mmol/L NH_4HCO_3+0.1%$NH_3.H_2O$) and ACN (67% Phase B up to 71% in 6 min, hold 95% in 1 min, hold 67% in 1 min); Detector, UV. This resulted inN-[4-formyl-3-(trifluoromethyl)phenyl]-3-iodo-4-methylbenzamide (1.3g, 16.14%) as an off-white solid. LC-MS, PH1-3 (ES, m/z): $[M+1]^+$ = 434; RT = 1.70 min

Synthesis of PHI1-3 (N-[4-formyl-3-(trifluoromethyl)phenyl]-3-(2-[imidazo[1,2-b]pyridazin-3-yl]ethynyl)-4-methylbenzamide)

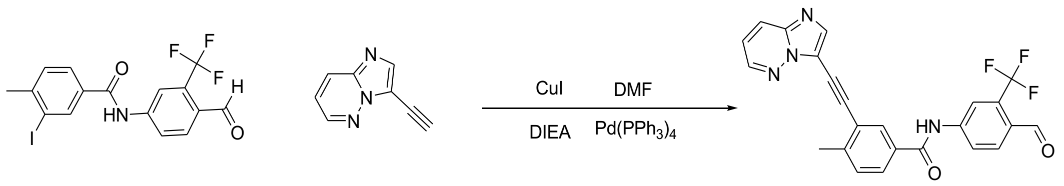

A mixture of N-[4-formyl-3-(trifluoromethyl)phenyl]-3-iodo-4-methylbenzamide (1.3 g, 3.00 mmol, 1 equiv), 3-ethynylimidazo[1,2-b] pyridazine(429mg, 3.00 mmol, 0.999 equiv), CuI(42.2 mg, 0.22 mmol, 0.074 equiv), $Pd(PPh_3)_4$(173 mg, 0.15 mmol, 0.050 equiv) and DIEA(0.8 mL, 4.84 mmol, 1.613 equiv) in DMF(20 mL, 258.44 mmol, 86.111 equiv) was stirred under N_2 atmosphere at room temperature overnight. The resulting mixture was diluted with ethyl acetate (50 mL). The resulting mixture was washed with 3x20 mL of water and 20 mL of brine. The resulting ethyl acetate phase was dried over anhydrous sodium sulfate and concentrated under vacuum.The residue was purified by silica gel column chromatography, eluted with EA/PE (0-50%) to afford N-[4-formyl-3-(trifluoromethyl)phenyl]-3-(2-[imidazo[1,2-b]pyridazin-3-yl]ethynyl)-4-methylbenzamide (1.0g,74.31%) as a yellow solid. LC-MS, PH1-3 (ES, m/z): $[M+1]^+ = 449$; RT = 1.61 min

Synthesis of PHI1 (N-[4-formyl-3-(trifluoromethyl)phenyl]-3-(2-[imidazo[1,2-b]pyridazin-3-yl]ethynyl)-4-methylbenzamide)

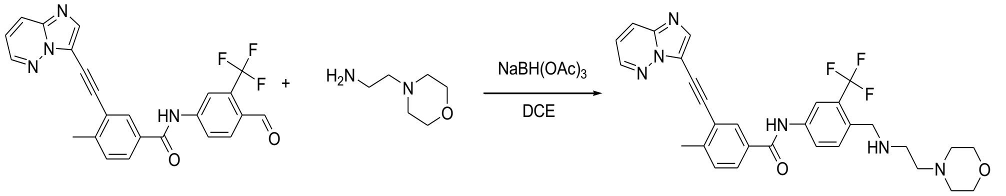

A mixture of N-[4-formyl-3-(trifluoromethyl)phenyl]-3-(2-[imidazo[1,2-b]pyridazin-3-yl]ethynyl)-4-methylbenzamide (50 mg, 0.11 mmol, 1 equiv) and 2-(morpholin-4-yl)ethan-1-amine (21.4 mg, 0.16 mmol, 1.474 equiv) in 1,2-dichloroethane (1 mL, 12.63 mmol, 113.278 equiv) was stirred at room temperature for 30 min. To a stirred mixture was added acetyl ethaneperoxoate sodioboranyl acetate (70 mg, 0.33 mmol, 2.962 equiv) in portions at room temperature. The resulting mixture was stirred under at room temperature overnight. The reaction was quenched with 0.2 mL water. The resulting mixture was concentrated under reduced pressure. The residue was purified by Prep-HPLC to afford 3-(2-[imidazo[1,2-b]pyridazin-3-yl]ethynyl)-4-methyl-N-[4-([[2-(morpholin-4-yl)ethyl]amino]methyl)-3 (trifluoromethyl)phenyl]benzamide (6.0 mg, 9.56%) as a white solid.

LC-MS: (ES, m/z): $[M+1]^+$ = 563.2; RT = 1.83 min. ^{1}H-NMR: (300 MHz, DMSO-d_6, *ppm*) δ 10.56 (s, 1H), 8.74 (d, *J* = 4.4 Hz, 1H), 8.25 (td, *J* = 10.5, 9.9, 3.1 Hz, 4H), 8.07 (d, *J* = 8.6 Hz, 1H), 8.01–7.91 (m, 1H), 7.73 (d, *J* = 8.6 Hz, 1H), 7.56 (d, *J* = 8.2 Hz, 1H), 7.40 (dd, *J* = 9.2, 4.5 Hz, 1H), 3.85 (s, 2H), 3.55 (t, *J* = 4.7 Hz, 4H), 2.62 (s, 5H), 2.41 (t, *J* = 6.2 Hz, 2H), 2.33 (s, 4H).

Synthesis of PHI2-1 tert-butyl N-[1-(6-ethylpyrimidin-4-yl)piperidin-4-yl]carbamate

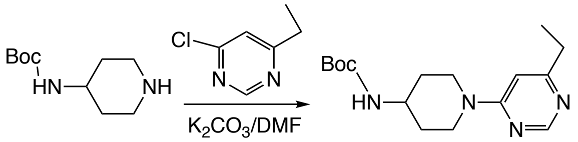

A solution/mixture of tert-butyl N-(piperidin-4-yl)carbamate (1.17 g, 5.85 mmol, 1.2 equiv), K_2CO_3 (1.35 g, 9.78 mmol, 2 equiv) and 4-chloro-6-ethylpyrimidine (700 mg, 4.89 mmol, 1 equiv) in DMF (20 mL, 258 mmol, 52.7 equiv) was stirred at 60°C for overnight. The resulting

mixture was diluted with H_2O (100 mL). The resulting mixture was filtered, the filter cake was washed with H_2O (2x20mL). The filtrate was concentrated under reduced pressure. This resulted in tert-butyl N-[1-(6-ethylpyrimidin-4-yl)piperidin-4-yl]carbamate (1.7 g, 96.06%) as a white solid. LC-MS (ES, m/z): $[M+1]^+$ = 307; RT = 1.11 min

Synthesis of PHI2-2 1-(6-ethylpyrimidin-4-yl)piperidin-4-amine hydrogen chloride

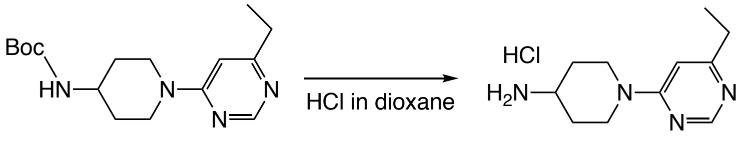

A mixture of tert-butyl N-[1-(6-ethylpyrimidin-4-yl)piperidin-4-yl]carbamate (1.7 g, 5.55 mmol, 1 equiv) and 4M HCl in dioxane (17 mL, 68 mmol, 12.25 equiv) was stirred at room temperature for 2 h. The resulting mixture was concentrated under reduced pressure. This resulted in 1-(6-ethylpyrimidin-4-yl)piperidin-4-amine hydrogen chloride (1.3 g, 96.51%) as a white solid. LC-MS (ES, m/z): $[M+1]^+$ = 207; RT = 0.36 min

Synthesis of PHI2 N-[4-([[1-(6-ethylpyrimidin-4-yl)piperidin-4-yl]amino]methyl)-3-(trifluoromethyl)phenyl]-3-(2-[imidazo[1,2-b]pyridazin-3-yl]ethynyl)-4-methylbenzamide

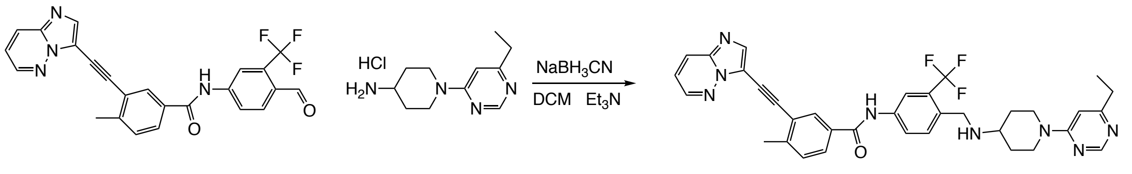

A solution of 1-(6-ethylpyrimidin-4-yl)piperidin-4-amine hydrochloride (62.8 mg, 0.30 mmol, 1.365 equiv) and Et_3N (55.6 mg, 0.55 mmol, 2.464 equiv) in DCM (2 mL, 31.46 mmol, 141.066 equiv) was stirred at room temperature for 5 min. N-[4-formyl-3-(trifluoromethyl)phenyl]-3-

(2-[imidazo[1,2-b]pyridazin-3-yl]ethynyl)-4-methylbenzamide (100 mg, 0.22 mmol, 1 equiv) was added with stirring at room temperature. The resulting mixture was stirred at room temperature for 30 min. Sodium carbonitrile borane (41.5 mg, 0.66 mmol, 2.961 equiv) was added in portions with stirring at room temperature. The resulting mixture was stirred at room temperature for 2 h. The reaction was quenched with 1 mL water. The resulting mixture was concentrated under vacuum. The crude product was purified by Prep-HPLC to afford N-[4-([[1-(6-ethylpyrimidin-4-yl)piperidin-4-yl]amino]methyl)-3-(trifluoromethyl)phenyl]-3-(2-[imidazo[1,2-b]pyridazin-3-yl]ethynyl)-4-methylbenzamide (16.1mg,11.30%) as a white solid. LC-MS (ES, m/z): $[M+1]^+$ = 639; RT =3.35 min.

^{1}H-NMR (300 MHz, DMSO-d_6, *ppm*) δ 10.55 (s, 1H), 8.74 (dd, *J* = 4.5, 1.6 Hz, 1H), 8.39 (s, 1H), 8.33–8.18 (m, 4H), 8.07 (d, *J* = 8.2 Hz, 1H), 8.01–7.92 (m, 1H), 7.81 (d, *J* = 8.5 Hz, 1H), 7.57 (d, *J* = 8.1 Hz, 1H), 7.41 (dd, *J* = 9.2, 4.5 Hz, 1H), 6.68 (s, 1H), 4.25 (d, *J* = 13.5 Hz, 2H), 3.90 (s, 2H), 3.02 (s, 2H), 2.62 (s, 3H), 2.56 (s, 1H), 2.20 (s, 1H), 1.89 (d, *J* = 12.7 Hz, 2H), 1.34–1.12 (m, 5H).
