# Supplementary material for: Inhibitors of BRAF dimers using an allosteric site
Source: Nat Commun. 2020 Sep 1;11:4370. doi: 10.1038/s41467-020-18123-2 (PMC7462985; doi:10.1038/s41467-020-18123-2)
Supplement: Supplementary file 2 — Reporting Summary [file 41467_2020_18123_MOESM2_ESM.pdf]

nature research

Corresponding author(s): Evripidis Gavathiotis, Bogos Agianian

Last updated by author(s): Jul 30, 2020

Reporting Summary

Nature Research wishes to improve the reproducibility of the work that we publish. This form provides structure for consistency and transparency in reporting. For further information on Nature Research policies, see Authors & Referees and the Editorial Policy Checklist .

Statistics

For all statistical analyses, confirm that the following items are present in the figure legend, table legend, main text, or Methods section.

n/a	Confirmed	
☐	☒	The exact sample size (*n*) for each experimental group/condition, given as a discrete number and unit of measurement
☐	☒	A statement on whether measurements were taken from distinct samples or whether the same sample was measured repeatedly
☐	☒	The statistical test(s) used AND whether they are one- or two-sided<br>*Only common tests should be described solely by name; describe more complex techniques in the Methods section.*
☐	☒	A description of all covariates tested
☐	☒	A description of any assumptions or corrections, such as tests of normality and adjustment for multiple comparisons
☐	☒	A full description of the statistical parameters including central tendency (e.g. means) or other basic estimates (e.g. regression coefficient) AND variation (e.g. standard deviation) or associated estimates of uncertainty (e.g. confidence intervals)
☒	☐	For null hypothesis testing, the test statistic (e.g. *F*, *t*, *r*) with confidence intervals, effect sizes, degrees of freedom and *P* value noted<br>*Give P values as exact values whenever suitable.*
☒	☐	For Bayesian analysis, information on the choice of priors and Markov chain Monte Carlo settings
☒	☐	For hierarchical and complex designs, identification of the appropriate level for tests and full reporting of outcomes
☒	☐	Estimates of effect sizes (e.g. Cohen's *d*, Pearson's *r*), indicating how they were calculated

Our web collection on statistics for biologists contains articles on many of the points above.

Software and code

Policy information about availability of computer code

Data collection	Modeling data were collected and analyzed with Schrodinger software suite (Releases 2016-2018, Schrödinger, LLC) and Pymol (Version 2.3, The PyMOL Molecular Graphics System, Schrödinger, LLC). Crystallographic data were processed with iMosflm (Version 7.2.1). Structures were solved and refined using CCP4 suite (Version 7.0) and REFMAC (Version 5.8.0158). Cell colony sizes were analyzed using ImageJ (Version 1.51).
Data analysis	Data analysis and statistical comparisons were performed by Graphpad Prism 7.0 and 8.0 software. Structural and small molecule data were analyzed with modules SiteMap, GLIDE, EPIC, LIGPREP, MAESTRO of Schrodinger software suite (Releaseses 2016-2018). Western blot data were analyzed with Image Studio (Version 3.1, LICOR).

For manuscripts utilizing custom algorithms or software that are central to the research but not yet described in published literature, software must be made available to editors/reviewers. We strongly encourage code deposition in a community repository (e.g. GitHub). See the Nature Research guidelines for submitting code & software for further information.

Data

Policy information about availability of data

All manuscripts must include a data availability statement. This statement should provide the following information, where applicable:

- Accession codes, unique identifiers, or web links for publicly available datasets
- A list of figures that have associated raw data
- A description of any restrictions on data availability

Source data related to this work are provided. Data generated or analyzed during this study and included in this article are available from the corresponding authors on a reasonable request. BRAFV600E/Ponatinib and BRAFV600E/PHI1 structure coordinates have been deposited with the PDB with accession codes 6P3D [http://dx.doi.org/10.2210/pdb6p3d/pdb] and 6P7G [http://dx.doi.org/10.2210/pdb6p7g/pdb], respectively. Other PDB codes: 4RZW [http://dx.doi.org/10.2210/pdb4rzw/pdb], 5C9C [http://dx.doi.org/10.2210/pdb5c9c/pdb], 4KSP [http://dx.doi.org/10.2210/pdb4ksp/pdb], 4G9R [http://dx.doi.org/10.2210/pdb4g9r/pdb], 3OG7 [http://dx.doi.org/10.2210/pdb3og7/pdb], 4XV2 [http://dx.doi.org/10.2210/pdb4xv2/pdb], 4MNE [http://dx.doi.org/10.2210/pdb4mne/pdb].

Field-specific reporting

Please select the one below that is the best fit for your research. If you are not sure, read the appropriate sections before making your selection.

- [x] Life sciences
- [ ] Behavioural & social sciences
- [ ] Ecological, evolutionary & environmental sciences

For a reference copy of the document with all sections, see nature.com/documents/nr-reporting-summary-flat.pdf

Life sciences study design

All studies must disclose on these points even when the disclosure is negative.

Sample size	Sample sizes were designed based on our previous publication (Karoulia et al. Cancer Cell, 2016, 30:485-498, [doi: 10.1016/j.ccell.2016.06.024]), with assays on related projects and variability of the response deviating from the mean as presented in the graphs and figure legends. Sample sizes and statistical data are reported in figure legends.
Data exclusions	No data were excluded from analyses.
Replication	Experimental findings were reproduced in all attempts.
Randomization	Allocation was random.
Blinding	Blinding was used for 3D-colony formation assay because data analysis was automated. For western blot analyses blinding was not used to ensure quality control of small molecules in manual control experiments. No animal/human subjects were used to bias group selection.

Reporting for specific materials, systems and methods

We require information from authors about some types of materials, experimental systems and methods used in many studies. Here, indicate whether each material, system or method listed is relevant to your study. If you are not sure if a list item applies to your research, read the appropriate section before selecting a response.

Materials & experimental systems

n/a	Involved in the study	
[ ]	[x]	Antibodies
[ ]	[x]	Eukaryotic cell lines
[x]	[ ]	Palaeontology
[x]	[ ]	Animals and other organisms
[x]	[ ]	Human research participants
[x]	[ ]	Clinical data

Methods

n/a	Involved in the study	
[x]	[ ]	ChIP-seq
[x]	[ ]	Flow cytometry
[x]	[ ]	MRI-based neuroimaging

Antibodies

Antibodies used

BRAF (Santa Cruz, sc-5284). Dilution 1:1000
BRAFV600E (NewEast BioSciences, 26039). Dilution 1:1000
CRAF (Santa Cruz, sc-133). Dilution 1:1000
MEK1 (Millipore, 07-641). Dilution 1:1000
MEK1/2 (Cell Signaling, 4694). Dilution 1:1000
P-MEK1/2 (Cell Signaling, 9154). Dilution 1:1000
ERK1/2 (Cell Signaling, 4696). Dilution 1:1000
P-ERK (Santa Cruz, sc-7383). Dilution 1:200
P-ERK1/2 (Cell Signaling, 4370). Dilution 1:500
ERK1 (Santa Cruz, sc-94). Dilution 1:200
Actin (Invitrogen, MA5-15739). Dilution 1:10000
GAPDH (Sigma, G8795). Dilution 1:5000
IRDye800CW (LICOR, 926-32210). Dilution 1:800
IRDye680RD (LICOR, 926-68071). Dilution 1:800

Validation

All antibodies used in the study have been validated in the literature and such information is provided in the manufacturer's website. Pubmed IDs describing example publications are given below:
BRAF (Santa Cruz, sc-5284). Dilution 1:1000. Example publication Pubmed ID: 31182717
BRAFV600E (NewEast BioSciences, 26039). Dilution 1:1000. Example publication Pubmed ID: 23041829
CRAF (Santa Cruz, sc-133). Dilution 1:1000. Example publication Pubmed ID: 26657898

MEK1 (Millipore, 07-641). Dilution 1:1000. Example publication Pubmed ID: 17974567
MEK1/2 (Cell Signaling, 4694). Dilution 1:1000. Example publication Pubmed ID: 31958057
P-MEK1/2 (Cell Signaling, 9154). Dilution 1:1000. Example publication Pubmed ID: 7911739
ERK1/2 (Cell Signaling, 4696). Dilution 1:1000. Example publication Pubmed ID: 32463582
P-ERK (Santa Cruz, sc-7383). Dilution 1:200. Example publication Pubmed ID: 8929541
P-ERK1/2 (Cell Signaling, 4370). Dilution 1:500. Example publication Pubmed ID: 32377705
ERK1 (Santa Cruz, sc-94). Dilution 1:200. Example publication Pubmed ID: 26876200
Actin (Invitrogen, MA5-15739). Dilution 1:10000. Example publication Pubmed ID: 30275445
GAPDH (Sigma, G8795). Dilution 1:5000. Example publication Pubmed ID: 12887926
IRDye800CW (LICOR, 926-32210). Dilution 1:800. Example publication Pubmed ID: 32007473
IRDye680RD (LICOR, 926-68071). Dilution 1:800. Example publication Pubmed ID: 25344226

Eukaryotic cell lines

Policy information about cell lines

Cell line source(s)	Cells were purchased from ATCC and also obtained from Poulikos Poulikakos laboratory. Cell lines used: A375, SKMEL239, SKMEL239-C4, SKMEL-30, SKMEL-2, HEK239H, CALU6, H1666, H2087.
Authentication	Cell lines were authenticated from their vendor. ATCC uses morphology, karyotyping, and STR profiling to confirm the identity of human cell lines and to rule out both intra- and interspecies contamination.
Mycoplasma contamination	All cell lines were tested negative for mycoplasma contamination.
Commonly misidentified lines (See ICLAC register)	No commonly misidentified lines used in our study.
