# Supplementary material for: Inhibitors of BRAF dimers using an allosteric site
Source: Nat Commun. 2020 Sep 1;11:4370. doi: 10.1038/s41467-020-18123-2 (PMC7462985; doi:10.1038/s41467-020-18123-2)
Supplement: Supplementary file 3 — Peer Review [file 41467_2020_18123_MOESM3_ESM.pdf]

Reviewers' comments:

Reviewer #1 (Remarks to the Author):

The current work reports a newly identified BRAF inhibitor that binds to BRAF dimers using a novel binding interaction which stabilizes a distinct alpha C helix conformation. This unique feature makes this inhibitor more potently inhibit BRAFV600E dimers, in comparison with vemurafenib. More intriguingly, the authors claim that this inhibitor displays positive cooperativity instead of negative cooperativity that is notoriously known as one major cause of drug resistance for current BRAF therapies, like vemurafenib. However, there are numbers of suggestions and comments that should be addressed before the manuscript is considered for publication:

(1) It is commonly believed that purified apo-BRAFV600E kinase domain is dimeric in solution ("Chemically Linked Vemurafenib Inhibitors Promote an Inactive BRAFV600E Conformation" by Grasso et al) while ATP analog AMP-PNP is known to disrupt BRAF dimers in solution ("Inhibitors that stabilize a closed RAF kinase domain conformation induce dimerization" by Lavoie et al). I would like to see more evidence supporting that PON and PHI1 stabilize BRAFV600E dimers in vitro. At least, the oligomeric status of apo-BRAFV600E and ligand-bound BRAFV600E should be compared.

(2) PON and PHI1 display similar potency against BRAFV600E in kinases assays (IC50 values are very close, 14nM vs.10 nM), however, distinct potency was observed in A375 cell-based assays (Fig 6a&b). It will be helpful to elaborate this discrepancy.

(3) PON behaves more like any pan-RAF inhibitor, with equal potency toward monomers and dimers. In contrary, PHI1 more preferentially inhibits dimers. Apparently, PHI1 has different inhibition mechanism, most likely due to the allosteric binding site, thus the conclusions drawn from PON data should not be extrapolated to PHI1 without taking extra caution. More cell-based assays are necessary to evaluate the cellular activity of PHI1, in comparison with type I and type II inhibitors, to validate its potential as novel BRAF therapy. Additionally, it is interesting to check whether PHI1 eliminates paradoxical activation of wild-type BRAF in cells carrying oncogenic RAS mutations.

(4) Like all other RAF inhibitors, PHI1 inhibits the kinase activity of BRAF by occupying the ATP-binding pocket. The allosteric site might facilitate the selectivity toward dimers. The title is a bit of misleading. It seems that PHI1 is an allosteric inhibitor.

(5) Since PHI1 is more selective for BRAFV600E dimers (Fig 6), a R509H mutation is a good control to further strengthen this conclusion. Similarly, a R509H mutation experiment is recommended for figure 7a-d.

(6) The synergy results (Fig 6) obtained from PHI1 and Enco can be explained by another possibility that pre-treatment with Enco eliminates the cellular activity of BRAFV600E monomers and PHI1 inhibits p61 BRAFV600E dimers, not necessary because of positive cooperativity.

Reviewer #2 (Remarks to the Author):

Summary: Mutant selective BRAF inhibitors have revolutionized treatment of metastatic melanoma. Unfortunately, these inhibitors are limited by divergent mechanisms of resistance. As described by Cotto-Rios et al., RAF inhibitors can be characterized by their ability to stabilize a α-C helix in or α-C helix out confirmation, which makes them more or less effective in inhibiting monomeric and/or dimeric RAF, respectively. Paradoxically, both sets of RAF inhibitors either induce negative allostery or RAF priming, which function as intrinsic mechanism of resistance related to the inhibitor binding properties. Therefore, Cotto-Rios et al. set out to identify novel RAF dimer inhibitors to be utilized in

the context of BRAFV600E resistant tumors and wild type RAF-dependent tumors. Based on the ability of p61BRAFV600E splice variant to function in a RAS-independent but dimerization dependent fashion, the authors leveraged a cell harboring this alteration to screen for novel BRAFV600E dimer inhibitors. The authors identified the pan tyrosine kinase inhibitor Ponatinib as a potent inhibitor of mutant BRAFV600E over wild-type BRAF with a similar IC50 towards its known targets, like ABL, FLT3, KIT, and PDGFR. By solving the X-ray crystal structure of Ponatinib bound to BRAF, the authors demonstrated the Ponatinib uniquely stabilizes a α-C helix centre conformation that in turn stabilizes RAF dimers and MEK interactions. In cells with different BRAF conformation and states, Ponatinib reduced MAPK signaling and tumorigenic properties. Finally, the authors improve upon Ponatinib by developing PHI1, which is selective for BRAF dimers in an α-C helix in confirmation. Overall, these studies identify that Ponatinib can be repurposed in the context of BRAF-dimer dependent cancers and a novel allosteric site within BRAF. Despite the elegant chemical biology and structural work in the manuscript, one major question remains about the extent to which Ponatinib is targeting BRAF in cells to impact reduce cancer cell growth. Therefore, the authors should attempt to decipher these distinct modes of action especially since the cellular experiments are conducted at concentrations that would inhibit all of the targets.

Major points:
1. Given that several of the additional targets of Ponatinib are known resistance mechanisms to Vemurafenib, the authors should test the extent to which Ponatinib targeting of p61BRAFV600E contributes to the reduced activation of the MAPK pathway in SKMEL-239 C4 cells by either knocking out p61BRAFV600E and showing no further reduction in p-ERK1/2 or overexpressing p61BRAFV600E in a non-transformed cell line and determining the extent to which p-ERK1/2 is reduced and tumorigenic properties are reduced.

Minor points:
1. In the abstract, the description of PHI1 exhibiting cellular selectivity for BRAFV600E dimers is misleading unless this is in the context of resistance.
2. One line 59 simultaneous is spelled wrong.
3. On line 103 I think it’s referring to protomer and not promoter.

Reviewer #3 (Remarks to the Author):

The authors identified ponatinib, an FDA-approved drug targeting BCR-ABL in CML, as a potent inhibitor of monomeric and dimeric BRAF V600E offering alternative targeting opportunities in TKI-resistant melanoma and other BRAF-driven diseases. Analysis of crystal structures identified a novel allosteric pocket formed by a particular (and not previously observed) conformation of the alpha C helix. In order to more effectively exploit this allosteric site, structure-guided drug design identified PHI1. Structural analysis and biochemical assays show that PHI1 represents a novel class of dimer-selective inhibitors.

This is an impressive piece of work that validates BRAF as a biologically valid off-target of the BCR-ABL gatekeeper mutant inhibitor ponatinib. In addition, careful structural biology and drug discovery efforts establish a new binding mode for pontatinib and PHI. Together with the detailed validation experiments, the authors convincingly demonstrate a novel targeting strategy. All experiments are amply controlled and deliver unequivocal results, the manuscript is well written and easy to follow. Crystallography and compound development is state-of-the-art. A possible second medical use of ponatinib and further development of PHI1 as a melanoma drug are important implication that should be reported and will be of interest for a broad readership beyond the BRAF scientific community.

Minor points:

1. Given the complexities of RAF regulation and targeting, a schematic representation (such as the one in SI Fig. 1B) illustrating BRAF regulation and the screening assay principle should be added to Fig 1. This will help the broad readership of Nat. Comm that are not RAF regulation aficionados.

2. SI Fig. 2: The legend (or figure labels) should list at what concentrations the kinase inhibitors from the in cell Western screen were used for these experiments.

3. It would be interesting if the authors could speculate, based on the known plasma levels of ponatinib in CML patients, on the relevance of BRAF inhibition in CML patients that are treated with ponatinib. Are there any of the observed adverse events to ponatinib that might point towards a major BRAF involvement?

4. The PDB entry codes for the two crystal structures that are reported in the manuscript should be added to Data availability statement and the SI Table 1.

We would like to thank the reviewers for their careful review of our work. We have provided additional experimental data that provide further insights and strengthen our conclusions. We also provided changes in the text to further clarify our results. We greatly appreciate your important contribution to improve our manuscript.

To facilitate the review process we indicated in each answer the figure panels or pages where the new results and text are displayed in the revised manuscript. Changes that address the referee's comments in the text are marked in blue fonts.

We hope that with the additional experimental data and revision of the manuscript, the reviewers will find the revised manuscript suitable for publication.

Reviewers' comments:

Reviewer #1 (Remarks to the Author):

The current work reports a newly identified BRAF inhibitor that binds to BRAF dimers using a novel binding interaction which stabilizes a distinct alpha C helix conformation. This unique feature makes this inhibitor more potently inhibit BRAFV600E dimers, in comparison with vemurafenib. More intriguingly, the authors claim that this inhibitor displays positive cooperativity instead of negative cooperativity that is notoriously known as one major cause of drug resistance for current BRAF therapies, like vemurafenib. However, there are numbers of suggestions and comments that should be addressed before the manuscript is considered for publication:

We thank the reviewer for the positive remarks of our work and experimental suggestions to strengthen our study. We hope with the additional experiments we have addressed the remaining points.

(1) It is commonly believed that purified apo-BRAFV600E kinase domain is dimeric in solution ("Chemically Linked Vemurafenib Inhibitors Promote an Inactive BRAFV600E Conformation" by Grasso et al) while ATP analog AMP-PNP is known to disrupt BRAF dimers in solution ("Inhibitors that stabilize a closed RAF kinase domain conformation induce dimerization" by Lavoie et al). I would like to see more evidence supporting that PON and PHI1 stabilize BRAFV600E dimers in vitro. At least, the oligomeric status of apo-BRAFV600E and ligand-bound BRAFV600E should be compared.

To address this point, we have performed additional size-exclusion chromatography analysis of apo-BRAFV600E kinase domain and ATP bound BRAFV600E kinase domain along with the PON and PHI1 complexes of BRAFV600E kinase domain, in Supplementary Fig. 9. Our chromatographic analysis shows that freshly prepared and analyzed apo-BRAFV600E kinase domain predominantly is monomeric although the dimeric and oligomeric fractions are also evident in solution. This profile of apo-BRAFV600E is in agreement with observations by Lavoie et al. (Figure 5e) who showed using AUC that apo-BRAF is monomer at low concentrations in equilibrium with dimer species that increase upon concentration. However, apo-BRAFV600E is an unstable protein and highly amenable to oligomerization, and eventually aggregation upon time, therefore, differences in apo-BRAF chromatographic profiles are plausible, and will depend

on material treatment and experimental conditions. In contrast, ATP bound BRAFV600E kinase domain runs as a stable and distinct monomeric conformation. Both PON and PHI complexes clearly run as stable dimeric species with overlapping peaks in the chromatogram (Supplementary Fig. 9).

(2) PON and PHI1 display similar potency against BRAFV600E in kinases assays (IC50 values are very close, 14nM vs.10 nM), however, distinct potency was observed in A375 cell-based assays (Fig 6a&b). It will be helpful to elaborate this discrepancy.

We agree with the reviewer about the limitation of the *in vitro* kinase assay with BRAFV600E that is not able to fully recapitulate the distinct and complex structural and functional BRAF regulation in cellular context and the differences observed with PON and PHI1 specifically in A375 cell-based assays (Fig. 6). To elaborate on this discrepancy we have added a discussion about this point on page 20.

(3) PON behaves more like any pan-RAF inhibitor, with equal potency toward monomers and dimers. In contrary, PHI1 more preferentially inhibits dimers. Apparently, PHI1 has different inhibition mechanism, most likely due to the allosteric binding site, thus the conclusions drawn from PON data should not be extrapolated to PHI1 without taking extra caution. More cell-based assays are necessary to evaluate the cellular activity of PHI1, in comparison with type I and type II inhibitors, to validate its potential as novel BRAF therapy. Additionally, it is interesting to check whether PHI1 eliminates paradoxical activation of wild-type BRAF in cells carrying oncogenic RAS mutations.

To evaluate whether PHI1 has a unique cellular activity, in comparison with type I and type II RAF inhibitors, as we previously demonstrated with p61BRAFV600E dimers in SKMEL239 C4 cells (Figure 6), we used additional cell lines that express dimeric BRAF mutants of class II and class III as well as cell lines expressing RAS mutants with wildtype BRAF (new figure 7). We performed treatments of four cell lines with PH1 and representative type I (Vemurafenib) and type II (TAK-632) inhibitors without and with Encorafenib pre-treatment (and wash out) to evaluate the positive co-operativity effect. The results suggest that PHI1 demonstrates positive co-operativity upon pre-treatment with Encorafenib, in H2087 ($BRAF^{L597V}$, class II) and H1666 ($BRAF^{G466V}$, class III) cells, whereas TAK-632 has equipotent inhibition of BRAF mutants with or without Encorafenib pre-treatment, and Vemurafenib is impacted by considerable negative co-operativity and paradoxical activation in these cell lines expressing BRAF dimers. Interestingly, these experiments also showed that PHI1 has paradoxical activation at various degrees in these cell lines presumably because of RAF priming in the presence of active RAS or low occupancy of the first protomer. Therefore, similar to type I (Vemurafenib) and type II (TAK-632) inhibitors, PHI1 has paradoxical activation as a single agent, however, with Encorafenib pre-treatment in cells with either non-V600E (Figure 7) or V600E (Figure 6) BRAF, PHI1 shows significant inhibition activity of ERK signaling without paradoxical activation. These results corroborate our finding using SKMEL239 C4 cell line ($p61BRAF^{V600E}$) and further support the mechanism of positive co-operativity as a unique property of PHI1 inhibitor compared to other BRAF inhibitors. We described these results in pages 17 and 18 and also provide related discussion in page 21.

(4) Like all other RAF inhibitors, PHI1 inhibits the kinase activity of BRAF by occupying the ATP-binding pocket. The allosteric site might facilitate the selectivity toward dimers. The title is a bit of misleading. It seems that PHI1 is an allosteric inhibitor.

We can see the point of the reviewer but we feel that the discovery of the allosteric site is an important discovery associated with PHI1 and PON and the title refers to the inhibitors “using” the allosteric site but not being exclusively allosteric inhibitors.

Regarding the use of the term “allosteric site”, we simply followed standard nomenclature in many publications describing kinase inhibitors that bind to similar sites next to the ATP-binding pocket (please see examples how others name similar sites as allosteric in references Zhang et al. Nature Rev. Cancer 2009, Wu et al, Trends in Pharmacological Sciences 2015, Fabbro et al British Journal of Pharmacology 2015. Moreover, the use of “allosteric” is in agreement with the nomenclature used in the KLIFS database http://klifs.vu-compmedchem.nl/ for kinase inhibitor binding modes and allosteric pockets. We would like to mention that the allosteric site of PHI1 is not present in other kinase structures when they were searched using the KLIFS database.

(5) Since PHI1 is more selective for BRAFV600E dimers (Fig 6), a R509H mutation is a good control to further strengthen this conclusion. Similarly, a R509H mutation experiment is recommended for figure 7a-d.

We agree with the reviewer’s suggestion and we tested whether PHI1 is more selective for BRAFV600E dimers compared to monomers using the R509H mutation. We used non-transformed HEK293 cells and transiently overexpressed p61BRAFV600E and p61BRAFV600ER509H mutants (Supplementary Fig. 17). We found that PHI1 again is more effective in inhibiting p-ERK1/2 signaling upon expression of p61BRAFV600E compared to inhibition of p-ERK1/2 signaling upon expression of BRAFV600ER509H mutant. These results are consistent with the selective inhibition of PHI1 in SKMEL-239 C4 expressing p61BRAFV600E dimers compared to SKMEL-239 or A375 cells expressing BRAFV600E monomers (Figure 6, Supplementary Fig. 16). We also considered the suggestion to perform a target engagement CETSA assay as in the current Supplemental Fig. 22 (previously Figure 7a-d) by including the R509H mutation, but it has been technically challenging to incorporate R509H mutation in SKMEL-239 C4 cells with endogenous BRAF. In addition, the CETSA conditions require saturating concentrations of the inhibitors. We therefore feel that such experiment would not yield conclusive result in terms of the selective inhibition effect by PHI1, which is however addressed in Supplementary Fig. 17.

(6) The synergy results (Fig 6) obtained from PHI1 and Enco can be explained by another possibility that pre-treatment with Enco eliminates the cellular activity of BRAFV600E monomers and PHI1 inhibits p61 BRAFV600E dimers, not necessary because of positive cooperativity.

Conceptually, this could be an alternative explanation to interpret the results of PHI1 inhibition of p61BRAFV600E dimers compared to BRAFV600E monomers and why we observe positive co-operativity with PHI1. However, SKMEL-239 C4 cells are generated and kept upon Vemurefenib resistance pressure and should have minimal amounts, if any, of BRAFV600E monomers. To address this experimentally, we have analyzed SKMEL-239 C4 cells in the presence or absence of Vemurafenib and subjected them to size exclusion chromatography (Supplemental Fig. 21). We evaluated the presence and size of BRAFV600E species by fractionation and western blot analysis (Supplemental Fig. 21). Both analyses showed that p61BRAFV600E species run as dimers, based on the expected molecular weight of the protein and the analysis does not support

the presence of BRAFV600E monomers. Based on these data, and new data in figure 7 with PHI1 and cell lines expressing non-V600E BRAF mutants, we believe the mechanism for positive co-operativity is further supported.

Reviewer #2 (Remarks to the Author):

Summary: Mutant selective BRAF inhibitors have revolutionized treatment of metastatic melanoma. Unfortunately, these inhibitors are limited by divergent mechanisms of resistance. As described by Cotto-Rios et al., RAF inhibitors can be characterized by their ability to stabilize a α-C helix in or α-C helix out confirmation, which makes them more or less effective in inhibiting monomeric and/or dimeric RAF, respectively. Paradoxically, both sets of RAF inhibitors either induce negative allostery or RAF priming, which function as intrinsic mechanism of resistance related to the inhibitor binding properties. Therefore, Cotto-Rios et al. set out to identify novel RAF dimer inhibitors to be utilized in the context of BRAFV600E resistant tumors and wild type RAF-dependent tumors. Based on the ability of p61BRAFV600E splice variant to function in a RAS-independent but dimerization dependent fashion, the authors leveraged a cell harboring this alteration to screen for novel
BRAFV600E dimer inhibitors. The authors identified the pan tyrosine kinase inhibitor Ponatinib as a potent inhibitor of mutant BRAFV600E over wild-type BRAF with a similar IC50 towards its known targets, like ABL, FLT3, KIT, and PDGFR. By solving the X-ray crystal structure of Ponatinib bound to BRAF, the authors demonstrated the Ponatinib uniquely stabilizes a α-C helix centre conformation that in turn stabilizes RAF dimers and MEK interactions. In cells with different BRAF conformation and states, Ponatinib reduced MAPK signaling and tumorigenic properties. Finally, the authors improve upon Ponatinib by developing PHI1, which is selective for BRAF dimers in an α-C helix in confirmation. Overall, these studies identify that Ponatinib can be repurposed in the context of BRAF-dimer dependent cancers and a novel allosteric site within BRAF. Despite the elegant chemical biology and structural work in the manuscript, one major question remains about the extent to which Ponatinib is targeting BRAF in cells to impact reduce cancer cell growth. Therefore, the authors should attempt to decipher these distinct modes of action especially since the cellular experiments are conducted at concentrations that would inhibit all of the targets.

We really appreciate the reviewer's careful analysis of our work and positive comments about our findings and we hope we have addressed the remaining points.

Major points:
1. Given that several of the additional targets of Ponatinib are known resistance mechanisms to Vemurafenib, the authors should test the extent to which Ponatinib targeting of p61BRAFV600E contributes to the reduced activation of the MAPK pathway in SKMEL-239 C4 cells by either knocking out p61BRAFV600E and showing no further reduction in p-ERK1/2 or overexpressing p61BRAFV600E in a non-transformed cell line and determining the extent to which p-ERK1/2 is reduced and tumorigenic properties are reduced.

We agree with the reviewer's concern and we decided to test directly whether Ponatinib targets p61BRAFV600E in a non-transformed cell line to reduce the extent of ERK1/2 signaling pathway activation. In Supplementary Fig. 10 we show that overexpression of p61BRAFV600E in HEK293 cells increased p-ERK1/2 while treatment of the same cells that overexpress p61BRAFV600E

with Ponatinib resulted in p-ERK1/2 inhibition in a dose-dependent manner. In this overexpression system the higher IC50 of p-ERK1/2 inhibition by Ponatinib compared to the IC50 with endogenous p61BRAFV600E in SKMEL-239 C4 is expected. Therefore, these results, in addition to results in Fig. 4b and Supplementary Fig.22, further demonstrate that Ponatinib is capable of directly targeting BRAF and inhibiting it in cells irrespective of other targets.

Minor points:
1. In the abstract, the description of PHI1 exhibiting cellular selectivity for BRAFV600E dimers is misleading unless this is in the context of resistance.

We agree with the reviewer. However, given that in additional experiments we also found activity by PHI1 on other BRAF mutants that form dimers (class II and III), we revised this description in the abstract as "*PHI1 exhibits discrete cellular selectivity for BRAF dimers, with enhanced inhibition of the second protomer when the first protomer is occupied..*"

We hope the reviewer find this suitable to describe our overall findings.

2. One line 59 simultaneous is spelled wrong.

We corrected this.

3. On line 103 I think it's referring to protomer and not promoter.

We corrected this.

Reviewer #3 (Remarks to the Author):

The authors identified ponatinib, an FDA-approved drug targeting BCR-ABL in CML, as a potent inhibitor of monomeric and dimeric BRAF V600E offering alternative targeting opportunities in TKI-resistant melanoma and other BRAF-driven diseases. Analysis of crystal structures identified a novel allosteric pocket formed by a particular (and not previously observed) conformation of the alpha C helix. In order to more effectively exploit this allosteric site, structure-guided drug design identified PHI1. Structural analysis and biochemical assays show that PHI1 represents a novel class of dimer-selective inhibitors.

This is an impressive piece of work that validates BRAF as a biologically valid off-target of the BCR-ABL gatekeeper mutant inhibitor ponatinib. In addition, careful structural biology and drug discovery efforts establish a new binding mode for pontatinib and PHI. Together with the detailed validation experiments, the authors convincingly demonstrate a novel targeting strategy. All experiments are amply controlled and deliver unequivocal results, the manuscript is well written and easy to follow. Crystallography and compound development is state-of-the-art. A possible second medical use of ponatinib and further development of PHI1 as a melanoma drug are important implication that should be reported and will be of interest for a broad readership beyond the BRAF scientific community.

We really appreciate the reviewer's thoughtful analysis and positive remarks about our findings. We fully agree with the broader interest and implications of this work and we hope we have addressed the remaining suggestions.

Minor points:

1. Given the complexities of RAF regulation and targeting, a schematic representation (such as the one in SI Fig. 1B) illustrating BRAF regulation and the screening assay principle should be added to Fig 1. This will help the broad readership of Nat. Comm that are not RAF regulation aficionados.

That's a great suggestion. We have provided two schematics in the first figure (Figure 1a and 1b) to illustrate the ERK signaling of different BRAF species referred in the manuscript and the screening assay.

2. SI Fig. 2: The legend (or figure labels) should list at what concentrations the kinase inhibitors from the in cell Western screen were used for these experiments.

We now provide concentrations of all kinase inhibitors used in the experiments in the figure legend of SI Fig2.

3. It would be interesting if the authors could speculate, based on the known plasma levels of ponatinib in CML patients, on the relevance of BRAF inhibition in CML patients that are treated with ponatinib. Are there any of the observed adverse events to ponatinib that might point towards a major BRAF involvement?

This is an interesting suggestion. To our knowledge, the clinical implications of BRAF targeting by Ponatinib have not been investigated and our work provides the mechanistic framework to initiate such clinical studies. We speculated on the potential benefit of CML patients with additional BRAF inhibition by Ponatinib. On page 19, we provided discussion points regarding the known plasma levels of Ponatinib in patients and the potential targeting of BRAF and ERK signaling inhibition by using Ponatinib in CML or in other tumors with oncogenic BRAF. We also highlighted clinical context that the use of Ponatinib, because of its potential dual targeting for BRAF and specific RTK, may be particularly effective to suppress ERK signaling in tumors.

4. The PDB entry codes for the two crystal structures that are reported in the manuscript should be added to Data availability statement and the SI Table 1.

We now provide the PDB entry codes of the two crystal structures in the Data Availability Statement (main text page 40) and they are also listed in the SI Table 1.

REVIEWERS' COMMENTS:

Reviewer #1 (Remarks to the Author):

The new manuscript addressed all my concerns and comments, thus I think this manuscript should be accepted and no further changes are required.

Reviewer #2 (Remarks to the Author):

Cotto-Rios et al. set out to identify novel RAF dimer inhibitors to be utilized in the context of BRAFV600E resistant tumors and wild type RAF-dependent tumors. One major concerned was identify by the reviewer that related to Ponatinib targeting of BRAF in cells to impact reduce cancer cell growth. The authors conducted additional experiments that now fully address the reviewer's concerns.

Reviewer #3 (Remarks to the Author):

The authors have answered the reviewer comments with satisfaction. The work is novel and of broad interest to the Nature Communications readership.

Oliver Hantschel
